# Making voluntary medical male circumcision services sustainable: Findings from Kenya’s pilot models, baseline and year 1

**DOI:** 10.1371/journal.pone.0252725

**Published:** 2021-06-11

**Authors:** Stephanie M. Davis, Nandi Owuor, Elijah Odoyo-June, Jonesmus Wambua, Eunice Omanga, Mainza Lukobo, Catharine Laube, Zebedee Mwandi, Chutima Suraratdecha, Urbanus M. Kioko, Wesley Rotich, Jacquin Kataka, Caroline Ng’eno, Diwakar Mohan, Carlos Toledo, Appolonia Aoko, John Anyango, Daniel Oneya, Kennedy Orenjuro, Elizabeth Mgamb, Kennedy Serrem, Ambrose Juma

**Affiliations:** 1 US Centers for Disease Control and Prevention, Atlanta, Georgia, United States of America; 2 Jhpiego, Nairobi, Kenya; 3 US Centers for Disease Control and Prevention, Nairobi, Kenya; 4 Jhpiego, Baltimore, Maryland, United States of America; 5 University of Nairobi, Nairobi, Kenya; 6 Center for Health Solutions, Shinda Project, Siaya, Kenya; 7 University of Maryland, Baltimore, Maryland, United States of America; 8 Timiza Project, Migori, Kenya; 9 Health Department, Siaya County, Siaya, Kenya; 10 Health Department, Migori County, Migori, Kenya; 11 National STD/AIDS Control Program (NASCOP), Ministry of Health, Nairobi, Kenya; Weill Cornell Medical College, UNITED STATES

## Abstract

Voluntary medical male circumcision is a crucial HIV prevention program for men in sub-Saharan Africa. Kenya is one of the first countries to achieve high population coverage and seek to transition the program to a more sustainable structure designed to maintain coverage while making all aspects of service provision domestically owned and implemented. Using pre-defined metrics, we created and evaluated three models of circumcision service delivery (static, mobile and mixed) to identify which had potential for sustaining high circumcision coverage among 10-14-year-olds group, a historically high-demand and accessible age group, at the lowest possible cost. We implemented each model in two distinct geographic areas, one in Siaya and the other in Migori county, and assessed multiple aspects of each model’s sustainability. These included numerical achievements against targets designed to reach 80% coverage over two years; quantitative expenditure outcomes including unit expenditure plus its primary drivers; and qualitative community perception of program quality and sustainability based on Likert scale. Outcome values at baseline were compared with those for year one of model implementation using bivariate linear regression, unpaired t-tests and Wilcoxon rank tests as appropriate. Across models, numerical target achievement ranged from 45–140%, with the mixed models performing best in both counties. Unit expenditures varied from approximately $57 in both countries at baseline to $44-$124 in year 1, with the lowest values in the mixed and static models. Mean key informant perception scores generally rose significantly from baseline to year 1, with a notable drop in the area of community engagement. Consistently low scores were in the aspects of domestic financing for service provision. Sustainability-focused circumcision service delivery models can successfully achieve target volumes at lower unit expenditures than existing models, but strategies for domestic financing remain a crucial challenge to address for long-term maintenance of the program.

## Introduction

Male circumcision (MC) decreases men’s risk of acquiring HIV through sex with women by approximately 60% [1–3]. The World Health Organization (WHO) recommends voluntary medical male circumcision (VMMC) as a public health intervention in countries with generalized HIV epidemics and low circumcision prevalence [4], and VMMC is currently implemented as a large-scale public health program in 15 countries in sub-Saharan Africa. Under guidance from WHO and the Joint United Nations Program on HIV/AIDS (UNAIDS), country programs begin operating in a “scaleup” phase which continues until they reach population VMMC coverage targets (currently, 90% among men and boys aged 10–29 years under UNAIDS Fast Track goals [5]), and should then transition to a”maintenance” phase [6], with the new goal of sustaining coverage as new boys enter the target age group, for as long as needed to control the HIV epidemic.

The need to develop service delivery strategies for this maintenance phase is becoming more apparent. Despite current large annual volumes of VMMCs, reaching over 4 million in 2018 [7], the “youth bulge” demographic transition has created an even larger annual cohort of nearly 5.5 million [8] 10-year-old boys across the VMMC implementing countries. Thus, the number of circumcisions needed annually is likely to remain similar through the phase change. While VMMC scaleup is largely donor-supported, with the US President’s Emergency Plan for AIDS Relief (PEPFAR) alone funding 84% of global VMMCs done through 2018 [9, 10], these resources may not be available to meet the VMMC needs of all beneficiary countries indefinitely. Countries therefore need their maintenance-phase VMMC services to become sustainable, and with several approaching their coverage targets, this need is becoming more urgent. Throughout this manuscript, “maintenance” refers to a post-scaleup phase in which the overall VMMC program goal is maintaining coverage levels already achieved; “sustainability” refers to a set of program characteristics, discussed below, expected to be desirable or crucial for continuing to deliver high-quality VMMC services during the potentially indefinite maintenance period.

Conceptual frameworks for sustainability in public health programs already exist, including within the HIV realm. While the need for sustainability from a financial perspective is intuitive, PEPFAR’s HIV service sustainability framework is broader, encompassing programming which is “country-owned and country-driven” [11], in a context where the country “has the enabling environment, services, systems, and resources required to effectively and efficiently control the HIV…epidemic”, with “increasing shares funded domestically over time” [12]. Both the PEPFAR Sustainability Index Dashboard (SID) [13] and WHO’s draft guidance on VMMC sustainability [14] organize sustainability into major domains, with PEPFAR using four (see Methods) and WHO capturing similar divisions with its longstanding concept of the six building blocks of health systems. These conceptual tools were developed for program use and reflect consensus views of major donor leadership and health systems experts on the critical elements of sustainability.

However, despite an emerging interest in sustainability, there is little field implementation experience with VMMC service delivery models intended to be sustainable by clearly defined metrics. Previous work on VMMC sustainability focused on developing national-level VMMC sustainability policy, plans and institutions [15, 16]; and embedding VMMC services in cross referral networks with priority health services [17]. But important questions remain in the service delivery realm; including what age groups to target; what delivery modality or modality mix to use; whether local implementing partners should remain involved or be replaced by Ministry of Health (MoH) staff; whether staff should be dedicated to VMMC alone or perform VMMC as one among other service responsibilities. Finally, because implementing countries have substantial internal variations in key factors like population density and transportation access to health care, no single model may be appropriate throughout the different settings in a given country.

Despite this lack of concrete experience, VMMC sustainability may become more feasible in the maintenance phase. A widely accepted service delivery approach is to continue circumcising rising adolescents. If this approach is taken, service delivery would concentrate on venues like schools, which deliver large numbers of clients per location, rather than stretching resources to reach broader age groups across more diverse venues. The intrinsic high demand for VMMC among adolescents may also lead to decreased need for demand creation and cost saving as circumcision during adolescence becomes the norm.

To address the sustainability experience gaps in a country that has nearly completed its scaleup phase, the US Centers for Disease Control and Prevention, Kenya’s National AIDS and STI Control Program (NASCOP), and the Ministries of Health of Migori and Siaya counties in Kenya, collaborated to organize, evaluate and refine adolescent service models intended to achieve sustainability. Progress towards sustainability was measured using a set of customized metrics largely drawn from the PEPFAR SID and designed to capture a wide range of both standard and novel parameters. The early adolescent age group (10–14 years) was targeted, due primarily to its historical high uptake of VMMC services in Kenya and its efficient accessibility through schools, detailed further below. Here, we describe the approach taken and the findings from baseline and the first year of implementation.

## Methods

### Study design

The full study protocol is available in S1 Appendix. We conducted a prospective pre-post assessment of static, mobile and mixed models each implemented in a purposively selected cluster of wards in Siaya and Migori counties of Kenya for a total of six clusters. Wards are Kenya’s smallest electoral units within each county with a typical population of 10,000–20,000 people. Since program inception, all the three models evaluated in this study had been implemented seamlessly across the country at the discretion of implementing partners but without formal assessment focused on sustainability. Under the evaluation protocol, the target age band for all three models was boys aged 10–14 years. For safety, 10 years was the lower age limit for PEPFAR-supported VMMCs outside infancy at the time. The 10–14 year age band was chosen for this evaluation because of: a) its rapid contribution to maintaining coverage among older, sexually active age groups as the boys age; b) its intrinsically high demand for VMMC, e.g., 60% of all Kenyan VMMC clients were aged 10–14 years in 2017 [10]; c) logistical efficiency of accessing them through schools, given that Kenya has 72% net male primary school attendance [18]; and d) the necessity of circumcising adolescents for at least the next decade regardless of strategy, because even with adoption of an infant circumcision strategy, adolescent MC would still remain available to cover infants past two months who would not safely get MC until they reach the lower age cutoff. In each study cluster, the VMMC services were reorganized to prioritize boys 10–14 years and to be provided solely through one of the three models. Across all study clusters, clients above 14 years who presented for VMMC were offered the service but were neither actively recruited nor counted towards the evaluation targets. Mobilisers focused on activities reaching younger adolescents such as direct meetings with schoolteachers, parents, and village leaders, and leveraging health promotion events planned in the wards by ministry of health team. They were refreshed monthly and their outputs reviewed weekly to ensure fidelity. It was envisioned that exclusive focus on 10-14-year-olds in clearly defined clusters would make it possible to measure any efficiency or program quality gains associated with each model, which are critical for sustainability.

Each study cluster consisted of 2–6 contiguous wards where only one service delivery model was implemented. The clusters were purposively selected through consensus with the county Ministries of Health and the implementing partners operating locally. Factors considered in selection and assignment of models to clusters were: a) to leave ‘buffer’ areas (not implementing any study model) between different clusters; b) to avoid including in the study cluster wards that border areas with large traditionally circumcising populations, from where many adolescents would potentially cross over to seek VMMC as an alternative to cultural circumcision; and c) to include one sparsely populated rural cluster for mobile model, one peri urban cluster with moderately dense population for mixed model and one densely populated urban cluster for static model.

A In the clusters assigned a **‘static’ model**, general clinicians stationed in a static health facility offered VMMC to clients who come requesting it. Outreach and mobile services were suspended. The VMMC teams determined the number and distribution of static sites sufficient to meet all VMMC needs of the cluster. Mobilizers were trained to refer 10–14 year old potential VMMC clients to these sites, as well as any older men who requested the service.
B In the clusters assigned a **‘mobile’ model**, a dedicated mobile VMMC team was responsible for maintaining VMMC coverage over a large catchment area via year-round outreach visits to multiple venues. Teams had a ‘base’ site but followed a set monthly schedule of service delivery in outlying health facilities and community sites within the cluster. Clients aged 15+ years were served at the ‘base’ sites. Clients aged 10–14 years were referred by mobilisers to the mobile/outreach services only.
C In clusters assigned a **‘mixed’ model**, providers offered both static and mobile service models. Teams chose their strategy mix between the two models freely. Adolescent-targeted campaign-style demand creation and services were conducted at opportune times in the school year.

The national routine VMMC data collection tool used in the study clusters was expanded to include evaluation-specific variables such as the ward of residence for each 10-14-year-old client circumcised. The evaluation was based on numerical target achievement and expenditure data plus key informant data in each model area, as well as consensus lessons learned from the investigators and implementers. The broad mix of outcomes was needed to cover the wide spectrum of elements that are potentially important to program sustainability and to explore for additional considerations needed to make VMMC sustainable.

VMMC services were provided by the implementing partners already responsible for these services in the study areas: Center for Health Solutions (CHS) in Siaya, and University of Maryland Baltimore (UMB) in Migori through subgrantee Impact Research and Development Organization (IRDO). These partners were responsible for reporting service delivery and expenditure data from each study cluster. Jhpiego, an international academic partner providing technical support and coordination for VMMC services in Kenya, conducted data collection from key informant interviews and consensus lessons learned from the investigators and implementers as well as analysis. Jhpiego also tracked any administrative markers of the progress of VMMC service transition from implementing partners to the county ministries of health, such as staff employment and inclusion of VMMC in government strategic documents and budgets.

In the analysis described here, primary outcomes in the baseline period (November 2016-October 2017) and Year 1 (actually the 10-month period December 2017-September 2018, truncated to align with the PEPFAR fiscal year) were compared. November 2017 was a planning period falling between the baseline and year one implementation periods.

### Baseline conditions, interventions and data collection

At baseline, all selected study clusters had ongoing VMMC services, provided through delivery approaches selected by partners but typically more similar to the ‘mixed’ model than to the others used in the study. Typically, these involved both year-round services performed by staff at their static facilities and mobile or outreach services provided in tents or peripheral health facilities, respectively. In keeping with national age prioritization, they emphasized recruitment of men 15–49 years [19], but circumcised any age-eligible boy or man on request and often held school-based campaigns.

During the baseline year, November 2016-October 2017, partners collected routine programmatic data regularly reportable to PEPFAR, including client numbers and ages. Notably, the baseline year was also the service startup year under new CDC/PEPFAR cooperative agreements for the partners working in both counties. Partners and the county MoHs also maintained routine internal expenditure records on the county without disaggregation to the study cluster levels. These expenditures captured by activity type using internal categorization systems were reported to the project evaluation team and retrospectively aligned with the study’s activity categories and designated as recurrent vs. investment by the investigators. Recurrent expenditures included administration, medical and non-medical supplies, program supplies, communication, vehicle maintenance and renovations, sensitization meetings and supervision, training of service providers including refresher trainings, salary for contracted staff, transport and personnel costs, utilities (e.g. water bills, electricity bills, etc.), expenditures on technical working groups, allowances and expenditures on County Health Management Teams (CHMTs) and Sub-County Health Management Teams (SCHMTs), supportive supervision, logistical coordination with schools and other sources of adolescents for recruitment, transportation, and backup supply chain support. Investment expenditures included renovating or improving minor theatres in the county health facilities, renovating central sterile services department (CSSD) in the health facilities, building incinerators, improving water supply and management and distribution systems for VMMC commodities, information technology infrastructure, office equipment and medical equipment.

At the study launch in November 2017, each study cluster was assigned one of the three service delivery models targeting boys 10–14, replacing routine service delivery: Each model was implemented by a team operating in partnership with its county health department according to the Kenya national VMMC guidelines [20] and following WHO standards [21]. Each team included a clinical officer or nurse VMMC performing the surgery, an assistant, a VMMC counsellor and an infection prevention officer. Standard national VMMC demand creation strategies were implemented. These included referrals from satisfied clients and peer mobilisers, distribution of education and communication materials in communities and schools, and interpersonal communication approaches to both clients and their parents, all focused on young adolescent clients.

### Data collection and outcomes

Programmatic data was captured using paper-based data collection tools which were later transferred by a trained staff member into a spreadsheet using standard study forms and categories on a monthly basis, and then provided to study coordinators based in the counties. Expenditure data was directly collected by economic study team staff in collaboration with MoH and implementing partner staff retrospectively, reviewing their primary expenditure data together once per project year for the full period. Metrics were collected separately for every study cluster in each county. These, and their collection processes, were:

- *Primary performance outcome*: *Number of VMMCs achieved among 10-14-year-old residents of study clusters*, *as a percentage of the cluster’s annual target*. Client age was collected in routine intake data, and providers were trained to document self-reported client residence (inside vs. outside the study cluster) through chart flagging. This was based on where they reported spending the most nights in the past six months, excluding boarding school. These data, and VMMC numbers, were summarized and submitted through a monthly paper study form for each cluster. Annual targets for study clusters were calculated based on each cluster’s 10-14-year-old male population projected to 2017 from the 2009 national census [22]. The goal was to achieve 90% coverage over a two-year period (essentially completing the scaleup phase in the model areas among the target population), from a baseline of 68% in Migori and 76% in Siaya obtained through expert consensus. Annual targets for all clusters ranged from 1277 to 3302.
  Routine program data from the baseline period was not stratified by client residence, and therefore the comparator when considering changes in overall performance from baseline is the *total* number of 10-14s circumcised in Year 1 (Y1).
- *Secondary performance outcome*: *Percentage of 10-14-year-old VMMC clients who were residents of the model area*. This was collected to provide context, in the event of a cluster performing well numerically but without achieving its coverage targets because a large proportion of clients lived outside the cluster.
- *Primary expenditure outcome*: *Unit expenditure (UE) per VMMC*. The numerator was total VMMC expenditure from all sources of support on all clients, because it was not feasible to attribute expenditures to specific age categories. The denominator was total MCs across all ages, to avoid an artificial UE decline from the increasing proportion of MCs done in 10-14s by Y1. Decreases noted in UE by Y1 were therefore expected to represent a combination of newfound efficiencies and existing inherent efficiencies from targeting adolescents. Activity-specific data were collected in both fine and broad categories, and sources of funds were attributed at baseline and for each cluster and activity. A financial approach was taken where actual expenditures were available; an economic approach was used for equipment already purchased or donated prior to the baseline period.
- *Secondary expenditure outcome*: *Percent of total expenditure in each broad recurrent spending category supplied by the county Ministry of Health*. The fine categories used in initial data collection were collapsed by the local economic coauthors into prespecified broad categories in reporting MoH contributions to percent recurrent expenditure, to more intuitively display which areas MoH was preferentially investing in. Categories were: human resources for routine services; human resources for technical assistance and quality assurance; commodities/ consumables; facility operation, transport, waste management; and demand creation. Annual targets for rising Ministry contributions were set for each category, ranging from 10–80% in Y1 and all reaching 100% by year 5. MOH expenditure that was supported by the IP was collected at the IP level (classed as IP expenditure).
- *Additional expenditure outcome*: Though not prespecified as a major outcome, identification of major expenditure drivers and comparison across models was also of interest in understanding impact of model type on financial aspects of sustainability.
- *Key respondent perception outcome set*: *mean Likert scale scores across all respondents on each question in a given questionnaire*, *covering perceptions of multiple aspects of program sustainability*. Respondents were grouped into three different levels, with a different self-administered anonymous key respondent survey given to each level, after consent. These were: leadership level (National VMMC MoH staff, county-level health leadership, UN agency representatives on the national VMMC task force), project site level (VMMC site staff and managers, facility in-charges), and community level (a school administrator, a teacher, a religious leader, a local leader and five residents per ward). For leadership respondent recruitment, questionnaires were distributed to all staff of county health management teams within the HIV domain and all national MoH program managers. For site-level recruitment at all participating facilities, questionnaires were distributed to facility in-charges at health centers and medical superintendents at higher-level facilities. For community member recruitment, village elders, teachers and church leaders were convenience sampled, one per ward, based on willingness to participate, seniority and accessibility. Thus except for community-level respondents, the study did not target specific numbers but rather sought to obtain a census of all eligible respondents.
  Surveys covered respondent perceptions of VMMC services in the study cluster across the appropriate subset of the four key PEPFAR domains of sustainability for each respondent group. Domains included multiple elements and each element potentially contained multiple questions. Domains are defined and their contents summarized in Table 1; full questionnaires are in S2–S4 Appendices. The Civil Society Engagement subdomain, for example, includes questions about the degree of commitment of diverse community groups to the program, the proactive efforts of program leadership to communicate updates to community groups, and community engagement efforts in planning and evaluating the program. The questionnaires were distributed at study launch for the baseline period, and a year later for the Y1 period. Individual respondents could be the same individuals at both time points if they met the recruitment criteria at both, or could change between time points. Respondents received questionnaires and instruction sheets from study coordinators, who collected the completed questionnaires after two working days. Non-responders were reminded three times, and continued non-response led to automatic replacement. Responses were along a Likert scale from 1 (“never” or “strongly disagree”) to 5 (“always” or “strongly agree”).
- *Additional outcomes*: To gather information on the predetermined policy and strategic metrics of sustainability (summarized in S5 Appendix) based on the PEPFAR SID, the evaluation team also reviewed relevant national and county-level VMMC documents including policies, strategic plans, scopes of work and others.

**Table 1:** Sustainability domains assessed through key informant surveys: Definitions, elements, and levels assessed (selected from PEPFAR SID tool).

Domain name	Definition (“what success looks like”)–PEPFAR SID	Elements	Respondent levels surveyed
Government, leadership and accountability	Host government [is] responsible to its citizens and international stakeholders for achieving planned HIV/AIDS results, is a good steward of HIV/AIDS finances, widely disseminates program progress and results, provides accurate information and education on HIV/AIDS, and supports mechanisms for eliciting feedback […]	Planning and coordination Policies and governance Civil society engagement Private sector engagement Public Access to information	Leadership Project Site Community *(civil society engagement element)*
National health system and service delivery	Host country institutions…constitute the primary vehicles through which HIV/AIDS programs and services are managed and delivered… [and] have achieved high and appropriate coverage of a range of quality, lifesaving…services and interventions. There is a high demand for HIV/AIDS services, which are accessible and affordable to poor and vulnerable populations […]	Service Delivery Human Resources for Health Commodity Security and Supply Chain Quality Management Laboratory	Project Site Community *(service delivery element)*
Strategic investments, efficiency and sustainable financing	Host country government is aware of the financial resources required to…meet its national HIV/AIDS…targets [and] seeks, solicits and or generates the necessary financial resources…and uses data to strategically allocate funding and maximize investments.	Domestic resource mobilization Technical and allocative efficiencies	Project Site
Strategic information	Using local and national systems, the host country government collects, analyzes and makes available timely, comprehensive, and quality HIV/AIDS data (including epidemiological, economic/financial, and performance data) that can be used to inform policy, program and funding decisions.	Epidemiological and health data Financial/expenditure data Performance data	Project Site

Finally, changes in implementation approaches over time were reported through biweekly to monthly calls between study leadership and implementer leadership; and implementers, lead evaluators, and investigators together generated consensus on challenges, refinements and lessons learned through dedicated discussions.

### Data management, storage and analysis

Completed data collection tools were verified by research coordinators and electronically transferred to the database by the evaluation data officer. Monthly routine database backups were automatically scheduled. Paper forms and records were kept under lock and key. A random sample of 10 records per model was verified by the research officer to ensure accuracy of the details captured. Qualitative data was entered into the REDCap software, which was programmed to include logic and consistency checks to minimize data entry errors. Summary statistics were also used to ensure no missing data; any missing data was resolved via calls with persons responsible for the initial reporting. Data was stored by Jhpiego on the Kenya National AIDS & STI Control Programme (NASCOP) encrypted secure database.

Y1 data analysis addressed:

- Performance: Monthly and monthly cumulative achievements among target-aged residents, as a percent of target, were tracked for each model area in Siaya and Migori. Additionally, though not a major performance outcome, mean total monthly VMMCs (total VMMCs performed in all clients during the project year, divided by number of months included in that year) were compared between the baseline and Y1 years. This analysis was done to provide an intuitive check on the reasonability of performance, as a large absolute drop from baseline to Y1 would have required explanation even if performance were adequate against targets. The monthly means rather than annual totals were used to adjust for the different lengths of the two “years” compared, as there were 12 months in the baseline year and 10 in Y1. The comparison used a simple bivariate linear regression with generalized estimating equations with exchangeable correlation to account for clustering at the level of the facility. The p-value generated was for the t statistic testing the hypothesis that the coefficient for the project year is equal to 0, with a significance threshold of p < .05.
- Expenditure: A program-level approach was taken. Key descriptive analyses done on the model-area level included baseline and Y1 unit expenditure, and percent contribution by MoH to expenditure in total and stratified by major expenditure domain. Assumptions used in expenditure estimation in each county, and mapping to the categories displayed in the results section, are in S6 Appendix.
- Key respondent perceptions: For each model area and for all model areas combined, mean Likert scores for each subdomain (calculated as the mean of scores across all questions in the domain) were tested for change between baseline and Y1 using an unpaired t-test for indicators with sample size over 30. For indicators with sample size less than 30, the Wilcoxon rank test was used to test for differences. Descriptive analysis was also done to identify the individual questions with the five highest and lowest scores for each questionnaire at each time point.
- Challenges, refinements and lessons learned: These were distilled from weekly and one-off meeting notes and summarized by the primary investigator.

### Ethics statement

This study was approved by the Johns Hopkins University School of Public Health Institutional Review Board, and the Maseno University Ethics Review Committee. This project was reviewed in accordance with CDC human research protection procedures and was determined to be research, but CDC investigators did not interact with human subjects or have access to identifiable data or specimens for research purposes.

## Results

### Performance outcomes

Targets and performance of study models are shown in Table 2, covering the primary and secondary performance outcomes.

**Table 2:** Performance in total and against targets, by county and model, baseline and Year 1.

Model implemented in Y1	County	Baseline—all MCs in 10-14s	Year 1—all MCs in 10-14s	Year 1—primary outcome: eligible MCs (done in 10–14 yo residents)	Year 1—secondary outcome (among 10–14 yo’s getting MC, % eligible	Year 1 target	Year 1 performance % (eligible/ target)	Year 1 performance (eligible/target) corrected to a full 12-month period	p-value for change: mean monthly MCs in 10-14s, baseline to Y1
Static	Migori	1363	2143	1207	56	2462	49%	59%	0.07
	Siaya	2466	3289	1781	54	2826	63%	76%	<0.001
Mixed	Migori	4462	6945	4790	69	3302	145%	174%	0.23
	Siaya	2746	2499	864	35	1277	68%	81%	<0.001
Mobile	Migori	2359	1837	1419	77	1740	82%	98%	0.03
	Siaya	5369	3374	1459	43	2840	51%	62%	<0.001
**Total**		18765	20087	11520	57	14447			

Performance against targets was higher in Migori than in Siaya in the mixed and mobile models. In both counties, the mixed model outperformed the others against targets. Mean monthly MCs performed in 10-14-year-olds (not shown), compared between project year periods, increased from baseline in the static and mixed models, either significantly despite the shorter year (Siaya) or non-significantly (Migori), but decreased significantly in both counties’ mobile models.

**Fig 1:**
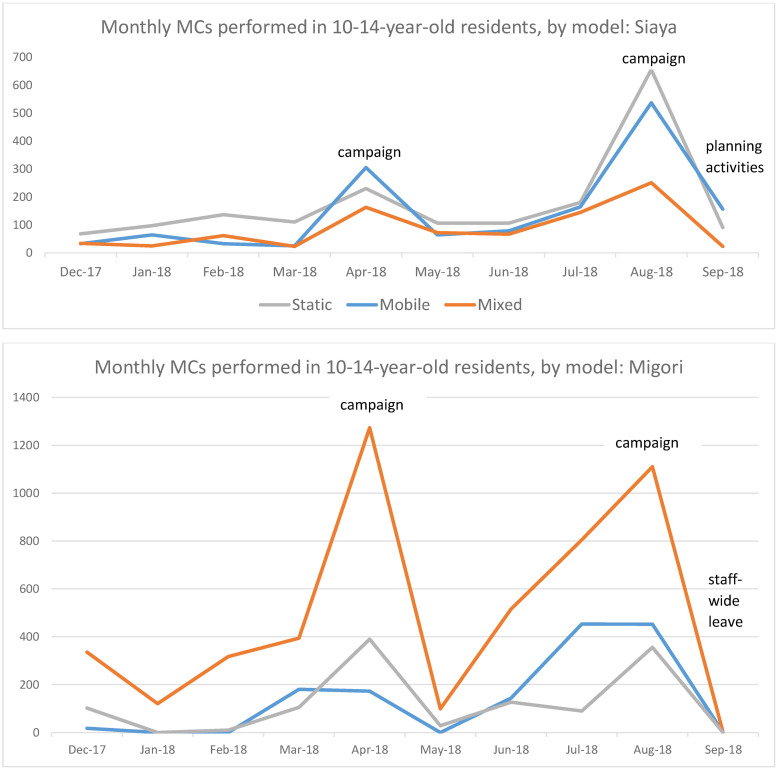
Monthly performance (10-14-year-old residents) by model with key influencing events, Siaya and Migori, Year 1.

Substantial variations in monthly performance over the Y1 period were also noted. Fig 1 shows monthly number of MCs in the target population performed by model in Migori and Siaya, with key influencing events marked. The overall pattern was characterized in both areas by spikes in performance during campaigns in April, August and December, but also by gradually increasing base volume. Performance also dropped at the beginning of the PEPFAR year due to staff diversion from clinical work to planning activities by the implementing partners.

### Expenditure outcomes

Total VMMC expenditure in Migori rose from approximately $906,000 at baseline to $1,009,011 in Y1 despite the shortened year (approximately $1.2 million if corrected for the shortened year). In Siaya it fell from $2.4 million to $2.1 million (approximately $2.5 million if also corrected). Total investment expenditure in Siaya rose from $11,800 at baseline to $32,000 in Y1, all by the PEPFAR-supported implementing partner. Total investment expenditure dropped in Migori from $53,000 at baseline to none in Y1.

Unit expenditures for each model and county in the baseline and Y1 periods are shown in Table 3, along with Y1 volumes, covering the primary expenditure outcome. The key columns for comparison between baseline and Y1 are shaded.

**Table 3:** Volume and unit expenditures at baseline and in Year 1.

Model	County	Baseline overall unit expenditure (total expenditure/ all MCs) *	Baseline unit expenditure per MC in 10–14 (total expenditure/ all MCs in 10-14s)*	Year 1 Volume (all MCs)	Year 1 overall unit expenditure (total expenditure/all MCs)*	Year 1 unit expenditure per MC in 10–14 (total expenditure/all MCs in 10-14s)*
Static	Migori	$57.08	$72.50	2399	$40.92	$49.81
	Siaya	$57.82	$68.01	4173	$88.70	$112.54
Mixed	Migori	$57.08	$72.50	7451	$34.33	$37.21
	Siaya	$57.82	$68.01	2888	$44.45	$51.75
Mobile	Migori	$57.08	$72.50	1943	$124.42	$135.49
	Siaya	$57.82	$68.01	4456	$89.86	$111.65

* Because no differentiated models were implemented at baseline, baseline UEs are county-level.

Baseline unit expenditures were essentially the same between counties. Unit expenditures dropped from baseline to Y1 in the mixed model (both counties) and the Migori static model; they rose substantially in the mobile model and the Siaya static model. Comparison with achieved volumes shows that models with higher volumes had lower unit expenditures, except that the Migori static and Siaya mixed models had low expenditures with low volumes.

Primary expenditure drivers in Migori were personnel (both years), travel (both years), commodities (baseline) and administrative costs (Y1). Primary expenditure drivers in Siaya were personnel, travel and commodities (both years).

**Fig 2:**
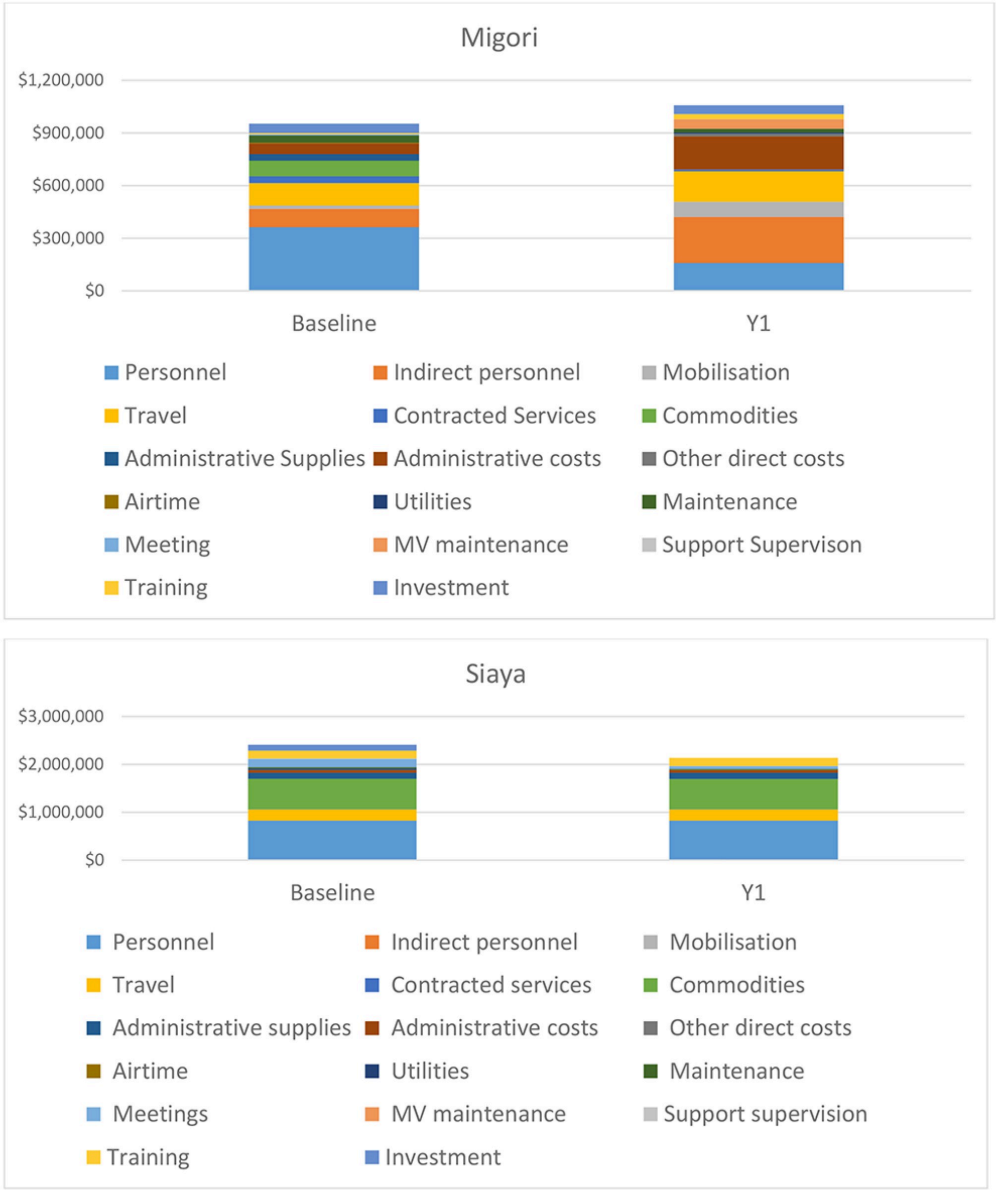
Expenditure breakdowns, baseline and Year 1, Migori and Siaya counties.

Primary Y1 expenditure drivers in the model areas with substantial expenditure increase were personnel (Siaya static) and commodities, personnel and travel (Migori mobile). Primary Y1 expenditure drivers in areas with substantial decrease were personnel and travel (Migori mixed) and personnel and commodities (Siaya mixed). Indirect personnel were a more important cost driver in Migori than in Siaya; in Migori these costs were paid mostly to implementing partner HQ staff, and increased in Y1 as additional HQ support was added, where in Siaya they were paid mostly to county health management team staff. Full expenditure category breakdowns are in Fig 2.

Percents of total expenditures covered by the MoH for each county by category (secondary expenditure outcomes for the study) are shown in Table 4. MoH contributions dropped from baseline to Y1 in most categories and in total, except for small increases in facility expenditures (Migori) and assumption of responsibility for demand creation (Siaya). Total MoH expenditures also dropped. In Siaya they dropped from $147,700 (14.77 million KSh) at baseline to $95,000 (9.5 million KSh); at the same spend rate over a full 12 months, corrected total Y1 expenditure would have been $114,000. In Migori they dropped from $116,000 (11.6 million KSh) at baseline to $62,300 (6.23 million KSh); corrected 12-month Y1 expenditure would have been $66,600. The shortened Y1 period should not affect MoH percent contributions.

**Table 4:** Percent of recurrent VMMC expenditure provided by MoH by spending category, baseline and Year 1.

Expenditure category	Migori		Siaya	
	Baseline	Year 1	Baseline	Year 1
Human resources for routine services	16%	0.3%	0%	1%
Human resources for TA & quality assurance	59%	21%	33%	23%
Commodities/supplies	0%	0%	0%	0%
Facility operation, transport, waste management	0%	2%	9%	4%
Demand creation	0%	0%	0%	100%
Total	13% ($.116 million)	6.1% ($.062 million)	6% ($.148 million)	5% ($.095 million)

### Key respondent perception outcome

Mean key informant response scores for baseline and Y1 are displayed in Table 5, covering the key respondent perception outcome. Mean baseline and Y1 scores were similar across models (maximum differences of 0.64 and 0.61 respectively). Higher scores signify stronger mean respondent agreement with a set of positive statements across aspects of the domain or subdomain.

**Table 5:** Mean key informant response scores across sustainability domains, baseline and Year 1.

Main Domain	Subdomain	Baseline	Year 1	p-value
*County and national leadership level (tool 4)*		*N = 7*	*N = 6*	
Governance, Leadership, Accountability	Planning and Coordination	4.06	3.48	**<0.001**
	Civil Society Engagement	3.86	2.89	**0.002**
	Transparency	3.87	3.5	0.357
*Site level (tool 5)*		*N = 59*	*N = 46*	
Governance, Leadership, Accountability	Planning and Coordination	3.51	3.75	**<0.001**
	Civil Society Engagement	3.12	3.50	**<0.001**
	Transparency	3.36	3.67	**0.025**
National health system and service delivery	Domestic service delivery	3.85	4.07	**<0.001**
	Human resources for health	3.72	3.97	**0.008**
	Supply chain	4.17	4.10	0.584
	Quality management	4.04	4.18	**0.040**
Strategic investments, efficiency and sustainable financing	Domestic resource mobilization	3.55	3.63	0.254
	Technical and allocative efficiencies	3.53	3.93	**0.005**
Strategic information	Performance data	4.29	4.15	0.785
*Community level (tool 6)*		*N = 21*	*N = 12*	
Community engagement and participation	N/A	3.59	3.71	**0.006**
Service delivery	N/A	3.90	4.03	**<0.001**

On the national level, greatest strengths (highest mean individual question scores) for Y1 were in stakeholder understanding of the project goals, communication between the project team and county health teams, routine convening of all stakeholders by county health teams, county health team commitment to maintaining VMMC coverage, and alignment of staffing goals with VMMC targets. Greatest weaknesses were lack of public expenditure reporting, lack of sufficient resource allocation for VMMC in county health budgets, and lack of county health team engagement with civil society for VMMC program planning and feedback. Also notably, this was the only level on which scores decreased significantly in Y1, with respondent assessments of the “planning and coordination” and “civil society engagement” subdomains dropping substantially.

On the site level, for all models, common greatest strengths for Y1 were in reliable regular provision of VMMC services, regular review of service quality against standards, conduction of VMMC without interfering with other health facility services, community acceptability of services, and staff training and certification in service provision and quality improvement. Key weaknesses included lack of diverse community groups committed to success of VMMC sustainability (mixed and static models), and lack of appropriate resource allocation in county budgets (mobile and static models). There was also a perceived lack of task-shifting to the lowest permitted cadre (static model), but this task-shifting was in fact in place. Nearly all subdomain scores increased significantly in Y1, and none decreased significantly.

On the community level, for all models, strengths for Y1 were in ease of timely access to services, communication about services to community and incorporation of community feedback. Weaknesses were in community representation in meetings with the MoH and involvement in the service planning stage. A notable weakness identified at baseline but not in Y1 was in keeping service provision nondisruptive to other community services (e.g., schooldays). Both subdomain scores increased significantly in Y1. Detailed key informant responses are available in S7 Appendix.

*Additional outcomes*: The documentation review found multiple enablers already in place from baseline in both counties. These included, among others:

- documentation of annual targets and division of responsibilities between county and partner staff
- an enabling environment free of policies discriminating against potential clients outside the typical target population
- full task-shifting already in place: of surgical MC from doctors to nurses, and of infection prevention from nurses to high school graduates with 6 months of training in health and infection prevention.
- institutionalized continuous quality improvement practices

### Challenges, refinements and lessons learned

The most important early challenge, across multiple model areas, was aligning demand creation with the target population. Mobilization is conducted not only by implementing partner staff, but also by trained community health volunteers (CHVs) employed by the MoH to perform outreach on multiple topics. The CHVs initially continued their prior practices of mobilizing for VMMC across model area boundaries, including in traditionally circumcising communities. When this was noted after the first month, expansion trainings were held and coupled with field supervisory follow-up visits, to align mobilization work with the study models. This also became the key refinement identified by partners for continued use after the study period: ensuring that mobilizers prioritized adolescent clients from non-circumcising communities to maximize the contribution to increased coverage. Implementers also observed that all models required continued investment in demand creation work. This was true even in the static model, where it had been initially hoped that since services there were at consistent locations and times, this predictability and the intrinsic high demand for VMMC in 10-14s might make continued substantial investment in demand creation unnecessary.

The models’ age focus made close coordination with schools and local Ministry of Education (MoE) leadership crucial. On the leadership level, VMMC program staff engaged with MoE county and subcounty management teams, resulting in school heads adding VMMC promotion messages into existing packages of school-based preventive health talks. On the school level, a pool of interested teachers emerged who committed to be trained and mentored as VMMC champions and went on to lead regular VMMC health talks in their schools. However, stakeholders were unable to codify coordination in a formal Memorandum of Understanding between Ministries, which proved to be a complex process to navigate, requiring national-level engagement.

An ongoing challenge across all models was staff turnover, necessitating frequent trainings and sometimes creating temporary staffing gaps. Similarly, periodic local circumcision campaigns required temporary staff reassignment between or away from model areas, though eligible VMMCs generated by these campaigns were counted toward model achievements. In addition, goal staffing levels initially calculated as sufficient for maintenance-phase services were not always enough to achieve local targets. Model area programs had to actively reassess staffing levels and adjust them several times during the study period.

However, the staffing realm also underwent a crucial sustainability development during Y1. Both county MoHs deliberately made substantial shifts toward ownership of the human resources and management aspects of VMMC service delivery, working with the implementing partners to plan and execute these shifts. The Migori county MoH spent most of Y1 planning this transition, but in Siaya, service delivery staff began the move from IPs into MoH payrolls and oversight structures, reporting to facility in-charges and discharging some additional health responsibilities along with their VMMC work. Partners there continued to provide the salary funding through a sub-agreement with the MoH, but salaries and benefits were aligned with MoH standards. In both counties, county/sub-county health management teams continued working with IP supervision teams to conduct regular supportive supervision activities.

Another crucial advance emerging from the development of these transition plans was that both counties added VMMC to their annual operations plans in 2018. However, these plans are not supported by dedicated budgets, and the lack of domestic financial support for the program was seen by all as the primary obstacle to sustainability. The county health directors also began work with the county health assemblies’ Committees for Health, to legislate and allocate funds for VMMC in the county HIV budgets, to advance domestic financial commitment to the program through platforms such as the next 5-year County Integrated Development Plan. Another avenue identified for increasing domestic contributions was incorporating costs currently borne by partners for types of support services already funded by MoHs for other health care programs—e.g., autoclaving and waste management—into MoH budgets. A third was incorporating VMMC service delivery into provider (especially nurse) job descriptions with associated benchmarks, implicitly increasing domestic human resources investment.

Implementers and MoHs also identified and executed additional opportunities for more efficient service delivery. These included implementing a national policy replacing universal HIV testing of 10-14-year-old MC clients with risk-based testing; shifting surges in service delivery to times of year when 10-14s were free from school; and the national MoH’s restocking sites where its mapping had identified instrument stockouts as an obstacle to maintaining services with VMMC reusable kits.

Finally, some challenges were model- or area-specific. For the static models these included refitting three facilities previously used only as outreach sites with the dedicated space and equipment to support daily VMMC services, in their new role as static sites. Service volumes were constrained until these renovations were completed, after three months. For mobile models, a key challenge was long travel times from base facilities to some service sites, causing service delivery to begin late in the day with clients waiting longer than expected. Finally, in some model areas, medical resources were temporarily diverted due to a cholera outbreak, and in others facility availability was interrupted during doctors’ and nurses’ strikes.

## Discussion

This manuscript compares the baseline and first implementation year for a nonrandomized implementation science study of three VMMC service delivery models intended to progress toward sustainability by multiple metrics, in a country with a mature VMMC program. Key findings included the adequacy and feasibility of all models for a startup year; the standout performance of the mixed model in both achievements and unit expenditure; notable progress toward MoH leadership particularly in human resource areas; and lack of progress so far in the crucial area of financial responsibility. Service sustainability is multidimensional and requires multiple types of quantitative and qualitative indicators to assess. This evaluation collected a uniquely comprehensive set of metrics in an initial effort to achieve this.

With respect to performance against targets, we consider all models to have performed adequately for a startup year, after correction to a 12-month period—including the lowest performer, the Migori static model at 54%. In our view, none should be ruled out as a potential sustainable approach for some regions of Kenya. The general upward monthly performance trends across study clusters and models were reassuring. Absolute numbers achieved among 10-14-year-olds rose from baseline to Y1 in the static and mixed clusters and dropped in the mobile areas, but the calculated targets for those clusters were substantially below their baseline year achievements and the models performed adequately against those targets. The coming addition of Y2 data will provide further clarity.

The complexity of the relationships between prior achievements, model overall achievements and performance against targets results largely from an important clientele feature noted in Y1 that is likely to have also been true for many years previous. Nearly half of clients served, even in the 10–14 age band, were traveling in from neighboring areas, typically traditionally circumcising areas. All areas except Migori static area would have overachieved their targets had their 10–14 achievements all been in cluster residents. In other programs that border on such areas, to what extent this phenomenon is a problem depends on multiple factors. These include the HIV incidence disparity between the circumcising and non-circumcising areas, whether the VMMC resource envelope is sufficient to cover both simultaneously or requires prioritization, and whether local nonmedical MC practices are considered effective in reducing HIV risk, depending on how much of the foreskin is removed. In Kenya, HIV prevalence in the pre-ART, pre-VMMC era was much lower in circumcising than in non-circumcising areas [23], a finding largely attributed to effective nonmedical circumcision practices.

The most notable expenditure finding was the low UE of the mixed model, a promising sign for its sustainability. Though all outcomes are difficult to compare across models because of their purposive geographic area selection, the mixed model areas, with their combination of rural and urban characteristics would not have been expected a priori to be cheaper settings to operate. In addition, the value difference is substantial, and is seen with both the high volume in Migori and the lower volume in Siaya. Mobile models are conventionally considered to have high total costs in VMMC programming due to multiple travel-associated costs, and our findings build on this by confirming high UE as well, at least at the modest volume achieved. By the same reasoning, static models are typically considered cheapest, but our findings are mixed. Siaya’s static model notably had comparable unit expenditures to its mobile model, and higher unit expenditures than Migori despite having higher volumes. An important suggestion arising from these findings, and contrary to conventional expectations, is that static models (often also meant when the term ‘integrated’ is used) should not be assumed to be inherently more affordable or sustainable than others.

Unit expenditures dropped from baseline in both countries’ mixed models and the Migori static model. Drops are probably attributable in part to the maturation of the partners’ programs in both counties from their startup period in the baseline year, but their concentration in the mixed models is informative. A 2016 analysis performed by another Kenya implementing partner on its programmatic VMMC data found an average unit expenditure of $44.21 [24], comparable to the mixed model Y1 findings.

The other key expenditure finding, and the most concerning finding from the study overall, was the decrease in Y1 in MoH contribution to recurrent expenditures, from an already low baseline. The primary MoH contribution category in both counties, human resources for Technical Assistance and quality assurance, was mostly attributed to leadership staff time spent participating in study planning and oversight meetings. Its decrease in Y1 reflected lower time demands as compared to the intensive planning phase at baseline, but the lack of increase reflects lack of committed budgeting. This remains the biggest challenge to program sustainability. The governance processes now underway described above represent the best pathway at present to obtaining domestic financing commitments. These may include taking on waste management and autoclaving costs, and formally committing additional health care worker time to VMMC via job descriptions. Potential other approaches to funding the program, like cost-sharing with clients, have not been explored to date.

A key missing parameter in developing sustainable VMMC financing is an understanding of how long the program will remain necessary or efficient if HIV incidence continues to drop in implementing countries. The extent to which ongoing antiretroviral scaleup alone can suppress transmission under current test-and-treat approaches is controversial and depends in part on how much transmission results from recent infections which are unlikely to be immediately treated. A final consensus set of criteria for achieving HIV epidemic control has not been defined [25], but recent large-scale intensive combination prevention studies have not achieved annual general population HIV incidences below 0.59% [26, 27]. Thus, the future duration of need for VMMC in the general population is currently unclear, making it difficult to form expectations about whether it will outlast donor financing.

Key informant scores on other domains of program sustainability were generally acceptable-to-high and improved from baseline to Y1. Findings here are chiefly useful for reassurance that leadership, implementing staff and community members do not identify critical unexpected gaps. The frequent identification of various aspects of financing and resource distribution is unsurprising given our expenditure findings. Improving civil society engagement was the other dominant theme and deserves concrete inclusion in county-level VMMC planning. In recent years it has also become a major area of focus for PEPFAR, as noted in its annual guidance publications, for similar reasons [28]. The final notable finding was worsening scores from baseline in this and the ‘planning and coordination subdomain’ among national leadership respondents. A possible explanation for this is the observed diversion of multiple county program leadership staff away from VMMC during Y1, in many cases toward other technical areas of the county HIV response.

The lessons learned discussed above include both partner operational refinements and concrete national-level policy changes. Some offer substantial hope that it may be possible to lower unit expenditures further (e.g. by drastically decreasing testing in young adolescents).

Several limitations to this study are notable. The programmatic setting made confounding by client crossover and unmeasured model area characteristics unavoidable. Target-setting was subject to multiple parameter uncertainties, most importantly baseline MC coverage in young adolescents. No recent survey data with sufficient power in this age group was available; the most recent representative data was the 2014 Demographic and Health Survey, which had found total circumcision coverage in 15-49-year age band of 56% on Siaya and 73% in Migori [29]. Targets for the first two years were designed to complete the scaleup phase by reaching 90% coverage in the target population in each model area, but given the uncertainties in some parameters used to calculate them, some may have represented larger proportions of the true unmet need than others, making percent achievements not entirely comparable across models. A forthcoming regional MC coverage survey will provide clarity. In particular, the striking “overperformance” of the mixed model in Migori—where the number of circumcised clients exceeded not only the target but the underlying target population size estimate—is explicable only by underestimation of the target population size, large-scale incorrect designation of nonresident clients as residents, or possibly large-scale VMMC-seeking by underaged clients reporting their age as 10 years. As population sizes were projected forward from decade-old census data, and the 2019 Kenyan census did not provide sufficient geographic granularity to correct them, population estimation is a likely source of error. Authors most familiar with the study setting believe that overestimation of the baseline MC coverage among 10-14-year-olds in the Migori mixed area also contributed. But we are unable to confidently determine what the relative contributions of these explanations are to this outlier finding or, outside of chance, why only this model area’s results were so discrepant.

Unit expenditure analysis may underestimate MoH total contributions by excluding prior investments in facilities, though this is likely mitigated by the fact that donors also invested substantially in facilities in the early years of the VMMC program. Conversely, while we aimed to capture the expenditure incurred for the project implementation, implanting partners and the MoH did have some expenditures—primarily in the human resource areas—for the evaluation itself, possibly caused overestimation human resource expenditures, and particularly the MoH contribution. Much of the MoH leadership time was spent on this purpose. However, most evaluation expenditures were made by the evaluating partner, and thus excluded.

The key respondent surveys were purpose-built and not externally validated, though they were derived from widely-used evaluation materials. Questions in any subdomain may be more relevant and comprehensive for certain settings than others. Another limitation is that implementer observations and refinements, though they represent valuable experience that was a key goal of this study, are necessarily subjective and need corroboration from experience elsewhere. Most importantly, findings here can only very tentatively be generalized elsewhere in Kenya, let alone other VMMC countries.

A substantial change to VMMC programming which fell after the data collection period also impacts the use and interpretation of our findings. PEPFAR discontinued most support for VMMC in boys aged 10–14 in 2020 to ensure client safety based on emerging data, and PEPFAR-supported programs in Kenya now serve only clients aged 15 years and older. Programs that continue serving younger males will therefore find sustainability taking on greater urgency at the same time that close safety monitoring becomes even more clearly crucial. Programs that adopt the 15-year minimum age will eventually, if successful in scaling up, find that most of the uncircumcised population is boys rising into that age range, e.g. the 15–19 years age band, which would then be expected to become the key clientele group for sustainable long-term service. Our experience can perhaps be applied most intuitively to that same age band, particularly those in school who are reachable through campaign-style approaches. Areas with lower male secondary school attendance may need a wider mix of strategies to reach these young men where they are.

The experience from this project suggests that it may be possible to bring down UE substantially compared to current rates when focusing on adolescents. These results also raise questions about whether fully static models are an ideal sustainable approach as often assumed, and have clarified for participating stakeholders some key remaining challenges in making VMMC sustainable in Kenya.

## Supporting information

S1 Appendix Protocol. (DOCX)

S2 Appendix Key informant questionnaires (tool 4: Leadership). (DOCX)

S3 Appendix Key informant questionnaires (tool 5: Project site). (DOCX)

S4 Appendix Key informant questionnaires (tool 6: Community). (DOCX)

S5 Appendix Metrics. (DOCX)

S6 Appendix Key assumptions used in economic analysis. (DOCX)

S7 Appendix Key informant summary results. (DOCX)

S1 Data (ZIP)
