# Supplementary material for: Making voluntary medical male circumcision services sustainable: Findings from Kenya’s pilot models, baseline and year 1
Source: PLoS One. 2021 Jun 11;16(6):e0252725. doi: 10.1371/journal.pone.0252725 (PMC8195380; doi:10.1371/journal.pone.0252725)
Supplement: S1 Appendix — (DOCX) [file pone.0252725.s001.docx]

**Kenya Project IQ Sustainability Program Evaluation**

1. **Project Title:** Identifying Sustainable Service Delivery Models to Maintain Medical Male Circumcision Coverage in Western Kenya
2. **Names of the investigators, co-investigators/co-applicants**

Martin Sirengo, MBChB, MMed, Head of Kenya National AIDS/STI Control Program (NASCOP), is the Primary Investigator and will oversee project implementation.

Kennedy Serrem, MD, MSc, VMMC Program Manager at Kenya National AIDS/STI Control Program (NASCOP), Co- Primary Investigator and will oversee project implementation under overall guidance of the Head of NASCOP (PI)

Elijah Odoyo-June, MBChB, MSc, PhD, provided technical input in protocol development, will provide field oversight of project implementation, including participating in site visits.

Stephanie Davis, MD, MPH, drafted the protocol and will provide ongoing technical assistance to all aspects of project implementation.

Carlos Toledo, PhD, provided technical input on the protocol, will provide additional technical assistance on implementation, and serves as the project officer.

Catey Laube provided additional text to the protocol as well as technical input, and serves as the primary Jhpiego.

Francesca Odhiambo, MBChB, MMed, reviewed the protocol and provided technical input; will provide technical support for the project implementation in Migori County and serve as the primary contact for the University of Maryland School of Medicine- Kenya Programs, the implementing partner for Migori County.

Elizabeth Owino, BSc, will provide technical support for the project implementation in Migori County and serve as the primary contact for Migori County AIDS/STI Coordinating office.

Jacquin Kataka, MBChB, MPH, reviewed the protocol and made technical input, will provide technical support for the project implementation in Siaya and serve as the primary contact for Centre for Health solutions, Shinda project in Siaya.

Isaac Ngere, MBChB, Msc, reviewed the protocol and made technical input, will provide technical support for the project implementation in Siaya and serve as the primary contact Siaya County AIDS/STI Coordinating office.

Vincent Ojiambo Onyango, BA, MA, will provide field oversight of project implementation, including participating in site visits.

Diwakar Mohan, MD, DrPH, contributed to the study design, sampling and data analysis sections.

Mainza Lukobo-Durrell, DrPH, will participate in protocol and methods development, will provide technical expertise during implementation, review of data analyses, conceptualization of publications, and will participate in manuscript writing.

Chutima Suraratdecha, PhD, participated in protocol development and will participate in evaluation.

1. **Names and addresses of any collaborating institutions**
2. US Centers for Disease Control and prevention (CDC), Atlanta USA,
   1. Stephanie Davis, HIV Prevention Branch DGHT/CGH/CDC, 1600 Clifton Rd, Mail Stop E-04 | Atlanta, Georgia 30333 | [smdavis@cdc.gov](mailto:smdavis@cdc.gov), Phone: (404) 718-4776 | Fax: (404) 639-8105
3. US Centers for Disease Control and prevention (CDC), Kenya,

Elijah Odoyo-June, CDC/CGH/DGHT Kenya, Box 606-00621 Village market, Nairobi. E-Mail: [yed0@cdc.gov](mailto:yed0@cdc.gov) ; Phone: +254 724 256 805

1. Ministry of Health Government of Kenya, National AIDS and STD Control Programme (NASCOP)

Martin Sirengo P.O Box 19361-00202, Nairobi, Kenya. Dr. Martin Sirengo. [Head@nascop.or.ke](mailto:Head@nascop.or.ke) Fax +254202710518

1. Jhpiego- affiliate of Johns Hopkins University,

Catey Laube -1776 Massachusetts Ave Washington, DC 20036, Email: [catey.laube@jhpiego.org](mailto:catey.laube@jhpiego.org); Mobile:  +1 202 322 5986 Skype: ca.lama; [www.jhpiego.org](http://www.jhpiego.org)

1. Siaya County Health Department:

Dr. Isaac Ngere, Siaya County AIDS/STI Coordinating office. Epidemiologist PO Box 597, Siaya-Kenya. E-Mail: [ngereisaac@gmail.com](mailto:ngereisaac@gmail.com)**;** Phone +254724963660

1. Migori County Health Department:

Elizabeth Owino, Migori County AIDS/STI Coordinating office. P. O. Box 258, RONGO, KENYA. E-Mail: [elizaowino@gmail.com](mailto:elizaowino@gmail.com)

1. University of Maryland, Baltimore Program (UMB)

Francesca Odhiambo, University of Maryland programs, Kenya. Wood Avenue, Krep Building 6^th^ Floor, Nairobi, 495-00606. Phone +254 733-120379. E-Mail: [FOdhiambo@mgickenya.org](mailto:FOdhiambo@mgickenya.org); [akothf@gmail.com](mailto:akothf@gmail.com)

1. Centre for Health solutions, Shinda project in Siaya.

Jacquin Kataka, | Program Director | +254 735698223, Centre for Health Solutions - Kenya (CHS) | CVS Plaza | Kasuku Road off Lenana Road, 5th Floor | P.O. Box 23248-00100 GPO, Nairobi | Tel: +254 20 271 0077 | Email: [info@chskenya.org](mailto:info@chskenya.org) | Website: www.chskenya.org | Facebook page: CHS | Twitter: @CHSKenya; Mobile: +254 722 377914

**Roles of the evaluating partner and collaborators:**  The evaluating partner will perform monitoring and evaluation of the piloted models to determine the strengths and challenges of each, and identify areas for improvement and course correction over the course of the project. The evaluating partner will not be involved in service provision, which will be performed as routine service delivery by 1) one or multiple existing CDC Kenya implementing partners, following PEPFAR and national practice standards, and 2) the national and county-level Ministry of Health (MoH). Services and the scope of the evaluating partner’s responsibilities are described in the following sections. The MoH was involved in finalization of the models, will oversee implementation by means of the regular reports described in sections VI.C.2, XI.A-B, and will participate in dissemination as per section VII.D.

1. **Funding Source:** This program evaluation is funded under a cooperative agreement between the U.S. Centers for Disease Control and Prevention (CDC) and Jhpiego, titled “Technical Assistance to Provide High-Quality Voluntary Medical Male Circumcision (VMMC) Services to Programs Supported by the President's Emergency Plan for AIDS Relief Grant” (Grant # 5UGGH001469-02). This protocol responds to an Associate Director of Science (ADS) restriction on funding for the April 1, 2016 – March 31, 2017 project period, but the program evaluation will span three years, pending available funding.
2. **Summary**

As countries that implement Voluntary Medical Male Circumcision (VMMC) for HIV prevention approach 80% male circumcision (MC) coverage among 15-29-year-olds, they need to transition to sustainable program implementation models that can maintain high coverage entirely under national leadership and financing. Sustainable models will need to deliver VMMC mainly targeting adolescents 10-14 years. With PEPFAR support, Kenya has been a global leader in achieving VMMC coverage targets and has the potential to be a leader in moving to full country ownership of sustainable VMMC services. An evaluation to determine the optimal VMMC service models for sustaining high MC coverage is proposed.

In the proposed evaluation, PEPFAR-supported implementing partners and the Kenyan Ministry of Health will pilot three potentially sustainable VMMC models in Siaya and Migori counties, and will rigorously assess each model on its ability to attain predetermined levels of sustainability attributes. No single model is expected to be appropriate everywhere, so three models will be developed for three broad settings. These models will all use existing VMMC approaches, but in combinations designed to serve 10-14-year-olds specifically. They are:

1. A ‘static’ model in which general clinicians stationed in a static health facility offer VMMC to clients who come requesting the service.
2. A ‘mixed’ model in which providers offer adolescent-targeted rapid results initiative (RRI)-type demand creation and services at opportune times in the school year and provide services similar to the static model during the rest of the year.
3. A ‘mobile’ model, in which a dedicated mobile VMMC team is responsible for maintaining VMMC coverage over a large catchment area via year-round outreach visits to multiple venues.

Each model will be evaluated repeatedly over time using a set of pre-specified indicators designed to capture a holistic understanding of the sustainability of each model in each county over dimensions including performance, unit cost, MoH leadership, community acceptability and policy development. Detailed costing data will be collected on each model. A time series sampling methodology will be used to identify significant changes in performance for each model over time. Pre-specified thresholds will be used to determine whether they perform sufficiently over the 3-5 year length of project to justify roll out. The national VMMC technical working group and other stakeholders will receive regular reports on the performance of each model.

**Table of Contents**

[**Roles of the evaluating partner and collaborators:** 3](#_Toc484944973)

[**I.** **Introduction and Background:** 8](#_Toc484944974)

[**II.** **Aims of the Program Evaluation and evaluation questions** 8](#_Toc484944975)

[**III.** **Design:** 8](#_Toc484944976)

[**A.** **Refinement process:** 8](#_Toc484944977)

[**B.** **Implementation models**: 9](#_Toc484944978)

[**C.** **Geographic scope and target population:** 10](#_Toc484944979)

[**D.** **Implementing partner/service provider role:** 10](#_Toc484944980)

[**E.** **Transition:** 11](#_Toc484944981)

[**F.** **Targets:** 11](#_Toc484944982)

[**G.** **Role of circumcision devices:** 12](#_Toc484944983)

[**H.** **Additional services:** 12](#_Toc484944984)

[**I.** **Interaction with Existing Services:** 12](#_Toc484944985)

[**J.** **Evaluation:** 12](#_Toc484944986)

[a. Metrics**:** 12](#_Toc484944987)

[b. Tools: 13](#_Toc484944988)

[c. Respondents: 14](#_Toc484944989)

[**c.** Client-level data: 15](#_Toc484944990)

[**IV.** **Participants and Recruitment:** 15](#_Toc484944991)

[A. Facilities: 15](#_Toc484944992)

[B. Respondents: 15](#_Toc484944993)

[C. Clients: 16](#_Toc484944994)

[**V.** **Procedures** 16](#_Toc484944995)

[A. Recruitment Process: 16](#_Toc484944996)

[B. Consent Process: 16](#_Toc484944997)

[C. Implementation: 17](#_Toc484944998)

[D. Data analysis and interpretation: 17](#_Toc484944999)

[1. Sample size and power: 17](#_Toc484945000)

[2. Analysis: 18](#_Toc484945001)

[3. Interpretation: 19](#_Toc484945002)

[D. Handling of unexpected or adverse events: 19](#_Toc484945003)

[**VI.** **Data Custody, Security, and Confidentiality Protections, and Dissemination** 20](#_Toc484945004)

[A. Data Custody 20](#_Toc484945005)

[B. Certificate of Confidentiality 20](#_Toc484945006)

[C. Data Security, Sharing and Ownership 20](#_Toc484945007)

[D. Data Dissemination 20](#_Toc484945008)

[E. Release of Data 20](#_Toc484945009)

[**VII.** **Additional ethical considerations** 20](#_Toc484945010)

[A. Risks of the Program Evaluation 20](#_Toc484945011)

[B. B. Personal and Social Benefits 21](#_Toc484945012)

[**VIII.** **Payment** 21](#_Toc484945013)

[**IX.** **Management** 21](#_Toc484945014)

[A. Oversight Plan 21](#_Toc484945015)

[B. Stakeholder engagement 21](#_Toc484945016)

[C. Personnel Qualifications 21](#_Toc484945017)

[D. Staff Training 21](#_Toc484945018)

[E. PI role 22](#_Toc484945019)

[F. Timeline 22](#_Toc484945020)

[G. Conflict of Interest 22](#_Toc484945021)

[H. Budget 22](#_Toc484945022)

1. **Introduction and Background:**

As VMMC priority countries begin approaching the PEPFAR target of 80% male circumcision coverage among 15-29-year-olds, they need to plan for a transition to long-term coverage maintenance entirely under national and local leadership, oversight and financing. Sustainable medical male circumcision service delivery models that can sustain high circumcision coverage and quality of services do not currently exist. Kenya is a global leader in achieving VMMC coverage targets and has the potential to be a leader in moving to full country leadership and ownership of sustainable VMMC services. Consistent with PEPFAR 3.0’s evolution towards a more sustainable HIV/AIDS response, PEPFAR will support Kenya to develop “affordable, quality, locally owned (managed, implemented and funded)”^[[1]](#endnote-1)^ VMMC models for the counties implementing VMMC.

Such sustainable models will need to deliver VMMC targeting adolescents aged 10-14 years, either long-term or as a bridge to early infant male circumcision, (through adolescent circumcision for the next decade, until the cohort of boys circumcised as infants age into that group, given that male circumcision under local anesthesia is not recommended for males aged > 60 days to < 10 years.) These approaches differ from the current catch-up phase of VMMC: they will be characterized by lower and more consistent volume, diffuse geographic distribution, pre-existing high-potential venues (schools and other programs) to facilitate access to clients, and the high intrinsic demand for VMMC typical among adolescent males. These characteristics make country-based financing more feasible, if the model used is lower-cost and better-positioned to take advantage of them through links to these venues. Finally, because implementing countries have substantial internal variations in characteristics such as population density and ease of access to health care, no single model may be appropriate throughout any country; approaches adapted to the local area may be needed.

1. **Aims of the Program Evaluation and evaluation questions:**

Over three years, through monitoring quantitative and qualitative indicators of VMMC service delivery sustainability for three VMMC service delivery models for 10-14-year-olds designed for different areas of Kenya, to determine if each can maintain adolescent client demand/acceptability, minimize costs and health system burden, optimize program and HIV/AIDS indicator data availability, and facilitate government ownership. The specific evaluation questions to be answered for each model are:

- 1. Can this model deliver high-quality VMMC services that are sufficient in volume to maintain over 80% coverage among 10-14 year olds?
  2. Can this model be sustainably implemented by the Government of Kenya using its own financial, human, and management/oversight resources?
  3. What are the areas of weakness that need to be improved in order to maximize this model’s sustainability?

1. **Design:**

The evaluation will consist of a prospective in-depth qualitative case-study and quantitative assessment of productivity and costs of three different VMMC service delivery models designed for different environments within Kenya. It is hypothesized that each model will be successful in its area of implementation, as compared to baseline measurements of the VMMC services currently provided in the model area.

## **Refinement process:**

All aspects discussed below will be subject to refinement based on experience throughout the life of the project, including but not limited to model design, geographic scope and targets.

## **Implementation models**:

The three models to be evaluated are all within the scope of current VMMC practice in Kenya, and thus are expected to initially have similar costs to current models, but have not been previously evaluated for suitability for delivering long-term services to adolescents. They are:

- - 1. A ‘static’ model in which general clinicians stationed in health facilities offer VMMC to clients who present requesting it, paired with targeted demand creation and mobilization at, and potentially transportation assistance from and back to schools and other adolescent venues. This approach may help minimize cost and burden, but has struggled to maintain volume in the ‘catchup’ phase due to clinicians’ competing responsibilities. However, it might be more viable in the lower-volume maintenance phase. This model may be most appropriate in high-density urban settings where physical access to clinics is not a barrier and many clinics have suitable facilities for circumcision.
    2. A ‘mixed’ model similar to the most common current approach, in which providers offer adolescent-targeted rapid results initiative (RRI)-type demand creation and services at opportune times in the school year, including use of schools as venues and emphasis on demand creation as necessary; and possibly provide services similar to the static model during the rest of the year. This model may be most appropriate in semi-urban settings or those where the adolescent population and demand have substantial season variations, such as areas with multiple boarding schools.
    3. A ‘mobile’ model, in which a mobile VMMC-dedicated team is responsible for maintaining VMMC coverage, including performing mobilization and demand creation, over a large catchment area via year-round short visits to multiple venues. To the extent feasible, the team will still rely on facilities in the catchment area for supplies and storage space to avoid developing a separate supply chain. Linkage to facilities for the purpose of achievement and adverse event reporting, and adverse event management, will be done as per standard national practice with mobile models. This model may be most appropriate in low-density rural settings where physical access to existing static facilities is a barrier and facilities may not have adequate infrastructure or staff to perform VMMC.

Particularly for models in which staff face competing healthcare responsibilities (static and mixed), a facility-level performance-based financing approach consistent with PEPFAR guidance on incentivization may be used to maintain service volume. (For example, a non-coercive output-based reward scheme that benefits the VMMC team as a group, including mobilizers, for achieving pre-determined ambitious service targets).

Before model implementation (baseline), semi -annually in the first year of evaluation, and annually every year thereafter, the evaluating partner will conduct an interim sustainability analysis of all evaluation data at the county level. Models objectively judged to be unsustainable or suboptimal will be discontinued during the life of the project and replaced with one of the other project models, or modified substantially based on local experience. Models will be seriously considered for discontinuation if they meet any of the following conditions without clear opportunities for improvement:

- Service delivery volumes < 70% of annual target
- Unit costs <10% lower than current implementation costs before the start of the model
- Follow up rate < 50%
- Moderate/severe adverse event rate > 5% ** reviewed quarterly
- Other policy or operational consideration (e.g., high facility turnover) that makes implementation of the assigned model inadvisable, based on determination of county government

## **Geographic scope and target population:**

This evaluation will be performed in Migori and Siaya counties, where the models will be implemented. The counties were selected on the basis of being believed to have achieved saturation or near-saturation (80% of 15-29-year-olds circumcised) and having county-level health leadership (county health teams) interested in the project and committed to its goal of transition to full county-level leadership for the best model identified. The target population consists of all uncircumcised males aged 10-14 years living in the selected areas of the selected counties without medical contraindications to circumcision.

Each model will be implemented in each county. Three groups of two to three contiguous wards or subcounties in each of these counties will be purposively selected based on having interest and commitment from facility-level leadership; not being adjacent to traditionally circumcising areas, to avoid confounding from culturally normative demand spilling over into pilot areas for sustainable VMMC; and being an appropriate setting for the model planned. Setting-specific criteria for each model are:

1. Static model: Urban or mixed urban/suburban area with good transportation options such that physical access is not a major barrier to clients living within that area. Health facilities in towns must have structures suitable for providing VMMC independently without major renovations, but may require additional supplies and staff support. This model may perform best in areas which also have higher intrinsic demand.
2. Mixed model: Semi-urban area containing both towns or semipermanent settlements and large rural sections with limited physical access to facilities, such that mobile services are needed in some but not all of the area; or, area where large numbers of adolescents are available for circumcision during periodic set times of year when mobile campaign can be run, and modest numbers are available at other times. Health facilities in towns must have structures suitable for providing VMMC independently without major renovations, but may require additional supplies and staff support. Ideally this will be an area where leadership of schools and other venues is supportive of hosting periodic VMMC services.
3. Mobile model: Rural area with low population density and limited physical access to health facilities, due to large distances and/or difficult terrain. However, must still be accessible to staff vehicles capable of delivering mobile services, e.g. in or near schools. Must be large enough to justify the use of a full-time mobile team for year-round services, or else mobile team must be hired for only a set period or periods of the year. This area may also have lower intrinsic demand than other areas. Ideally this will be an area where leadership of schools and other venues is supportive of hosting periodic VMMC services.

All facilities in each ward group will be allocated to the model to which that ward group is assigned. Thus each county involved will host all model types, to prevent confounding by county-level variables. (In the case of the ‘mobile’ model, facility involvement may be limited to providing storage space and supplies for the mobile team, as well as attending to complications and concerns from the client in the interim period between the procedure and scheduled follow-up visits.)

Facilities involved in this evaluation will be exclusively MoH facilities, supported in part by CDC-funded implementing partners. Models with potential for low volume output (static, outreach) will not represent more than about 20% of any county’s population, to minimize risk that pilot failure could jeopardize county’s ability to meet overall COP targets.

## **Implementing partner/service provider role:**

This will vary with the model being piloted. For the static model, implementing partner roles will include human resources supplementation for both service delivery and demand creation as needed, supportive supervision, logistical coordination with schools and other sources of adolescents for recruitment and transportation, and backup supply chain support where necessary. For the mixed model, implementing partner roles will be similar, but human resources and logistical support may surge during RRI-type periods. For the mobile model, the implementing partner may initially be wholly responsible for all aspects of service delivery, as it is not associated with specific facilities or MoH services, but will transition ownership to the county leadership as described below.

In all cases, implementing partners will provide any of these and additional elements determined to be necessary through their needs assessments, but will continually seek to capacitate county and facility-level leaders and providers to take over increasing responsibility, consistent with PEPFAR guidance for improving program sustainability.

## **Transition:**

Over the course of the evaluation, any responsibilities currently performed by PEPFAR, including any elements of human resources, supply chain, financial support, and all other responsibilities, will be transitioned to the MoH (see Table 1). While the evaluation is scheduled to take three years, an additional two years will be available for completing transition of implementation funding responsibilities, and the evaluation could be extended to cover those years if funding becomes available and unanswered questions remain about the models’ ability to provide sustainable services. During this evaluation, calls or meetings between CDC, county MoH staff, and Jhpiego will be held every six months at minimum to discuss progress and address challenges.

Table 1: Distribution of **financial** responsibilities by project year (average across all 3 models; expected to vary by model, with early MoH contributions higher in the static and outreach models and lower in the mobile model)

|  | **Year 1** | | **Year 2** | | **Year 3** | | **Year 4** | | **Year 5** | |
| --- | --- | --- | --- | --- | --- | --- | --- | --- | --- | --- |
|  | MOH | PEPFAR | MOH | PEPFAR | MOH | PEPFAR | MOH | PEPFAR | MOH | PEPFAR |
| **Human resources for routine services** | 10% | 90% | 30% | 70% | 50% | 50% | 80% | 20% | 100% | - |
| **Human resources for technical assistance & quality assurance** | - | 100% | 20% | 80% | 30% | 70% | 50% | 50% | 100% | - |
| **Commodities/ consumables** | 40% | 60% | 70% | 30% | 80% | 20% | 100% | - | 100% | - |
| **Facility operation, transport, waste management** | 80% | 20% | 80% | 20% | 90% | 10% | 100% | - | 100% | - |
| **Demand creation** | 50% | 50% | 75% | 25% | 90% | 10% | 90% | 10% | 100% | - |

## **Targets:**

Participating facilities will provide VMMC for all clients meeting eligibility criteria who present for services, but will focus on mobilizing males aged 10 – 14 years. Aspirational targets in the first two years of implementation will be equal to the sum of the number of VMMCs needed to achieve saturation in the 10-14-year age group if not already achieved; plus the number of rising 10-year-olds entering the target age range. Subsequent targets, if initial targets are met, will be equal to the number of rising 10-year-olds. At this point each model will have an annual service delivery target of approximately 2,000 VMMCs, with final targets to be determined by the final choice of subcounties.

## **Role of circumcision devices:**

The PrePex device has been approved for routine use on Kenya for males ages 13 years and up, and may be made available in the program models once providers are trained and conditions for full compliance with tetanus toxoid vaccination protocol are met. ShangRing device is also undergoing evaluation for possible introduction into the national VMMC for circumcision of males 13 years and above. Introduction of any device (PrePex or ShangRing) to the program models will be done uniformly and in full compliance with national guidelines across models to preserve comparability.

## **Additional services:**

- 1. Consistent with Kenyan MoH policy and WHO recommendations, clients undergoing MC through PrePex will receive 2 doses of TT 4 weeks apart and wait for a minimum of 2 weeks after the second dose before device placement. This approach may be adjusted if WHO recommendations or Kenyan MoH policy on tetanus vaccination in the context of VMMC is revised.
  2. The VMMC platform will be made available to other MoH health activities if county leadership decides use it as an opportunity to target other services, such as screening (e.g. for depression, under-age drinking, drug abuse prevention services, domestic violence and unintentional injury risk, and/or other reproductive health services), or condom use education. This may be achieved through linkage to and/or co-location with offered services. Currently, there are no guidelines for additional routine health care services provided as part VMMC package other than TT immunization.

## **Interaction with Existing Services:**

Areas selected for the evaluation are intended to have near or above 80% coverage of male circumcision among men 15-29 years of age, so that additional services in the area targeted at males above 14 years would be minimal and not interfere with assessment of model performance. However, due to difficulties with assessing coverage and/or continued high demand, it may still be necessary for the implementing partner to continue such services in the evaluation area. In such cases, the implementing partner will work with the evaluating partner and CDC to minimize client crossover between the model and other services, primarily by directing demand creation and services for these older males to venues and hours congenial to them rather than to adolescents. Also in such cases, the evaluating partner will include in its quarterly data collection the age distributions of clients in both service types, so as to clarify the extent and impact of client crossover.

## **Evaluation:**

### Metrics**:**

Appendix A is a set of pre-specified indicators designed to capture a holistic understanding of the sustainability of each model in each county over multiple dimensions. Source documents used in developing these indicators included the PEPFAR Sustainability Index Dashboard (SID) 2.0^[[2]](#endnote-2)^ (which provided the basic domains for evaluation), the World Bank Checklist for Transition Planning of National HIV Responses,^[[3]](#endnote-3)^ and Oberth and Whiteside’s 2016 AJAR article “What does sustainability mean in the HIV and AIDS response?”^[[4]](#endnote-4)^

These indicators will be used to track each model’s progress over time. They are designed to contain both process and outcome indicators of sustainability. Both are expected to be lacking initially and to develop over time. Not all indicators are equally under the control of the project implementation staff, but all should help to provide an overall picture of the sustainability of the piloted models.

### Tools:

Data on indicators will be collected as follows (see Appendix B for tools) for the duration of the evaluation (3-5 years depending on funding):

- - 1. Monthly quantitative indicators for program outcomes/outputs (Tool 1): This tool will be filled out for each involved site, is dedicated to abstracting program performance data from existing data streams, and will collect key indicators: the number of VMMCs performed, disaggregated by age, and the number of moderate and severe adverse events. These data will be reviewed quarterly, more frequently than the rest of the project indicators, to allow the evaluation partner to promptly identify and prioritize potential spillover, safety/quality concerns, or other aspects of service delivery under the three prescribed models that will require close attention at the time of annual sustainability assessment. Safety/quality concerns will be brought to the attention of implementing partner staff for immediate resolution, and if they meet the potential model discontinuation criteria described in section IV-B, that model will be seriously considered for discontinuation if there are not clear opportunities for resolution.
    2. Annual quantitative indicators for program outcomes (tool 2): One copy of this tool will be filled out for each model in each county. Program outcome indicators will be the annual county-wide number of VMMCs performed, to assess population coverage. Beyond procedure data, this tool will capture additional objective measures of sustainability that can be ascertained through document review, such as existence of a county VMMC strategy with clear targets.
    3. Annual cost data collection of all VMMC models (tool 3): Data will be recorded for each model in each county. This tool is designed for baseline and prospective cost analysis from provider perspective using the top-down and bottom-up (ingredients-based) approaches. Costs of the services incurred will be based on the spending during the implementation will be collected by phase (development, start-up and implementation), implementation activity (e.g., VMMC procedure, demand creation, outreach, communication, monitoring and evaluation, and quality improvement), input type (recurrent costs [personnel, travel, supplies, drugs, building, utilities, contracted services] and investment costs [training, equipment, buffer stock and new construction and renovation, IT infrastructure), and source of funds at baseline and for each VMMC service delivery model. Worksheets, manual and training will be provided to IP and MoH on how to derive the costs for each input using the ingredients-based and top-down approach. Additional data to be collected will also include the type of equipment purchased and when it was purchased, building size, type of training, for example. Prospectively, the tool will be completed by a trained staff who will work closely with the implementing partners and ministry of health to validate all inputs, costs, and resources for each model. The shared costs will be estimated for each activity and input type either by the percentage of usage (e.g. square foot for building and security) or time used to perform the activity. In-kind contribution will also recorded and its costs will be estimated to allow an accurate assessment of resource needs. Points of contact will be identified for IP program activity detail expertise, IP spending data, MoH program activity expertise, and MoH spending data. The evaluating partner will establish working relationships with these points of contact to oversee the annual cost tracking and to manage the annual validation and cost data collection for activities associated with each VMMC model, in each county, and through the phases of project development, start-up, and implementation. All data that is tracked for each model of VMMC will be summarized and reviewed annually with the use of Tool 3. Data sources will include financial records, program detail data from key staff interviews about personnel time and value of shared resources, in-kind contribution data, local market prices, equipment life expectancy, and capital investments. The following cost components will not be considered in this study:
- Indirect costs (i.e., productivity losses) associated with disease morbidity and mortality, and adverse events costs associated with care and treatment.
- Other societal costs, such as the costs of providing general medical training for health professionals participating in HIV/AIDS programs.
- Higher-level overheads borne by United States Government (USG) agencies to support the interventions.
- Costs associated with research activities occurring at sites but not part of the regular package of care and treatment services.

The ‘mapping’ tab on tool 3 which directs partners on how their fine activity categories are assigned to the broader activity categories used for reporting may be edited over time to reflect changes in fine activity categories being implemented.

- - 1. Annual self-administered **VMMC sustainability assessment tools** (tools 4-6): These are self-administered questionnaires for different groups of respondents, grouped by which domains of model sustainability they would have the opportunity to observe in their work, including evaluating partner staff who will fill it out based on their participation in national and county-level task force meetings and other interactions with stakeholders. Questionnaires have questions developed to obtain respondents’ opinions on each indicator, using a Likert scale. They will be filled out semiannually in the first year, and annually afterwards. Individuals who remain in the same roles for multiple years will be sampled multiple times, though they remain anonymous and therefore unlinked; where there is turnover, new individuals in the same roles will be sampled.

### Respondents:

Respondents include both VMMC staff and community members, further described in the sub-bullets below. They are grouped into the below categories based on the tools they will complete and the domains/sustainability indicators (see Appendix A) they will be expected to address.

Respondents will be selected based on consultation with the national and county MoH, CDC, and other local stakeholders as needed. Over the course of the evaluation, staff turnover or reassignment is expected, therefore the individual respondents may change, but the categorical representation will be preserved.

- - 1. National VMMC leadership, County-level health leadership, other stakeholders (UNICEF and WHO reps on national VMMC task force) and Site MoH leadership at each site: Tool 3
       1. Governance, Leadership and Accountability
    2. Staff directly involved in sustainability model oversight (including USG and MoH staff), site program leadership (VMMC site manager), VMMC site staff, and CDC VMMC lead: Tool 4 containing the domains below, and will assist the evaluating partner in filling out tools 1 and 2 as appropriate based on their access to the necessary data.
       1. Governance, Leadership and Accountability
       2. National Health System and Service Delivery
       3. Strategic Investments, Efficiency, and Sustainable Financing
       4. Strategic Information
    3. Non-VMMC site staff (site staff not directly involved in VMMC) and community members and stakeholders (4-6 residents, teachers and school administrators): Tool 5
       1. National Health System and Service Delivery (relevant indicators only)
  1. Client-level data:

No client-level or personally identifiable information will be collected by the evaluation. However, secondary data from facility records of VMMC clients will be used to show the age disaggregation of clients and other basic demographics, essential for the evaluation of services (see tools).

1. **Participants and Recruitment:**

The static and mixed models will include health facilities that have trained service providers and are approved to provide VMMC services in Migori and Siaya counties. Service delivery will be as per the national standard and the evaluation will not assess the criteria for clinical decisions.

## Facilities:

- 1. **Inclusion criteria:**
     1. Facilities with established VMMC service delivery operation for a minimum of 12 months (static and mixed models only).
     2. All facilities falling within the chosen geographic areas for the model to be employed.
     3. Facilities with at least two trained VMMC providers present (so that services could continue at a basic level even if staffing were not further supplemented by implementing partners); at least one of these, except in the case of the mobile model, should be an MoH staff member.
     4. Facilities with appropriate supplies and equipment for the service delivery model they are implementing, per national guidance; or facilities which can readily acquire these with support from the implementing partner and face no other serious obstacles to offering services.
     5. Facilities with leadership willing and able to provide the service delivery model to be evaluated in their area.
  2. **Exclusion criteria:**
     1. Facilities not approved by the MoH or not willing to be part of the project
     2. Initially, all facilities meeting other criteria in a model’s geographic area will be included; however, over time, if it proves infeasible or inefficient in the static and/or mixed models to continue supporting some low-volume facilities (<3 clients per day on average), and MC coverage and access to MC for all target residents of the area can be maintained through other facilities, partners may shift support away from these low-volume facilities.

## Respondents:

Key informants include:

- - 1. National VMMC leadership, County-level health leadership, other stakeholders (UNICEF and WHO representatives on national VMMC task force) and Site MoH leadership.
    2. Staff directly involved in sustainability model oversight (including USG and MoH staff), site program leadership (VMMC site manager), VMMC site staff, and CDC VMMC lead
    3. Non-VMMC site staff (site staff not directly involved in VMMC) and
    4. School administrators and teachers - the headmaster at each involved school and all teachers whose classes have been involved, and
    5. Community members - 4-6 adult residents with knowledge or experience with VMMC services will be recruited from villages in the involved areas.

The number of key informants to be surveyed will depend on the number of wards, subcounties or facilities selected based on having interest and commitment from facility-level leadership.

## Clients:

Because VMMC provided by the models is performed as routine service delivery, inclusion and exclusion criteria for VMMC service are the same as under the national program.

- 1. **Inclusion criteria:**
     1. Male age 10 years and up (age and residence are not exclusion criteria for service, but males aged over 14 years or living outside selected counties will not be counted towards targets)
     2. Assent provided by client
     3. Written consent provided by client or if under 18, guardian, as per Kenyan law
  2. **Exclusion criteria:**
     1. Medical contraindication to circumcision
     2. Already circumcised

1. **Procedures**

## A. Recruitment Process:

1. Facilities: In collaboration with NASCOP, the national technical working group, and county-level health authorities, sites in Migori and Siaya counties with high circumcision demand and active current services will be selected for evaluation. The evaluating partner(s) will collaborate with county health management teams to select wards for each model, and then identify health facilities within those wards meeting inclusion criteria. All such facilities located within the selected wards will be notified of their potential selection and the evaluation criteria and objectives, including the evaluation questions targeted towards the county health department.

2. Respondents: Respondents will be selected and recruited based on their involvement with the VMMC program. Letters will be sent to national VMMC task force members, chief medical officer and VMMC site manager at each involved site, the CDC VMMC lead, the headmaster at each involved school and all teachers explaining the evaluation and inviting them to participate in the evaluation.

Adult community members with knowledge or experience with VMMC services provided in their community will be invited to complete a self-administered questionnaire (tool #6). Six community respondents will be recruited from members of the health facility management committees, teachers and parents association (PTA), county assembly health committee and provincial administration who will have been sensitized on the sustainability evaluation during stakeholder engagement.

## B. Consent Process:

1. The evaluating partner will facilitate obtaining written agreement from each facility indicating their willingness to participate. The agreement/consent will follow standard communication between the county health department and the health facility administration. The inclusion and exclusion criteria will be included in the letter of communication that discussed selection of facilities.

2. Respondents will be asked to volunteer to complete a self-administered questionnaire. Respondents can decline to complete the questionnaire.

3. Clients will consent to VMMC as per standard MoH procedures using the standard national VMMC consent form. Under Kenyan law, consent for surgery on minors (below 18 years) is given by parents or guardians. For minors residing in boarding schools, responsible teachers convene parents-teachers association (PTA) meetings in presence of the VMMC implementing partner. During the PTA meeting, all stakeholders are sensitized by the implementing partner on the benefits of VMMC and parents/guardians are invited to give written informed consents for minors who opt for circumcision. Written parental or guardian consent must be filed and verified by VMMC providers before any minor is circumcised in the context of outreach to schools.

## C. Implementation:

1. *Model Implementation*: This will follow protocols described in PEPFAR’s best practices documents for the delivery of services. Each facility will be supported through existing implementing partners to ensure adequate:
   1. Workforce
   2. Equipment and supplies
   3. Demand generation plan and staffing
   4. Quality improvement plans

This will begin with a needs assessment for each facility, jointly performed by implementing partner and evaluating partner representatives at the beginning of project implementation. This assessment will determine which of these areas need partner support and to what extent in each facility, and will form the foundation of the implementing partner’s initial support plan. Facilities that currently provide services using more than one service delivery model will alter their process to provide only one type of service delivery model during the evaluation period in order to allow for comparison of variables specified in matrices in Appendix A.

The implementing partner will present a proposed workplan for each model by May 15, 2017, including needs assessment at facilities, plans for mapping and liaising with schools and other venues, demand creation plan, year-round service delivery schedule, and level of planned staffing support. Models will be finalized with CDC Kenya and CDC Atlanta by June 30, 2017. The needs assessment will be updated and resubmitted to CDC Kenya by the implementing partner every six months, and other plans will be updated and resubmitted to CDC Kenya annually.

1. *Evaluation Implementation*: The evaluating partner will encourage the participation of stakeholders including a NASCOP representative, CDC, and county health teams on site visits, to enhance their ability to provide informed opinions through the self-report tools. On an annual basis, the evaluating partner will also perform expenditure analysis for each model in each area, and a catchment area visit to assess whether key venues are being used.

Evaluating partner staff will also attend all county-level VMMC task force meetings, maintain a presence on the national VMMC Task Force, and participate in updates to that body on model progress. The information and analysis obtained will be provided by the evaluating partner in a written quarterly report to CDC, and disseminated by CDC Kenya to other stakeholders including the county health teams as appropriate.

No human biospecimens (blood, urine, saliva, etc.) will be collected.

## D. Data analysis and interpretation:

### 1. Sample size and power:

The implementation will follow a time series sampling methodology with the sample size based on the number of time points for which the data is available. For the purposes of this evaluation, the data time point is the volume of VMMC clients processed through each service delivery model. In each model in each county, a total of approximately 4 facilities serving a combined total of 2000 clients per year, and a total of 13 county-specific key informant respondents is expected. In addition, on the national level, 4 key informant respondents are expected.

### 2. Analysis:

Data collected through both self-administered surveys and secondary data abstraction will be captured in the evaluation tool.

VMMC service statistics collected using Tool 1 will be analyzed using segmented regression methods. The outcome variables of interest include number of VMMC clients, percentage of annual target achieved, age distributions of clients, and adverse event rates. The time scale for the analyses will be a ‘calendar month’. The usage of time series models is twofold – i) Assess the impact of the proposed intervention(s) on the outcome at the ‘adequacy’ level, ii) Forecast future levels of the outcome with present level of intervention. We will begin with exploratory data analysis using scatterplots and trend charts to identify the underlying trend, seasonal patterns and outliers, if any. More traditional descriptive analyses, such as summaries and bivariate comparisons between the outcome and potential time-varying confounders, as well as simple before-and-after comparisons. Segmented regression methods will be used to model the effects of the different phases of the study. The following equation expresses the analysis in

Y_t_ = b_0_ + b_1_ * time_t_ + b_2_ * intervention_t_ + b3* time after intervention_t_ + et

Yt is the total number of clients at participating facilities per month t; t is a continuous variable indicating time in months at time t from the start of observations; intervention1 is an indicator for time t occurring before (intervention = 0) or after (intervention = 1) start of intervention. The error term et at time t represents the random variability not explained by the model and consists of a normally distributed random error and an error term that may be correlated to errors at preceding or subsequent time points

The data will be assessed for trend, variance over time, autocorrelation and seasonality. Forecasting future trends of the outcomes will be done using moving averages and smoothing methods (simple or exponential).

Data collected through self-administered questionnaires (Tools 4-6) will be analyzed using parametric tests such as Chi-square or nonparametric tests such as Mann-Whitney and Kruskal Wallis rank test as appropriate for Likert scale data, depending on underlying distributions.

Data will be presented using frequencies and proportions for categorical variables, means and standard deviations for normally distributed continuous variables, and median and interquartile range for continuous variables with skew. Disaggregation of results will be by model and county. Counties and facilities will be given codes for data collection and analysis. No personal identifiers for any client will be collected or used for analysis.

Qualitative data will be thematically coded. Thematic data will then be analyzed to determine patterns in opinions and experiences of the respondents.

Cost Analysis: A key aim is to understand the financing of VMMC service delivery for Kenya’s Ministry of Health to ensure sustainability of services. The financial and economic cost analysis will be conducted prospectively from the programmatic perspective to assess costs to the health system/MoH and implementing partners. The costs of activities involved in implementing each different model are critical to the Kenya government to assume the financial responsibility for VMMC. From the economic standpoint, the costs of capital investments (equipment and construction/renovation) will be depreciated over the estimated cost of each item life, with a discount rate of 3%. All cost data will be summarized annually and reviewed with program outcome data to generate unit costs against the progress toward transitioning financial responsibility targets. In addition to total costs and unit costs – to be calculated using the number of procedures performed as collected in Tool 2 – the information on cost drivers for each model, the data collected with Tool 3 will provide indicators of shared financial responsibility (proportionate contribution of the MoH both overall and by activity) to inform the discussion regarding program financial sustainability. Cost drivers will be assessed and a sensitivity analysis will be conducted for input types, such as personnel costs, transport costs and beneficiary volume. Using the information on the sources of financing, financial responsibility targets and unit costs by input type, the costs of transition will be projected taking into account potential scale-up scenarios of the models.

Limitations, sources of bias and corrective measures: The major potential limitation and source of bias is external factors which could affect performance of the models, including funding limitations, civil unrest, and protracted health workers’ strikes. These factors will be noted in reports on model performance and may serve as a basis for temporarily continuing a model which otherwise meets criteria for discontinuation, if there is a reasonable expectation that the external factors are transient. Ultimately, ability of the county-level MoH to mobilize and commit the necessary resources to sustain the models is also outside the control of the investigators, and if this cannot be achieved then the project will only be able to provide data on the other elements of sustainability, including techniques for cost minimization, which should still be valuable local implementation. Successful models also cannot necessarily be expected to be successful outside the areas for which they are developed, and will not be expanded elsewhere without careful consideration of local differences which might require adaptation.

### 3. Interpretation:

For each model, in answering the first evaluation question, investigators will determine whether the annual evaluation metrics (Appendix A) are either improved from the prior year or optimal in the majority of metric within every domain (e.g., National Health System and Service Delivery). In answering the second and third questions, investigators will identify which individual metrics are suboptimal and not improving. ‘Optimal’ is defined as:

- For each Y/N metric, a score of 85% ‘yes’ among respondents
- For target achievements, >90%
- For AE rate, < 2%
- For unit cost, lower than $45 (only to be included in evaluation after year 1, to allow for the start-up period; FY2016 PEPFAR national UE = $48.77, which is a reasonable comparison because currently IPs assume virtually all costs of VMMC service provision, using similar models.)

## Handling of unexpected or adverse events:

Adverse events in clients will be handled according to existing national practices, including management according to adverse event algorithms, referral to higher levels of care where indicated, and reporting to PEPFAR and the MoH. Further detail is in the Adverse Event Action Guide, available online at malecircumcision.org. No breaches of confidentiality are anticipated because no individual-level data will be collected on VMMC clients, and respondent data will be anonymous.

1. **Data Custody, Security, and Confidentiality Protections, and Dissemination**

## Data Custody

Data collection forms will be limited to the tools shown in Appendix B, which collect no personal identifiers; data are anonymous. Data will be collected and managed using Jhpiego-owned tablets with Epi Info or MS Access software. To ensure data quality, data quality checks will be built into the data collection software and data will be electronically double-entered by evaluating partner staff. Data will be stored on a secure Ministry of Health server in Nairobi, protected by limited access and strong password systems. Monthly routine database backups will be automatically scheduled. The MoH (NASCOP) will own the data. The data will be owned and retained by MoH and will be disposed of according to MoH procedures and timelines for data destruction (7 years for written records, indefinite storage for electronic records).

## Certificate of Confidentiality

No data will be stored in the United States. Evaluating partner staff will have access to client records in order to verify aspects of service quality delivery, but no individual-level data will be collected from these records. Evaluating partner staff filling out tool 1 will sign a confidentiality agreement (tool 8).

## Data Security, Sharing and Ownership

PIs will be accountable for responsible stewardship of data and protecting data confidentiality. This responsibility includes protecting physical custody of the data, storage and sharing with appropriate data use agreements that contain the appropriate security provisions. The Kenya National AIDS & STI Control Programme (NASCOP) encrypted, secure database will serve as the primary repository for evaluation data. MoH will own all data collected under the evaluation. Evaluation data will be viewed by NASCOP, MoH, CDC, and the evaluating partner for the purposes of project monitoring and analysis as described in Section VI.

## Data Dissemination

In addition to the oversight plans described above, a final report on the performance of the models, with recommendations, will be prepared for NASCOP and the VMMC task force. The stakeholders previously discussed will be invited to join the VMMC task force at a results presentation session, at which their feedback will be obtained. If results are of interest for publication and the MoH is in agreement, they will be submitted with MoH participation for publication to a peer-reviewed journal and at a relevant international conference.

## Release of Data

The recently updated CDC data policy will be followed. The project plans to release evaluation data in an open, machine-readable, non-proprietary format within 30 months of the end of data collection. Data collected will be anonymous feedback on VMMC service delivery and no individual level data will be collected.

1. **Additional ethical considerations**

## Risks of the Program Evaluation

The project itself has no associated risk for clients, as VMMC services will continue to be provided to national standards in facilities that currently offer them, to all clients currently eligible under the national program. If for any reason a client prefers to be served through a model that is not available at the facility of their initial visit, he will be referred based on availability. Adverse events occurring within facilities participating in this program evaluation are not within the scope of this protocol, but will be managed according to the national standard of care and reported through routine reporting channels. Adverse event rates will also be monitored as part of this program evaluation (see Section VI). VMMC programs are culturally acceptable in the selected counties as evidenced by their success to date; no social harm is expected to clients obtaining VMMC at the most common and accepted age for doing so.

## B. Personal and Social Benefits

The evaluation will not create direct personal benefit for individual VMMC clients; rather, this evaluation will produce important information to inform sustainable service delivery approaches to maintaining male circumcision coverage in the specified counties in western Kenya.

1. **Payment**

No payment or reimbursement will be provided to clients related to the evaluation, but transport facilitation costs will be covered as a standard element of VMMC service delivery in some settings. Transport facilitation for VMMC clients to and from VMMC service delivery points will not involve direct cash re-imbursements to clients. But where necessary, the implementing partner will provide appropriate transport means that ferries clients between designated community points and the VMMC service outlets. Such arrangement may involve the use of the implementing partner’s own vehicles or contracted local transport service providers. If model implementing partner(s) incur additional costs associated with cooperating with the evaluation, these costs may be reimbursed to the partner.

1. **Management**

## Oversight Plan

The primary evaluator representing Jhpiego Kenya will lead day-to-day oversight and management of the evaluation, and maintain coordination with MoH, the National VMMC Task Force, and county health leadership through quarterly updates in collaboration with CDC Kenya and the evaluating partner. County Health Teams will designate staff responsible for the county-level Ministry oversight; these staff will be involved in the oversight process for and will gradually take over management of the implementation, with support from CDC and implementing partners. Evaluating partner in-country staff will be responsible for data management and perform the quarterly model-level assessments that provide the primary data source for model course corrections and will provide a quarterly report to CDC which can be used in quarterly updates to NASCOP and the VMMC Task Force, as well as a summary of this report which will be provided to the county health teams.

## Stakeholder engagement

The National VMMC Task Force will engage other national-level arms of government to participate in meetings as appropriate. This will include engaging the Ministry of Education and Child’s Department in Nairobi prior to project implementation to obtain input on and support for school involvement in client recruiting and as a VMMC venue. The County Health Team will lead county-level engagement, which will include engaging similarly with the county-level educational hierarchy and Child’s Department (in Kisumu) prior to implementation.

## Personnel Qualifications

The evaluating partner personnel managing the project will be experienced providers of VMMC technical assistance who are familiar with the Kenya VMMC milieu.

## Staff Training

This investigation does not collect individual-level data or subject participants to interventions outside the national standard of care; staff will be those already employed in VMMC service delivery and their service delivery interactions with clients will not be affected by participation in the models. Therefore human subjects research protection training is not necessary. However, these staff will receive protocol-specific procedure training prior to model implementation. This will cover acceptable parameters for planning outreach events on schedules consistent with their assigned model, understanding standards by which model success will be evaluated, and maintaining appropriate cost records to support economic analysis.

## PI role

The PI or his designee will receive briefings quarterly and as needed on evaluation progress, and will participate in evaluation visits.

## Timeline

Assuming protocol approval by April 1

**Years 1-**3: Model implementation and evaluation

**Annually during this period:** Models will be reviewed for meeting criteria for discontinuation

**April-May 2017:** finalize model workplans, train staff, obtain agreement to participate from

leadership of involved facilities

**June 2017:** begin service delivery for all models

**June 2020:** present final results to stakeholders

**Years 4-5:** Transition of continuing models to MoH management and financing

**Every six months during this period:** Phone call or meeting between responsible CDC and

county MoH staff on transition progress

## Conflict of Interest

Investigators report no conflicts of interest with respect to the results or implementation of this protocol.

## Budget

If no additional funding is received for this activity, the planned budget is $250,000; $100,000 to be spent the first year, and $75,000 to be spent each of two following years.

1. PEPFAR 2015 technical considerations. [↑](#endnote-ref-1)
2. PEPFAR Sustainability Indices and Dashboards. President’s Emergency Plan for AIDS Relief, 2016. Available online at https://www.pepfar.gov/countries/cop/c71524.htm [↑](#endnote-ref-2)
3. Checklist for transition planning of national HIV responses. World Bank, 2016. Available online at http://documents.worldbank.org/curated/en/645871473879098475/pdf/108266-NEWS-WBChecklistforTransitionPlanning-PUBLIC.pdf [↑](#endnote-ref-3)
4. Oberth G, Whiteside A. What does sustainability mean in the HIV and AIDS response? Afr J AIDS Res. 2016;15(1):35-43. doi: 10.2989/16085906.2016.1138976. Epub 2016 Jan 20. [↑](#endnote-ref-4)
