# Supplementary material for: Making voluntary medical male circumcision services sustainable: Findings from Kenya’s pilot models, baseline and year 1
Source: PLoS One. 2021 Jun 11;16(6):e0252725. doi: 10.1371/journal.pone.0252725 (PMC8195380; doi:10.1371/journal.pone.0252725)
Supplement: S2 Appendix — (DOCX) [file pone.0252725.s002.docx]

**Project Title:** Identifying Sustainable Service Delivery Models to Maintain Medical Male Circumcision Coverage in Western Kenya

**Principal Investigator:** Stephanie Davis

**TOOL 4: KEY INFORMANT QUESTIONNAIRE – HIGH-LEVEL LEADERSHIP**

INSTRUCTIONS:

In the following questions, you are being asked to rate the VMMC sustainability project (services focused on 10-14-year-old boys to maintain high coverage) across various factors that affect sustainability. Please answer all questions about the project as a whole if you are on the national or county level, or about the model being used in your area if you are on the site level. If you feel you are not able to answer a question, please mark ‘N/A’ (not able to answer).

**This data is being collected to help the Ministry of Health determine which model(s) of VMMC service delivery are suitable for long-term use to maintain high VMMC coverage for HIV prevention.** **It is part of an evaluation of several models of delivery of sustainable VMMC services to determine which are successful in maintaining high coverage, low costs, complete ownership and leadership by the Ministry of Health, and other elements of sustainability. Each model attempts to deliver VMMC services to 10-14-year-old males in a way that is appropriate for its geographic area, and is evaluated over 3-5 years to determine whether it is successful and acceptable to those affected by it. Each model is expected to be successful in the area where it is used. The Ministry will be kept updated regularly about model performance, and the results will be published or put in a public report.**  Your name is not being recorded and will not be used. Filling out this form is voluntary and there are no consequences to you if you decline.

**If you have any questions or concerns about this evaluation, you can contact any of the below persons:**

**Principal Investigator Local Co-Investigator**

**Dr. Stephanie Davis Dr. Nandi Owuor**

[**smdavis@cdc.gov**](mailto:smdavis@cdc.gov) **nandi.owuor@jhpiego.org**

**1600 Clifton Rd. NE, MS E-04 Jhpiego Kenya Office, PO Box 66119-00800**

**Atlanta, GA 30033 +254722628770 or +254732134000**

**+1-404-718-4776**

**Secretariat, Masego University Ethics Review Committee**

[**muerc-secretariate@maseno.ac.ke**](mailto:muerc-secretariate@maseno.ac.ke)

**Directorate of Research, Publications and Innovations (DRPI)**

**Maseno University Main Campus**

**Along Kisumu-Busia Road**

**P. O. Box, Private Bag**

**Maseno, Kenya.**

**+ 254 57 351 622 EXT. 3050**

| **No.** | **CATEGORIES** | |
| --- | --- | --- |
|  | DATE (DD/MM/YY)  REGION  LOCATION (COMMUNITY/TOWN)  DIRECTORATE/DIVISION/DEPARTMENT/UNIT  TITLE  If you are on the site level : model being used in your area | _____________________________  _________________________________________________  _________________________________________________  ________________________________________________  _________________________________________________  __________________________________________________ |

**GOVERNANCE, LEADERSHIP AND ACCOUNTABILITY**

1. **PLANNING AND COORDINATION**
2. VMMC management roles and responsibilities between the county health team and all implementing partners are clearly defined.

Strongly disagree

Disagree

Neither agree nor disagree

Agree

Strongly agree

N/A

1. The county health team is committed to maintaining 80% VMMC coverage in the site’s catchment area

To very little or no extent

To a small extent

To some extent

To a great extent

To a very great extent

N/A

1. The county health team has translated national VMMC policies/strategies into county level VMMC strategic plans and response activities.

To very little or no extent

To a small extent

To some extent

To a great extent

To a very great extent

N/A

1. The county health team uses data to measure the effectiveness of the VMMC project in delivering needed VMMC services in the right locations.

Never

Rarely

Sometimes

Often

Always

N/A

1. Current and future staffing needs are based on the VMMC program goals and targets.

Never

Rarely

Sometimes

Often

Always

N/A

1. The county health team develops budgets that allocate resources to high need VMMC service delivery locations.

Never

Rarely

Sometimes

Often

Always

N/A

1. The county health team responsible for supervising, monitoring and supporting the VMMC sustainability project are qualified and competent to do so.

Strongly disagree

Disagree

Neither agree nor disagree

Agree

Strongly agree

N/A

1. The county health team supporting the VMMC sustainability project have enough time among their other responsibilities to dedicate to the project.

Strongly disagree

Disagree

Neither agree nor disagree

Agree

Strongly agree

N/A

1. The county health team supporting the VMMC sustainability project provides the necessary leadership and interactions required for successful project implementation.

Strongly disagree

Disagree

Neither agree nor disagree

Agree

Strongly agree

N/A

1. The county health team actively leads a mechanism or process (i.e., committee, working group, etc.) that routinely convenes stakeholders for VMMC planning and coordination purposes.

Never

Rarely

Sometimes

Often

Always

N/A

1. VMMC activities implemented by various stakeholders are effectively coordinated by the county health team.

Strongly disagree

Disagree

Neither agree nor disagree

Agree

Strongly agree

1. N/A
2. Communication between the VMMC sustainability project team and the county health team is effective.

Strongly disagree

Disagree

Neither agree nor disagree

Agree

Strongly agree

N/A

1. The goal of the VMMC sustainability project (maintaining 80% coverage) is well understood by all stakeholders.

Strongly disagree

Disagree

Neither agree nor disagree

Agree

Strongly agree

N/A

1. The VMMC sustainability project activities are harmonized with county strategies

To very little or no extent

To a small extent

To some extent

To a great extent

To a very great extent

N/A

1. The VMMC sustainability project has an effective strategy for handling increases in demand for VMMC services.

To very little or no extent

To a small extent

To some extent

To a great extent

To a very great extent

N/A

1. The VMMC sustainability project has an effective demand creation strategy for meeting performance targets.

To very little or no extent

To a small extent

To some extent

To a great extent

To a very great extent

N/A

1. **CIVIL SOCIETY ENGAGEMENT**
2. Diverse community groups are committed to the success of the VMMC sustainability project.

To very little or no extent

To a small extent

To some extent

To a great extent

To a very great extent

N/A

1. The county health team communicates the need for the program and provides important information about the VMMC program to community leaders

Never

Rarely

Sometimes

Often

Always

N/A

1. The county health team engages with civil society in VMMC program planning and client recruitment for VMMC.

Never

Rarely

Sometimes

Often

Always

N/A

1. The county health team engages with civil society in program evaluation and getting feedback from VMMC clients.

Never

Rarely

Sometimes

Often

Always

N/A

1. **TRANSPARENCY**
2. The VMMC sustainability project team makes VMMC expenditure summary reports available to stakeholders and the general public regularly.

Never

Rarely

Sometimes

Often

Always

N/A

1. VMMC program achievements are shared with stakeholders and the general public regularly.

Never

Rarely

Sometimes

Often

Always

N/A

1. There are clear and transparent processes for the selection of implementing partners for the VMMC sustainability project.

Strongly disagree

Disagree

Neither agree nor disagree

Agree

Strongly agree

N/A

1. There are clear and transparent processes for the hiring of staff for the VMMC sustainability project.

Strongly disagree

Disagree

Neither agree nor disagree

Agree

Strongly agree

N/A

OTHER

1. In your opinion, what are some of the major strengths of this model?
2. In your opinion, what are some of the major weakness of this model?
