# Supplementary material for: Making voluntary medical male circumcision services sustainable: Findings from Kenya’s pilot models, baseline and year 1
Source: PLoS One. 2021 Jun 11;16(6):e0252725. doi: 10.1371/journal.pone.0252725 (PMC8195380; doi:10.1371/journal.pone.0252725)
Supplement: S3 Appendix — (DOCX) [file pone.0252725.s003.docx]

**Project Title:** Identifying Sustainable Service Delivery Models to Maintain Medical Male Circumcision Coverage in Western Kenya

**Principal Investigator:** Dr. Stephanie Davis

**TOOL 5: KEY INFORMANT QUESTIONNAIRE – PROJECT AND SITE LEVEL**

INSTRUCTIONS:

In the following questions, you are being asked to rate the VMMC sustainability project across various factors that affect sustainability. Please answer all questions. If you feel you are not able to answer a question, please mark ‘NA’ (not able to answer).

This data is being collected to help the Ministry of Health determine which model(s) of VMMC service delivery are suitable for long-term use to maintain high VMMC coverage for HIV prevention. **This data is being collected to help the Ministry of Health determine which model(s) of VMMC service delivery are suitable for long-term use to maintain high VMMC coverage for HIV prevention.** **It is part of an evaluation of several models of delivery of sustainable VMMC services to determine which are successful in maintaining high coverage, low costs, complete ownership and leadership by the Ministry of Health, and other elements of sustainability. Each model attempts to deliver VMMC services to 10-14-year-old males in a way that is appropriate for its geographic area, and is evaluated over 3-5 years to determine whether it is successful and acceptable to those affected by it. Each model is expected to be successful in the area where it is used. The Ministry will be kept updated regularly about model performance, and the results will be published or put in a public report. Y**our name is not being recorded and will not be used. Filling out this form is voluntary and there are no consequences to you if you decline.

**If you have any questions or concerns about this evaluation, you can contact any of the below persons:**

**Principal Investigator Local Co-Investigator**

**Dr. Stephanie Davis Dr. Nandi Owuor**

[**smdavis@cdc.gov**](mailto:smdavis@cdc.gov) **nandi.owuor@jhpiego.org**

**1600 Clifton Rd. NE, MS E-04 Jhpiego Kenya Office, PO Box 66119-00800**

**Atlanta, GA 30033 +254722628770 or +254732134000**

**+1-404-718-4776**

**Secretariat, Masego University Ethics Review Committee**

[**muerc-secretariate@maseno.ac.ke**](mailto:muerc-secretariate@maseno.ac.ke)

**Directorate of Research, Publications and Innovations (DRPI)**

**Maseno University Main Campus**

**Along Kisumu-Busia Road**

**P. O. Box, Private Bag**

**Maseno, Kenya.**

**+ 254 57 351 622 EXT. 3050**

| **No.** | **CATEGORIES** | |
| --- | --- | --- |
|  | DATE  REGION  LOCATION (COMMUNITY/TOWN)  DIRECTORATE/DIVISION/DEPARTMENT/UNIT  TITLE | _____________________________  _________________________________________________  _________________________________________________  ________________________________________________  _________________________________________________ |

**GOVERNANCE, LEADERSHIP AND ACCOUNTABILITY**

1. **PLANNING AND COORDINATION**
2. VMMC management roles and responsibilities between the county health team and all implementing partners are clearly defined.

Strongly disagree

Disagree

Neither agree nor disagree

Agree

Strongly agree

N/A

1. The county health team is committed to maintaining 80% VMMC coverage in the site’s catchment area

To very little or no extent

To a small extent

To some extent

To a great extent

To a very great extent

N/A

1. The county health team has translated national VMMC policies/strategies into county level VMMC strategic plans and response activities.

To very little or no extent

To a small extent

To some extent

To a great extent

To a very great extent

N/A

1. The county health team uses data to measure the effectiveness of the VMMC project in delivering needed VMMC services in the right locations.

Never

Rarely

Sometimes

Often

Always

N/A

1. Current and future staffing needs are based on the VMMC program goals and targets.

Never

Rarely

Sometimes

Often

Always

N/A

1. The county health team develops budgets that allocate resources to high need VMMC service delivery locations.

Never

Rarely

Sometimes

Often

Always

N/A

1. The county health team responsible for supervising, monitoring and supporting the VMMC sustainability project are qualified and competent to do so.

Strongly disagree

Disagree

Neither agree nor disagree

Agree

Strongly agree

N/A

1. The county health team supporting the VMMC sustainability project have enough time among their other responsibilities to dedicate to the project.

Strongly disagree

Disagree

Neither agree nor disagree

Agree

Strongly agree

N/A

1. The county health team supporting the VMMC sustainability project provides the necessary leadership and interactions required for successful project implementation.

Strongly disagree

Disagree

Neither agree nor disagree

Agree

Strongly agree

N/A

1. The county health team actively leads a mechanism or process (i.e., committee, working group, etc.) that routinely convenes stakeholders for VMMC planning and coordination purposes.

Never

Rarely

Sometimes

Often

Always

N/A

1. VMMC activities implemented by various stakeholders are effectively coordinated by the county health team.

Strongly disagree

Disagree

Neither agree nor disagree

Agree

Strongly agree

N/A

1. Communication between the VMMC sustainability project team and the county health team is effective.

Strongly disagree

Disagree

Neither agree nor disagree

Agree

Strongly agree

N/A

1. The goal of the VMMC sustainability project (maintaining 80% coverage) is well-understood by all stakeholders.

Strongly disagree

Disagree

Neither agree nor disagree

Agree

Strongly agree

N/A

1. The VMMC sustainability project activities are harmonized with county strategies

To very little or no extent

To a small extent

To some extent

To a great extent

To a very great extent

N/A

1. The VMMC sustainability project has an effective strategy for handling increases in demand for VMMC services.

To very little or no extent

To a small extent

To some extent

To a great extent

To a very great extent

N/A

1. The VMMC sustainability project has an effective demand creation strategy for meeting performance targets.

To very little or no extent

To a small extent

To some extent

To a great extent

To a very great extent

N/A

1. **CIVIL SOCIETY ENGAGEMENT**
2. Diverse community groups are committed to the success of the VMMC sustainability project.

To very little or no extent

To a small extent

To some extent

To a great extent

To a very great extent

N/A

1. The county health team communicates the need for the program and provides important information about the VMMC program to community leaders

Never

Rarely

Sometimes

Often

Always

N/A

1. The county health team engages with civil society in program planning and client recruitment for VMMC.

Never

Rarely

Sometimes

Often

Always

N/A

1. The county health team engages with civil society in program evaluation and getting feedback from VMMC clients.

Never

Rarely

Sometimes

Often

Always

N/A

1. **TRANSPARENCY**
2. The VMMC sustainability project team makes VMMC expenditure summary reports available to stakeholders and the general public regularly.

Never

Rarely

Sometimes

Often

Always

N/A

1. VMMC program achievements are shared with stakeholders and the general public regularly.

Never

Rarely

Sometimes

Often

Always

N/A

1. There are clear and transparent processes for the selection of implementing partners for the VMMC sustainability project.

Strongly disagree

Disagree

Neither agree nor disagree

Agree

Strongly agree

N/A

1. There are clear and transparent processes for the hiring of staff for the VMMC sustainability project.

Strongly disagree

Disagree

Neither agree nor disagree

Agree

Strongly agree

N/A

**NATIONAL HEALTH SYSTEM AND SERVICE DELIVERY**

1. **DOMESTIC SERVICE DELIVERY**
2. The county health team executes their VMMC responsibilities without the need for external technical assistance.

To very little or no extent

To a small extent

To some extent

To a great extent

To a very great extent

N/A

1. If VMMC technical assistance is needed, these needs have been identified.

Strongly disagree

Disagree

Neither agree nor disagree

Agree

Strongly agree

N/A

1. If there are any VMMC technical assistance needs identified, there is a process in place for addressing these.

Strongly disagree

Disagree

Neither agree nor disagree

Agree

Strongly agree

N/A

1. The VMMC sustainability project team executes their responsibilities without the need for external technical assistance.

Strongly disagree

Disagree

Neither agree nor disagree

Agree

Strongly agree

N/A

1. If technical assistance is needed by VMMC sustainability project team, these needs have been identified.

Strongly disagree

Disagree

Neither agree nor disagree

Agree

Strongly agree

N/A

1. If there are any technical assistance needs identified by the VMMC sustainability project team, there is a process in place for addressing these.

Strongly disagree

Disagree

Neither agree nor disagree

Agree

Strongly agree

N/A

1. VMMC services are delivered as designed by the VMMC sustainability Project.

To very little or no extent

To a small extent

To some extent

To a great extent

To a very great extent

N/A

1. VMMC services are easily accessible to interested clients.

Strongly disagree

Disagree

Neither agree nor disagree

Agree

Strongly agree

N/A

1. VMMC services do not interfere with other health services provided at the site.

Strongly disagree

Disagree

Neither agree nor disagree

Agree

Strongly agree

N/A

1. Recruitment of clients for VMMC does not interfere with any services provided at the recruitment venues.

Strongly disagree

Disagree

Neither agree nor disagree

Agree

Strongly agree

N/A

1. VMMC services are offered in a manner acceptable to the community.

Strongly disagree

Disagree

Neither agree nor disagree

Agree

Strongly agree

N/A

1. **HUMAN RESOURCES FOR HEALTH**
2. The staff assigned to the VMMC sustainability project are qualified to meet the necessary targets to maintain 80% coverage of the target-aged population in the catchment area.

Strongly disagree

Disagree

Neither agree nor disagree

Agree

Strongly agree

N/A

1. All VMMC **field staff** are trained and certified to perform their duties as assigned.

Strongly disagree

Disagree

Neither agree nor disagree

Agree

Strongly agree

N/A

1. The staff assigned to the VMMC sustainability project are sufficient to meet the necessary targets to maintain 80% coverage of the target-aged population in the catchment area.

Strongly disagree

Disagree

Neither agree nor disagree

Agree

Strongly agree

N/A

1. VMMC is offered regularly as planned.

Never

Rarely

Sometimes

Often

Always

N/A

1. There are waiting lists for VMMC services.

Never

Rarely

Sometimes

Often

Always

N/A

1. Routine VMMC service delivery is task-shifted to the lowest permitted cadre

To very little or no extent

To a small extent

To some extent

To a great extent

To a very great extent

N/A

1. Staff from this cadre routinely perform all VMMC surgical/procedural steps without on-site supervision from a higher cadre.

Never

Rarely

Sometimes

Often

Always

N/A

1. **SUPPLY CHAIN**
2. There are established processes for materials procurement, distribution and reprocessing for the VMMC program.

Strongly disagree

Disagree

Neither agree nor disagree

Agree

Strongly agree

N/A

1. Supply processes work well enough to provide everything needed to ensure client safety and achieve VMMC targets.

Never

Rarely

Sometimes

Often

Always

N/A

1. In instances where supply processes interfere with VMMC safety and target achievement, specific deficiencies are addressed.

Never

Rarely

Sometimes

Often

Always

N/A

1. In the past quarter, supply processes worked well enough to ensure VMMC service was delivered as planned.

Never

Rarely

Sometimes

Often

Always

N/A

1. **QUALITY MANAGEMENT**
2. Staff have the training and capacity to apply VMMC quality improvement methods.

Strongly disagree

Disagree

Neither agree nor disagree

Agree

Strongly agree

N/A

1. Performance against VMMC targets is reviewed at least quarterly with the service delivery team.

Never

Rarely

Sometimes

Often

Always

N/A

1. Performance against VMMC quality standards is reviewed at least quarterly with the service delivery team.

Never

Rarely

Sometimes

Often

Always

N/A

1. The VMMC sustainability team reviews has a process for reviewing adverse events.

Strongly disagree

Disagree

Neither agree nor disagree

Agree

Strongly agree

N/A

1. VMMC performance data is used for improvements and decision-making

To very little or no extent

To a small extent

To some extent

To a great extent

To a very great extent

N/A

1. The county health team reviews unit costs for the VMMC sustainability project when they become available.

Strongly disagree

Disagree

Neither agree nor disagree

Agree

Strongly agree

N/A

1. The VMMC sustainability team uses unit costs for planning services

Strongly disagree

Disagree

Neither agree nor disagree

Agree

Strongly agree

N/A

**STRATEGIC INVESTMENTS, EFFICIENCY AND SUSTAINABLE FINANCING**

1. **DOMESTIC RESOURCE MOBILIZATION**
2. The VMMC sustainability team is aware of and fully engaged in planning the transition to county-based funding for VMMC.

To very little or no extent

To a small extent

To some extent

To a great extent

To a very great extent

N/A

1. The VMMC sustainability team needs technical assistance in program finance planning

To very little or no extent

To a small extent

To some extent

To a great extent

To a very great extent

N/A

1. If there are any VMMC technical assistance needs identified, there is a process in place for addressing these.

Strongly disagree

Disagree

Neither agree nor disagree

Agree

Strongly agree

N/A

1. Domestic funding sources for VMMC program support have been identified.

To very little or no extent

To a small extent

To some extent

To a great extent

To a very great extent

N/A

1. **TECHNICAL AND ALLOCATIVE EFFICIENCES**
2. The sustainability project team reviews VMMC program costs annually.

Never

Rarely

Sometimes

Often

Always

N/A

1. The VMMC sustainability project team identifies opportunities for improving cost efficiency without sacrificing safety.

Never

Rarely

Sometimes

Often

Always

N/A

1. The VMMC sustainability project team uses cost saving strategies without sacrificing safety

Never

Rarely

Sometimes

Often

Always

N/A

**STRATEGIC INFORMATION**

1. **PERFORMANCE DATA**
2. The VMMC sustainability project team complies with reporting expectations and procedures in a timely manner.

To very little or no extent

To a small extent

To some extent

To a great extent

To a very great extent

N/A

1. Site-level VMMC performance data is submitted to the county health team through the national reporting system.

Never

Rarely

Sometimes

Often

Always

N/A

**OTHER**

1. In your opinion, what are some of the major strengths of this model?
2. In your opinion, what are some of the major weakness of this model?
