# Supplementary material for: Making voluntary medical male circumcision services sustainable: Findings from Kenya’s pilot models, baseline and year 1
Source: PLoS One. 2021 Jun 11;16(6):e0252725. doi: 10.1371/journal.pone.0252725 (PMC8195380; doi:10.1371/journal.pone.0252725)
Supplement: S4 Appendix — (DOCX) [file pone.0252725.s004.docx]

**Project Title:** Identifying Sustainable Service Delivery Models to Maintain Medical Male Circumcision Coverage in Western Kenya

**Principal Investigator:** Dr. Stephanie Davis

**TOOL 6: KEY INFORMANT QUESTIONNAIRE – COMMUNITY LEVEL**

INSTRUCTIONS:

Greetings. The Ministry of Health has been offering male circumcision services at health facilities or through mobile services in your community. We are asking you to tell us what you think about the male circumcision program and the services provided. Your answers will help the Ministry to continue offering these services to more boys. Please answer all questions. If you feel you are not able to answer a question, please mark ‘N/A’ (not able to answer).

**This data is being collected to help the Ministry of Health determine which model(s) of VMMC service delivery are suitable for long-term use to maintain high VMMC coverage for HIV prevention.** **It is part of an evaluation of several models of delivery of sustainable VMMC services to determine which are successful in maintaining high coverage, low costs, complete ownership and leadership by the Ministry of Health, and other elements of sustainability. Each model attempts to deliver VMMC services to 10-14-year-old males in a way that is appropriate for its geographic area, and is evaluated over 3-5 years to determine whether it is successful and acceptable to those affected by it. Each model is expected to be successful in the area where it is used. The Ministry will be kept updated regularly about model performance, and the results will be published or put in a public report.**  Your name is not being recorded and will not be used. Filling out this form is voluntary and there are no consequences to you if you decline.

**If you have any questions or concerns about this evaluation, you can contact any of the below persons:**

**Principal Investigator Local Co-Investigator**

**Dr. Stephanie Davis Dr. Nandi Owuor**

[**smdavis@cdc.gov**](mailto:smdavis@cdc.gov) **nandi.owuor@jhpiego.org**

**1600 Clifton Rd. NE, MS E-04 Jhpiego Kenya Office, PO Box 66119-00800**

**Atlanta, GA 30033 +254722628770 or +254732134000**

**+1-404-718-4776**

**Secretariat, Masego University Ethics Review Committee**

[**muerc-secretariate@maseno.ac.ke**](mailto:muerc-secretariate@maseno.ac.ke)

**Directorate of Research, Publications and Innovations (DRPI)**

**Maseno University Main Campus**

**Along Kisumu-Busia Road**

**P. O. Box, Private Bag**

**Maseno, Kenya.**

**+ 254 57 351 622 EXT. 3050**

| **CATEGORIES** | | |
| --- | --- | --- |
|  | DATE  REGION  COMMUNITY/TOWN/ WARD | _____________________________  _________________________________________________  _________________________________________________ |
| **DEMOGRAPHICS** | | |
|  | Sex of respondent | Male  Female |
|  | Age at last birthday |  |
|  | Are you a: | Ward representative  Religious leader  Community leader  School teacher  Student  Other: |

1. **COMMUNITY ENGAGEMENT AND PARTICIPATION**
2. How often do you participate in meetings with other community leaders or community members to talk about the male circumcision services in your community?

Never

Rarely

Sometimes

Often

Always

N/A

1. How often do you participate in meetings with the Ministry of Health to plan the male circumcision services being offered in your community?

Never

Rarely

Sometimes

Often

Always

N/A

1. In general, community representatives have a lot of influence in the way male circumcision services are provided in this community.

Strongly disagree

Disagree

Neither agree nor disagree

Agree

Strongly agree

N/A

1. How often do you participate in meetings with the Ministry of Health to provide feedback about male circumcision services being offered in your community?

Never

Rarely

Sometimes

Often

Always

N/A

1. In general, feedback provided by community representatives is used to improve the way male circumcision services are provided in this community.

Strongly disagree

Disagree

Neither agree nor disagree

Agree

Strongly agree

N/A

1. The Ministry of Health shares important information about the male circumcision program in your community.

Never

Rarely

Sometimes

Often

Always

N/A

1. The information you receive about the male circumcision program helps you to know more about the services offered in my community

Strongly disagree

Disagree

Neither agree nor disagree

Agree

Strongly agree

N/A

1. The information you receive about the male circumcision program helps you to know how well the program is doing.

Strongly disagree

Disagree

Neither agree nor disagree

Agree

Strongly agree

N/A

1. How often do you participate in activities to recruit boys for male circumcision in your community?

Never

Rarely

Sometimes

Often

Always

N/A

1. In general, community representatives are willing to support the male circumcision program in this community.

Strongly disagree

Disagree

Neither agree nor disagree

Agree

Strongly agree

N/A

**II. SERVICE DELIVERY**

1. Most people know about the male circumcision services offered in the community.

Strongly disagree

Disagree

Neither agree nor disagree

Agree

Strongly agree

N/A

1. If a boy between 10-14 years old wants to get circumcised, can he easily get the service?

Never

Rarely

Sometimes

Often

Always

N/A

1. Would you say that most boys who go to get circumcised, get services on the same day?

Never

Rarely

Sometimes

Often

Always

N/A

1. How do you think people feel about the way male circumcision services are provided in your community?

Strongly disagree

Disagree

Neither agree nor disagree

Agree

Strongly agree

N/A

1. Male circumcision activities conducted in the community are planned and coordinated such that they do not disrupt other services, including the school timetable.

Strongly disagree

Disagree

Neither agree nor disagree

Agree

Strongly agree

N/A

**OTHER**

1. In your opinion, what are some of the best things about the way male circumcision services are provided in your community?
2. In your opinion, what are some of the worst things about the way male circumcision services are provided in your community?

THANK YOU FOR YOUR PARTICIPATION
