# Supplementary material for: Making voluntary medical male circumcision services sustainable: Findings from Kenya’s pilot models, baseline and year 1
Source: PLoS One. 2021 Jun 11;16(6):e0252725. doi: 10.1371/journal.pone.0252725 (PMC8195380; doi:10.1371/journal.pone.0252725)
Supplement: S5 Appendix — (DOCX) [file pone.0252725.s005.docx]

**Appendix A:** **Annual Evaluation Metrics for VMMC Sustainability Pilot, Kenya**

**Purpose:** This evaluation will compare models at the same point in time to determine which are most viable, and also to track each model’s progress over time. It is designed to contain both process and outcome indicators of sustainability. Both are expected to be lacking initially and to develop over time in the model(s) that emerge as viable. Not all of these indicators are equally under the control of the project implementation staff, but all should help to provide an overall picture of the sustainability of the piloted models. Source documents used in developing this metric include the PEPFAR Sustainability Index Dashboard (SID) 2.0, the World Bank Checklist for Transition Planning of National HIV Responses, and Obert and Whitside’s 2016 AJAR article “What does sustainability mean in the HIV and AIDS response?”.

**Directions:**  Please answer all questions, using key informant interviews, site visits and record and document review as appropriate. In key informant interviews, where possible please ask open-ended questions about processes and outcomes rather than asking leading questions about whether indicators are being met, to elicit the most accurate information. For any “no” answer, please investigate and provide a brief description of plans for and barriers to achieving the indicator, including whether barriers appear to be related or unrelated to the specific service delivery model involved. After completing this assessment, please review findings and recommendations with the service delivery team and the Ministry of Health point of contact.

**Metrics for performance comparison between models:**

| Strategic Investments, Efficiency, and Sustainable Financing | Technical and Allocative Efficiencies | What is the most recent unit cost per VMMC in the target age range?  Timeframe covered by data: ____________________ | (#) | Data Source |
| --- | --- | --- | --- | --- |
| Strategic Investments, Efficiency, and Sustainable Financing | Technical and Allocative Efficiencies | What is the breakdown of costs between cost categories? | (%’s) |  |
| National Health System and Service Delivery | Domestic Service Delivery | What number and percent of the quarterly target of VMMCs in the target age group was achieved in the last quarter? | (#) |  |
| National Health System and Service Delivery | Domestic Service Delivery | What is the AE rate (moderate and severe) in a random sample of 200 client charts? | (#) |  |
| National Health System and Service Delivery | Quality Mgmt. | The model’s service delivery site performed satisfactorily (no findings that would require site shutdown) on an External Quality Assurance assessment performed by the evaluating partner within the past year. | Y/N |  |

**Metrics for assessment of other sustainability aspects achieved for each model:**

| **Domain** | **Area** | **Questions** | **Answer** | **Data Source** |
| --- | --- | --- | --- | --- |
| Governance, Leadership, and Accountability | Planning and Coordination | There is documentation of the division of management roles and responsibilities between the MoH and any implementing partner, and both parties have this documentation. | Y/N |  |
| Governance, Leadership, and Accountability | Planning and Coordination | County health leadership is committed to maintaining countywide VMMC saturation. | Y/N |  |
| Governance, Leadership, and Accountability | Planning and Coordination | The county MoH has a currently active written strategy that includes maintaining the sustainability model services. | Y/N |  |
| Governance, Leadership, and Accountability | Planning and Coordination | The strategy includes explicit specifications for the activities and deliverables expected from the model (e.g. catchment area, demographically accurate targets to maintain saturation, engagement with venues, etc.) | Y/N |  |
| Governance, Leadership, and Accountability | Planning and Coordination | The county MoH has a written numerical annual VMMC target, based on demographic data, for maintaining long-term countywide VMMC saturation. | Y/N |  |
| Governance, Leadership, and Accountability | Planning and Coordination | The county MoH and service delivery team have agreed on written numerical annual targets, based on demographic data, for maintaining saturation in the team’s catchment area. |  |  |
| Governance, Leadership, and Accountability | Planning and Coordination | The MoH staff responsible for supervising, monitoring and supporting the models are qualified and competent to do so, and have sufficient bandwidth among their other responsibilities. | Y/N |  |
| Governance, Leadership, and Accountability | Planning and Coordination | The MoH parties responsible for supervising, monitoring and supporting the models are actively engaged in providing the necessary leadership and interactions to discharge these responsibilities. | Y/N |  |
| Governance, Leadership, and Accountability | Planning and Coordination | Relationships between the implementing partner and the supervising MoH staff are characterized by good communication, trust, and the shared goal of maintaining VMMC saturation. | Y/N |  |
| Governance, Leadership, and Accountability | Planning and Coordination | The model’s service delivery team has a schedule of service, demand creation and engagement/coordination activities, planned a year in advance, which is harmonized with the county strategy, and which if implemented correctly could meet targets. | Y/N |  |
| Governance, Leadership, and Accountability | Planning and Coordination | If applicable for this model: The model’s service delivery team has a signed Memorandum of Understanding or equivalent document with each venue where it will perform circumcision (schools, churches, businesses frequented by potential clients, etc.) that details terms of engagement, except for public-space venues where no party exists to sign such an MOU. | Y/N/NA |  |
| Governance, Leadership, and Accountability | Planning and Coordination | The model’s service delivery team’s relationships with venues consistently result in the necessary access to the venues to meet targets. | Y/N/NA |  |
| Governance, Leadership, and Accountability | Planning and Coordination | The model has a feasible strategy in place for handling surges in demand (more staff can be temporarily assigned, etc.) | Y/N |  |
| Governance, Leadership, and Accountability | Planning and Coordination | The model includes a demand creation strategy adequate for meeting performance targets. | Y/N |  |
| Governance, Leadership, and Accountability | Non-discrimin-ation | No MoH strategy document discriminates against MSM, HIV-positive males, other males who are outside the typical VMMC target population but within the target age range, or males who decline HIV testing, in the provision of VMMC services. | Y/N |  |
| Governance, Leadership, and Accountability | Non-discrimin-ation | The model’s service delivery team does not discriminate against MSM, HIV-positive males, other males who are outside the typical VMMC target population but within the target age range, or males who decline HIV testing, in the provision of VMMC services. |  |  |
| National Health System and Service Delivery | Domestic Service Delivery | If there are technical assistance needs on the part of the county MoH around supervising, monitoring and supporting the active models, these have been identified. | Y/N/NA |  |
| National Health System and Service Delivery | Domestic Service Delivery | Services are being delivered in a manner consistent with the selected model as described in the protocol. | Y/N |  |
| National Health System and Service Delivery | Domestic Service Delivery | If there are technical assistance needs on the part of the county MoH around supervising, monitoring or supporting the active models, there is a process in place for addressing these. | Y/N/NA |  |
| National Health System and Service Delivery | Domestic Service Delivery | The MoH parties responsible for supervising, monitoring and supporting the active models execute their responsibilities without the need for external technical assistance. | Y/N/NA |  |
| National Health System and Service Delivery | Domestic Service Delivery | If there are technical assistance needs on the part of the model’s service delivery team in the areas of planning, implementation, self-monitoring or continuous quality assurance, these have been identified. | Y/N/NA |  |
| National Health System and Service Delivery | Domestic Service Delivery | If there are technical assistance needs on the part of the model’s service delivery team in the areas of planning, implementation, self-monitoring or continuous quality assurance, there is a process in place for addressing these. | Y/N/NA |  |
| National Health System and Service Delivery | Domestic Service Delivery | The model’s service delivery team executes its planning responsibilities without the need for external technical assistance. | Y/N |  |
| National Health System and Service Delivery | Domestic Service Delivery | The model’s service delivery team executes its implementation responsibilities without the need for external technical assistance. | Y/N |  |
| National Health System and Service Delivery | Domestic Service Delivery | The model’s service delivery team executes its self-monitoring responsibilities without the need for external technical assistance. | Y/N |  |
| National Health System and Service Delivery | Domestic Service Delivery | The model’s service delivery team executes its continuous quality assurance responsibilities without the need for external technical assistance. | Y/N |  |
| National Health System and Service Delivery | Domestic Service Delivery | Interested clients are able to access the services easily. | Y/N |  |
| National Health System and Service Delivery | Domestic Service Delivery | Service provision does not interfere with other health services at the site. | Y/N/NA |  |
| National Health System and Service Delivery | Domestic Service Delivery | Service provision does not unduly disrupt the intended functions of the venues (e.g. schools) from which clients are recruited. | Y/N |  |
| National Health System and Service Delivery | Domestic Service Delivery | The way services are offered is acceptable to the community. | Y/N |  |
| National Health System and Service Delivery | Human Resources for Health | There are qualified and sufficient **field staff** assigned to the model to meet the necessary targets to maintain 80% coverage of the target-aged population in the catchment area. | Y/N |  |
| National Health System and Service Delivery | Human Resources for Health | There are qualified and sufficient **leadership staff** assigned to the model to meet the necessary targets to maintain 80% coverage of the target-aged population in the catchment area. | Y/N |  |
| National Health System and Service Delivery | Human Resources for Health | The county MoH has identified in writing the person in charge of supervising, monitoring and supporting the active models. If these responsibilities are shared among multiple people, their division is clearly documented. | Y/N |  |
| National Health System and Service Delivery | Human Resources for Health | The model’s routine service delivery is task-shifted to the lowest permitted cadre. | Y/N |  |
| National Health System and Service Delivery | Supply Chain | Processes for materials procurement, distribution and reprocessing are in place and documented. | Y/N |  |
| National Health System and Service Delivery | Supply Chain | If supply processes problems have interfered with safety or target achievement, the specific deficiencies have been identified. | Y/N |  |
| National Health System and Service Delivery | Supply Chain | If supply processes problems have interfered with safety or target achievement, the specific deficiencies are being addressed. | Y/N |  |
| National Health System and Service Delivery | Supply Chain | Processes for materials procurement, distribution and reprocessing have allowed the program to operate without service interruptions due to supply or commodity problems over the past quarter. | Y/N |  |
| National Health System and Service Delivery | Supply Chain | The costs of VMMC commodities compare are the same as or lower than international benchmark costs for similar commodities. | Y/N |  |
| National Health System and Service Delivery | Quality Mgmt. | The model team’s staff have the training and capacity to apply quality improvement methods. | Y/N |  |
| National Health System and Service Delivery | Quality Mgmt. | The model team has a functioning continuous quality improvement system in place, with documentation of a meeting within the past quarter. | Y/N |  |
| National Health System and Service Delivery | Quality Mgmt. | The MoH parties responsible for supervising, monitoring and supporting the active models review model performance against **targets** at least quarterly, and hold IP accountable. | Y/N |  |
| National Health System and Service Delivery | Quality Mgmt. | The MoH parties responsible for supervising, monitoring and supporting the active models review model performance against **quality standards** (through EQA and AE reporting) at least annually, and hold IP accountable. |  |  |
| National Health System and Service Delivery | Quality Mgmt. | The MoH parties responsible for supervising, monitoring and supporting the active models review model **unit costs** at least annually, and hold IP accountable. |  |  |
| National Health System and Service Delivery | Quality Mgmt. | The model’s service delivery team has an adverse event review process in place (can be part of its CQI activities). | Y/N |  |
| National Health System and Service Delivery | Quality Mgmt. | The model’s service delivery team reviews cost data and uses it in planning services at least annually. |  |  |
| Governance, Leadership, and Accountability | Civil Society Engagement | The model’s service delivery team and/or responsible MoH staff are actively engaged with civil society groups in the model catchment areas, e.g., seeking feedback on service performance and/or enlisting help in client recruitment and venue development. | Y/N |  |
| Governance, Leadership, and Accountability | Transparency | The MoH makes the model’s VMMC budget or expenditure analysis data publically available. | Y/N |  |
| Governance, Leadership, and Accountability | Transparency | The MoH makes VMMC achievement data, by age, publically available within 1 year. | Y/N |  |
| Governance, Leadership, and Accountability | Transparency | The MoH makes selection processes for any implementing partner, or staff hiring for the model, public in a timely way. | Y/N |  |
| Strategic Investments, Efficiency, and Sustainable Financing | Domestic Resource Mobilization | The key MoH staff in all areas crucial to the county’s health finance planning process (may include health, finance, county executive leadership, etc.) are aware of and engaged in planning the transition to county-based funding. |  |  |
| Strategic Investments, Efficiency, and Sustainable Financing | Domestic Resource Mobilization | Any technical assistance needs in the area of program finance planning have been identified. | Y/N |  |
| Strategic Investments, Efficiency, and Sustainable Financing | Domestic Resource Mobilization | A written plan is in place for meeting any technical assistance needs identified in program finance planning. | Y/N |  |
| Strategic Investments, Efficiency, and Sustainable Financing | Domestic Resource Mobilization | Any technical assistance needs that have been identified in program finance planning are being met. | Y/N |  |
| Strategic Investments, Efficiency, and Sustainable Financing | Domestic Resource Mobilization | The county MoH has a functional mechanism by which to pay for the execution of service delivery. | Y/N |  |
| Strategic Investments, Efficiency, and Sustainable Financing | Domestic Resource Mobilization | The county has identified and documented the funding sources it intends to use to provide its support to the VMMC program and amount from each (e.g. cost-sharing, annual health budget, etc.) |  |  |
| Strategic Investments, Efficiency, and Sustainable Financing | Domestic Resource Mobilization | The county MoH has a dedicated funding stream for implementation of the sustainability model(s). | Y/N |  |
| Strategic Investments, Efficiency, and Sustainable Financing | Domestic Resource Mobilization | If Y to the above, the county MoH has an explicit budget line item for implementation of the sustainability model(s). | Y/N |  |
| Strategic Investments, Efficiency, and Sustainable Financing | Domestic Resource Mobilization | What percent of the cost of program implementation (not external evaluation) has the MoH funded for the past quarter in each of the following major cost categories? (include any revenue from cost-sharing, and any in-kind contributions at their local cost)  Human resources for routine services  Human resources for technical assistance & quality assurance  Commodities/ consumables  Facility operation, transport, waste management  Demand creation | (#)  ______  ______  ______  ______  ______ |  |
| Strategic Investments, Efficiency, and Sustainable Financing | Domestic Resource Mobilization | What percent of county HIV resources are allocated to VMMC? |  |  |
| Strategic Investments, Efficiency, and Sustainable Financing | Domestic Resource Mobilization | The county has a regular auditing policy in place which includes VMMC expenditures. | Y/N |  |
| Strategic Investments, Efficiency, and Sustainable Financing | Domestic Resource Mobilization | Cost data is made available at least annually. | Y/N |  |
| Strategic Investments, Efficiency, and Sustainable Financing | Domestic Resource Mobilization | A written plan is in place documenting what level of funding responsibility to county government intends to take on year by year over the life of the project. | Y/N |  |
| Strategic Investments, Efficiency, and Sustainable Financing | Technical and Allocative Efficiencies | At least annually, the model’s service delivery team performs a thorough review of cost drivers and identifies opportunities for improving cost efficiency without sacrificing safety. | Y/N |  |
| Strategic Investments, Efficiency, and Sustainable Financing | Technical and Allocative Efficiencies | The model’s service delivery team is using reusable surgical tools, a proven cost minimization method, exclusively or almost exclusively in the sustainability models. | Y/N |  |
| Strategic Investments, Efficiency, and Sustainable Financing | Technical and Allocative Efficiencies | The model’s service delivery team has taken advantage of all other apparent cost-saving opportunities that do not compromise care or service reliability (e.g. free outreach housing through village counsels or at military bases, etc.) | Y/N |  |
| Strategic Investments, Efficiency, and Sustainable Financing | Technical and Allocative Efficiencies | Avoidable overheads (e.g. multiple layers of subcontractors) have been eliminated from service implementation. | Y/N |  |
| Strategic Investments, Efficiency, and Sustainable Financing | Technical and Allocative Efficiencies | Paid staff downtime is minimal. | Y/N |  |
| Strategic Information | Epidem-iological and Health Data | The model’s service delivery team maintains written information about the demographics and population of the catchment area, updated at least every three years. | Y/N |  |
| Strategic Information | Epidem-iological and Health Data | For models where this is applicable, the model’s service delivery team maintains venue mapping information, including ascertaining emergence of new venues at least every year. | Y/N |  |
| Strategic Information | Performance Data | Clear written reporting expectations and procedures are in place for the model’s service delivery team which include, at minimum, VMMCs performed in the target age group and AEs diagnosed (these procedures may be national-level). | Y/N |  |
| Strategic Information | Performance Data | The team complies with these procedures, in a timely manner and within any time frame the procedures stipulate. | Y/N |  |
| Strategic Information | Performance Data | Site-level record review and comparison with reported data demonstrates >95% reporting of required indicators. | Y/N |  |
| Strategic Information | Performance Data | Performance data is being reliably entered into the appropriate national reporting system. | Y/N |  |
| Strategic Information | Performance Data | The responsible county-level MoH staff reliably receive the data reported through the national reporting system (Situation Room?) | Y/N |  |
