# Supplementary material for: Making voluntary medical male circumcision services sustainable: Findings from Kenya’s pilot models, baseline and year 1
Source: PLoS One. 2021 Jun 11;16(6):e0252725. doi: 10.1371/journal.pone.0252725 (PMC8195380; doi:10.1371/journal.pone.0252725)
Supplement: S6 Appendix — (DOCX) [file pone.0252725.s006.docx]

**Appendix D: Key assumptions used in economic analysis**

Exhibit 1: Assumptions used in estimating baseline total and unit expenditures in Siaya County, and mapping to categories used in the main manuscript

| **General descriptive category** | **Mapping to subset of categories in main manuscript table 4, where relevant** | **Mapping to fine category(ies) in main manuscript figure 2** | **Description** | **Basis for allocation** | **Percentage** |
| --- | --- | --- | --- | --- | --- |
| Administrative costs (non-staff) | Facility operation, transport, waste management | Administrative cost | Includes all cost related to office running, which is shared amongst several programs. Includes transport for general oversight purposes but not program-specific/service delivery transport. | Allocation is based on proportion of staff dedicated to the VMMC program (33/161 = 19%), proportion of MCs to other programs, space utilization of staff providing VMMC services and other programs etc. | 19% of the total expenditure on administrative costs |
|  |  | Administrative supplies |  |  |  |
| Meetings and trainings | Human resources for technical assistance & quality assurance (IP/PEPFAR) | Meetings | Expenditure on meetings and trainings | All the expenditures attributed to VMMC program | 100% allocation to VMMC program |
|  |  | Training |  |  |  |
| Expenditure on medical equipment |  | Investment | All VMMC medical equipment were purchased by CHS | All Medical equipment dedicated to VMMC program. Cost of equipment (purchased or donated before baseline) amortized using useful life years, inflation rate and present value | 100% allocation to VMMC program. Estimated cost per annum was included in the baseline expenditure estimations |
| Medicines, medical supplies and consumables | Commodities/supplies | Commodities | This includes cost of medicines and medical supplies e.g. surgical packs, gloves etc. | These are direct program costs | 100% allocation to VMMC program |
| Other direct program costs | Facility operation, transport, waste management | Mobilization costs | These includes the cost incurred directly to support the mobilization of clients for VMMC services | These are direct program costs | 100% allocation to VMMC program |
|  |  | Other direct Costs | These include operational costs e.g. transport costs, communication costs | Proportion of staff dedicated to VMMC program. | 19% of expenditure on airtime |
|  |  | Airtime | The includes the airtime costs utilized by the VMMC program | These are direct program costs | 100% allocation to VMMC program |
|  |  | MV Maintenance | Includes cost of maintenance, fuel, and vehicle rentals. For Siaya, CHS purchased vehicles for use in the VMMC program as well as in other programs in the county | Ideally, allocation should be based on mileage of vehicle use in the VMMC program or fuel consumption. However, this information was not readily available | We used the proportion of VMMC sites among total program sites to estimate expenditure attributed to VMMC program. This was 6 of 29, or 55%. |
|  | Facility operation, transport, waste management | Travel |  |  |  |
|  | Human resources for technical assistance & quality assurance (IP/PEPFAR) | Support Supervision | These includes the costs related to supportive supervision. | This mainly joint support supportive supervision done to all sites that supported both VMMC and non-VMMC |  |
| Indirect costs | Facility operation, transport, waste management | Contracted Services | This includes contracted services (Cleaning and security services), IT services, office equipment, office space, renovation, and utilities: i.e. Water, electricity etc | Proportion of staff dedicated to VMMC program i.e. 33 out of 171 staff | 19% |
|  |  | Maintenance |  |  |  |
|  |  | Utilities |  |  |  |
| County Health Management Teams | Human resources for technical assistance & quality assurance (MOH) | Indirect Personnel cost | Staff costs paid by the county government to manage and oversee the program on behalf MOH. | Allocation based on the level of effort | Ranges between 10 to 25% |
| Direct staff-VMMC team | Human resources for routine services | Personnel | Total salary and other benefits for the surgical team was obtained from the payroll systems of UMB and CHS. The VMMC team comprised of VMMC advisor, Quality Assurance (QA) training officer, Early Infant Male Circumcision (EIMC) technical leads/trainers, drivers, Voluntary Medical Male Circumcision (VMMC) surgeon, surgeon and nurse | Allocation based on proportion of time spent on the program | 100% |
| Administrative staff | Human resources for technical assistance & quality assurance (IP/PEPFAR); includes IP HQ technical personnel | Indirect personnel | Proportion of time admin staff spend on VMMC program. The information was provided by CHS finance department and is based on the level of effort (LOE) | Allocation was based on level of effort for each staff to VMMC program (9% of administrative staff are VMMC-dedicated) | 9% |
| Motor vehicles (in Siaya) | Facility operation, transport, waste management | Travel | Expenditure includes cost of maintenance, fuel and vehicle rentals. For Siaya, CHS purchased vehicles for use in the VMMC program as well as in other programs in the county | Ideally, allocation should be based on mileage of vehicle use in the VMMC program or fuel consumption. However, this information was not readily available | We used the proportion of VMMC sites among total program sites to estimate expenditure attributed to VMMC program. This was 6 of 29, or 55%. |

Exhibit 2: Assumptions used in estimating baseline total and unit expenditures in Migori County

| ***Shared costs*** | **Maps to category(ies) in main manuscript figure 2- Explanation:** | ***De*scription** | ***Basis for allocation*** | ***Percentage*** |
| --- | --- | --- | --- | --- |
| Administrative costs (non-staff) | Administrative cost | Include rental costs, lease of printing equipment, general office supplies and office running costs. | Proportion of administrative costs attributed to a site | 100% allocation to the VMMC program |
|  | Administrative supplies |  |  |  |
| Meetings and trainings | Meetings | Sensitization meeting/mobilization, TWG meetings with the county, training of service providers, refresher training of service providers, performance review meetings, orientation meetings, start up and sustainability model meetings | Level of effort (time commitment in the VMMC program) | 100% of the costs allocated to the VMMC program |
|  | Training |  |  |  |
|  | Mobilization |  |  |  |
| Expenditure on medical Equipments. | Investment | Medical and non-medical equipment used specifically for the VMMC program | Proportion of usage of the equipment | 100% allocation of equipment costs to the VMMC program |
| Medicines, Medical Supplies and Consumables | Commodities: | All consumables including medicines and supplies used in the program e.g. gloves, swaps, surgical spirits etc. | Usage in the program | 100% costs allocated to the VMMC program |
| Other direct program costs | Other direct Costs | These include operational costs e.g. transport costs, hire of theatre for operation, allowances for VMMC staff, communication costs. | Based on usage | 100% of the costs allocated to the VMMC program |
|  | Airtime |  |  |  |
|  | Travel |  |  |  |
| Indirect costs | Utilities | Includes utilities e.g. water, electricity, maintenance, management costs | Based on level of usage in the program implementation | Maintenance costs (50% of total maintenance costs allocated to VMMC program), 9% of management costs allocated to the program, rent (9%), utilities (9%) |
|  | Maintenance |  |  |  |
| County Health Management Teams | Indirect Personnel cost | These county staff include the chief officer of health, county director of health, county health promotion officer, County AIDS and STI Coordinator (CASCO), VMMC focal person, Reproductive Health (RH) coordinator, nursing office, county public health officer etc. They coordinate and supervise implementation of health program, including VMMC, in the county | Based on the level of effort of specific county officer | Chief officer, county director, county records officer, county nursing officer, county medical technologist officer (10% of the total costs) |
|  |  |  |  | County health promotion officer and CASCO (25% of the total cost), county public officer (15%), VMMC focal person (80%) |
|  |  |  |  | Sub-county MoH officer (10%), sub-county health records officer (10 %,) sub-county RH coordinator, nursing officer, sub-county medical laboratory officer (10%), sub-county public health officer (15%) |
| [Salary for MoH VMMC team](file:///C:\Users\HP\Documents\2020\Project%20IQ-%20Clarification\Assumption%20reviewed%2003-11-2020.xlsx#RANGE!_ftn1) | Personnel | MoH in Migori receives grant from UMB to pay for staff costs. In Migori, the county government of Migori was paying staff involved in the VMMC program and therefore categorized as MoH costs | Allocation based on level of effort | 100% for the VMMC team |
| Administrative staff | Indirect personnel | Proportion of staff time spent on VMMC program. The information was provided by UMB finance department and is based on the level of effort (LOE). Includes IP HQ technical personnel as well. | Allocation was based on level of effort for each staff to VMMC program (9% of administrative staff are VMMC-dedicated) | 9% |

Exhibit 3: Percentage of salary allocation to VMMC by staff role **at baseline** in Migori County, for county-level MoH staff

| CHMT cadre | Level of effort to VMMC programme |
| --- | --- |
| CEC | 10% |
| Chief Officer for Health | 10% |
| County Director of Health | 10% |
| County Health Promotion Officers | 25% |
| CASCO | 25% |
| VMMC Team Lead-Focal Person | 80% |
| County Health Records | 10% |
| Reproductive Health Coordinator | 10% |
| County Nursing Officer | 10% |
| County Medical Laboratory Technologist | 10% |
| County PHO | 15% |

Exhibit 4: Percentage of salary allocation to VMMC by staff role **at baseline** in Migori County, for subcounty-level MoH staff

| CHMT cadre | Level of effort to VMMC programme |
| --- | --- |
| Subcounty (SC) MOH | 10% |
| SCASCO | 25% |
| Sub-county Promotion Officers | 0% |
| VMMC Implementing Officer | 0% |
| Sub-county Health Records Officer | 10% |
| Sub-county Reproductive Health Coordinator | 10% |
| Sub-county Nursing Officer | 10% |
| Sub-county PHO | 15% |
| Sub County Medical Laboratory Officer | 10% |

Exhibit 5: Percentage of salary allocation to VMMC by staff role **at baseline** in Migori County, for implementing partner (UMB) staff

| Program Director/ Co-PI | 9% |
| --- | --- |
| Finance and Operations manager | 9% |
| National office staff ratio | 9% |
| Senior Technical Advisor | 9% |
| Finance Officer | 9% |
| Program Officer | 9% |
| HR officer | 9% |
| Grants officer | 9% |
| Program Manager | 9% |
| Data Officers - M & E | 9% |
| Strategic Information Advisor | 9% |
| Admin Assistant | 9% |
| Driver | 100% |
| VMMC Advisor | 100% |
| QA/Training Officer | 100% |
| QA/Training Officer | 100% |
| EIMC Tech Leads/Trainers | 100% |
| Driver | 100% |
| NO/ VMMC Surgeon | 100% |
| RCO/ VMMC Surgeon | 100% |
| NO/ VMMC Surgeon | 100% |
| NO/ VMMC Surgeon | 100% |
| NO/ VMMC Surgeon | 100% |
| RCO/ VMMC Surgeon | 100% |
| Clinical Officer/ Surgeons | 100% |
| Clinical Officer/ Surgeons | 100% |
| Clinical Officer/ Surgeons | 100% |
| Scrub Nurse/ | 100% |
| Scrub Nurse | 100% |
| Scrub Nurse | 100% |
| RCO/ VMMC Surgeon | 100% |
| NO/ VMMC Surgeon | 100% |
| driver | 100% |
| Driver | 100% |
| RCO/ VMMC Surgeon | 100% |
| Driver | 100% |
| Driver | 100% |
| Clinical Officers | 100% |
| Registered Nurses | 100% |
| Hygiene Staff | 100% |
| Field Officers | 100% |
| Sub County Accounts & Logistic Officer | 100% |
| Counsellors | 100% |

Exhibit 6: Percentage of salary allocation to VMMC by staff role **at baseline** in Siaya County, for county-level MoH staff

| **Cadre** | **Level of effort *(Percentage of time in each staff is engaged in VMMC activities)*** |
| --- | --- |
| **COUNTY HEALTH MANAGEMENT TEAM** | |
| CEC | 10% |
| Chief Officer for Health | 10% |
| County Director of Health | 10% |
| County Promotion Officers | 10% |
| CASCO | 25% |
| VMMC Team Lead- Focal Person | 80% |
| Reproductive Health Coordinator | 10% |
| County Clinical Officer | 10% |
| County Nursing Officer | 10% |
| County PHO | 10% |
| County Community Strategy Focal Person | 10% |
| County Medical Laboratory Technologist | 10% |
| County Health Records and Information Officer (CHRIO) | 10% |

Exhibit 7: Percentage of salary allocation to VMMC by staff role **at baseline** in Siaya County, for subcounty-level MoH staff

| **SUB COUNTY HEALTH MANAGEMENT TEAM** | |
| --- | --- |
| **County: ALEGO USONGA** |  |
| SCMOH | 10% |
| SCASCO | 25% |
| Sub County Promotion Officers | 80% |
| Sub County Health Records | 10% |
| Sub-County Reproductive Health Coordinator | 10% |
| Sub County Nursing Officer | 10% |
| Sub County PHO | 10% |
| Sub-county Medical Laboratory Technologist | 10% |
| Community Strategy Focal Person | 10% |
|  |  |
|  |  |
| **County: UGENYA** |  |
| SCMOH | 10% |
| SCASCO | 25% |
| Sub County Promotion Officers | 80% |
| Sub County Health Records | 10% |
| Sub-County Reproductive Health Coordinator | 10% |
| Sub County Nursing Officer | 10% |
| Sub County PHO | 10% |
| Sub-county Medical Laboratory Technologist | 10% |
| Community Strategy Focal Person | 10% |
|  |  |
|  |  |
| **County: UGUNJA** |  |
| SCMOH | 10% |
| SCASCO | 25% |
| Sub County Promotion Officers | 80% |
| Sub County Health Records | 10% |
| Sub-County Reproductive Health Coordinator | 10% |
| Sub County Nursing Officer | 10% |
| Sub County PHO | 10% |
| Sub-county Medical Laboratory Technologist | 10% |
| Community Strategy Focal Person | 10% |
|  |  |
|  |  |
| **County: GEM** |  |
| SCMOH | 10% |
| SCASCO | 25% |
| Sub County Promotion Officers | 80% |
| Sub County Health Records | 10% |
| Sub-County Reproductive Health Coordinator | 10% |
| Sub County Nursing Officer | 10% |
| Sub County PHO | 10% |
| Sub-county Medical Laboratory Technologist | 10% |
| Community Strategy Focal Person | 10% |
|  |  |
| **Sub-Total** |  |
| **County: BONDO** |  |
| SCMOH | 10% |
| SCASCO | 25% |
| Sub County Promotion Officers | 80% |
| Sub County Health Records | 10% |
| Sub-County Reproductive Health Coordinator | 10% |
| Sub County Nursing Officer | 10% |
| Sub County PHO | 10% |
| Sub-county Medical Laboratory Technologist | 10% |
| Community Strategy Focal Person | 10% |
|  |  |
| **Sub-Total** |  |
| **County: RARIEDA** |  |
| SCMOH | 10% |
| SCASCO | 25% |
| Sub County Promotion Officers | 80% |
| Sub County Health Records | 10% |
| Sub-County Reproductive Health Coordinator | 10% |
| Sub County Nursing Officer | 10% |
| Sub County PHO | 10% |
| Sub-county Medical Laboratory Technologist | 10% |
| Community Strategy Focal Person | 10% |
|  |  |
|  |  |

Exhibit 8: Percentage of salary allocation to VMMC by staff role at baseline in Siaya County, for implementing partner (CHS) staff

| Position/Title | Level of effort to VMMC programme |
| --- | --- |
| Chief Executive Officer | 7% |
| Finance and Administration Director | 7% |
| Human Resources Manager | 6% |
| Grants Development Manager | 4% |
| Finance Manager | 7% |
| Contract & Grant Manager | 3% |
| Operations Manager | 3% |
| ICT Manager | 6% |
| Compliance Manager | 3% |
| Head of Programs | 11% |
| Evaluation Advisor | 11% |
| Programs Director | 22% |
| Deputy Project Director/STA | 22% |
| Deputy Project Director; Strategic Information & Systems | 22% |
| VMMC Advisor | 22% |
| QI Advisor | 22% |
| Senior Program Officer Bondo | 22% |
| Srn. Program Officer Gem | 22% |
| Srn. Program Officer Rarieda | 22% |
| Srn. Program Officer Siaya | 22% |
| Srn. Program Officer Ugenya/Ugunja | 22% |
| Program Officer-Early Infant Male Circumcision | 100% |
| Communication & Mobilization Advisor | 100% |
| Sub-county Mobilization Officers | 100% |
| Sub-county Mobilization Officers | 100% |
| Sub-county Mobilization Officers | 100% |
| Sub-county Mobilization Officers | 100% |
| Sub-county Mobilization Officers | 100% |
| Sub-county Mobilization Officers | 100% |
| Program Officer VMMC | 100% |
| Program Officer VMMC | 100% |
| Program Officer VMMC | 100% |
| Program Officer VMMC | 100% |
| Program Officer VMMC | 100% |
| Program Officer VMMC | 100% |
| VMMC Surgeons | 100% |
| VMMC Surgeons | 100% |
| VMMC Surgeons | 100% |
| VMMC Surgeons | 100% |
| VMMC Surgeons | 100% |
| VMMC Surgeons | 100% |
| VMMC Surgeons | 100% |
| VMMC Surgeons | 100% |
| VMMC Surgeons | 100% |
| VMMC Surgeons | 100% |
| VMMC Surgeons | 100% |
| VMMC Surgeons | 100% |
| VMMC Surgeons | 100% |
| VMMC Surgeons | 100% |
| VMMC Surgeons | 100% |
| VMMC Surgeons | 100% |
| QA/QC Coordinator | 100% |
| M&E Officer VMMC | 100% |
| M&E Officer VMMC | 100% |
| Data Officer VMMC | 100% |
| Data Officer VMMC | 100% |
| Data Officer VMMC | 100% |
| Senior Finance Officer | 22% |
| Grant Accountant | 22% |
| Compliance Officer | 6% |
| Compliance Officer | 6% |
| Human Resources Officer | 22% |
| Procurement Officer | 22% |
| Administration Officer | 22% |
| Admin assistant/receptionist | 22% |
| Logistics and stores assistant | 22% |
| ICT Officer | 22% |
| Contracts & Grants Officer (Sub) | 22% |
| Driver | 22% |
| Driver | 22% |
| Driver | 22% |
| Driver | 22% |
| Driver | 22% |
| Driver | 22% |
| Driver | 22% |
| Driver | 22% |
| Driver | 22% |
| Driver | 22% |

Exhibit 9: Assumptions used in estimating Y1 total and unit expenditures in Migori County

| **Direct Program Costs** | **Description** | **Basis for allocation** | **Percentage** |
| --- | --- | --- | --- |
| Direct staff-for routine VMMC services | Includes all costs related to hiring and maintaining human resources directly involved in delivery of VMMC services | All cost was allocated to VMMC program Staff were distributed across the various model of service delivery, thus have been grouped based on model implemented by each facility | 100% |
| Administrative costs | All administrative and program support cost are based on program LOE. All expenditures captured are those related to VMMC. These includes office related running cost, transport of general staff for general oversight purposes only, support supervisions etc. | Proportion of staff dedicated to VMMC program, proportion of MCs to other programs, space utilization of staff providing VMMC services and other programs etc. | 100% of costs allocated to VMMC program. Allocation into the various models of service delivery are based on workload (VMMCs done) |
| Meetings and trainings | All meeting and trainings undertaken by the sub-grantee are VMMC related. | All cost related to meeting and trainings are 100% | 100% allocation to VMMC program |
| Program expenses | All other program expenses, commodities and supplies, transport and travel and operating expenses for service delivery | Program expenses are 100% utilized to deliver VMMC services. | 100% |
| Motor Vehicle Maintenance | Includes all the cost of maintaining and running/operating program motor vehicles | All the cost captured reflects what was utilized by the program | 100% |
| Indirect staff costs | Cost of other all staff not directly involved in service provision but have LOE into the program. This include staff at Siaya field office and those providing technical assistance | Indirect staff who support VMMC are paid directly by the program, thus program contribution is reflected as 100% | 100% |
| Field office running costs | Includes all the costs involved in sustaining and running the field office. A percentage of the cost is attributed to the VMMC Program | This cost was already allocated to VMMC based on program LOE. Thus, the cost provided was factored in as 100% | 100% |
| **University of Maryland- HQ and field office (UMB)** | | | |
| Field office running costs | Includes all the costs involved in sustaining and running the field office. A percentage of the cost is attributed to the VMMC Program | This cost was already allocated to VMMC based on program LOE. | 100% |
| Contracted services | Includes all contractual costs to the third party to implement the VMMC i.e. costs of contracting SORI lake side community hospital to provide VMMC | All cost was factored in as 100%. The agreement is only to support implementation | 100% |
| Management of Severe Adverse Events (SAE) | Includes all the costs associated with managing all circumcision related complication. Part of this cost was met by UMB | The cost is 100% VMMC | 100% |
| Meetings and Training | Includes cost of all meetings and trainings related to VMMC; This cost includes facilitation costs during TWG meeting, attending trainings, external quality assessment of VMMC supported sites | All costs are 100% VMMC related | 100% |
| Facility running costs | Includes all utilities and overhead costs for running the MOH facilities within Migori County. The costs cover all services provided within health facilities; thus, allocation was necessary. | Allocation of the cost is based on workload |  |
| Support Supervision | Includes the cost of Support supervision done by CHMT and SCHMT. The costs cover all facilities within the County and Sub-County. | Allocation of the costs was based on LOE as provided by the CASCO see attached annex V1 | As indicated in the annex for CHMT |

Exhibit 10: Assumptions used in estimating Year 1 total and unit expenditures in Siaya County

| Direct Program Costs | Description | Basis for allocation | Percentage |
| --- | --- | --- | --- |
| Direct staff costs-VMMC Team | Includes all costs related to hiring and maintaining human resources directly involved in delivery of VMMC services | All costs are 100% VMMC. Staff are grouped into 16 roving teams, which rotate within the various models of service delivery | 100% |
| Program expenses | All other program expenses, commodities and supplies, transport and travel and operating expenses | VMMC is part of shinda program which is implemented by CHS in SIAYA County. Shinda program covers HIV care and treatment and VMMC programs. Some of the costs are 100% VMMC while others are shared. VMMC utilizes 30% of the shared costs as per LOE | 100% |
| Meetings and trainings | Includes all costs of meetings and trainings undertaken by CHS | All cost related to meeting and trainings are 100% | 100% |
| Motor Vehicle Maintenance | Includes all the cost of maintaining and running/operating program Motor vehicles | Allocation of these costs was based on program LOE. | 100% |
| **Indirect program costs** | | | |
| Field office running costs | Includes all the costs involved in sustaining and running the field office. A percentage of the cost is attributed to the VMMC Program | Allocation of the costs is based on program LOE | 30% |
| Administrative costs including overheads and utilities | All administrative and program support costs are based on program LOE. The costs include office related running cost; rent, utilities, transport, support supervision etc. | Allocation of the costs is based on program LOE | 30% |
| Indirect staff costs | Cost of all other staff not directly involved in service provision but have LOE into the program. This include staff at Siaya field office | Allocation of the costs is based on program LOE | 30% |
| **Ministry of health** | | | |
| Facility running costs | Includes all Utilities and overhead costs for running the MOH facilities within Siaya County. The costs cover all services provided within health facilities; thus, allocation was necessary. | Allocation of the cost is based on workload. |  |
| Support supervision | Includes the cost of Support supervision done by CHMT and SCHMT. The costs cover all facilities within the county and sub-county. | Allocation of the costs was based on LOE as provided by the CASCO see attached annex V1 | As indicated in the annex for CHMT |

**Exhibit 8.** Percentage of salary allocation to VMMC by staff role in **Year 1** in Migori County, for county-level MoH staff

| Cadre | No per cadre | Level of effort to VMMC programme |
| --- | --- | --- |
| CEC | 1 | 5% |
| Chief Officer for Health | 1 | 5% |
| County Director of Health | 1 | 10% |
| County Health Promotion Officers | 1 | 15% |
| CASCO | 1 | 25% |
| VMMC Team Lead- Focal Person | 1 | 35% |
| County Health Records | 1 | 10% |
| Reproductive Health Coordinator | 1 | 10% |
| County Nursing Officer | 1 | 8% |
| County Medical Laboratory Technologist | 1 | 10% |
| County PHO | 1 | 15% |

**Exhibit 9**. Percentage of salary allocation to VMMC by staff role in **Year 1** in Migori County, for subcounty-level MoH staff

| **NYATIKE SUB COUNTY** | No per cadre | Level of effort to VMMC programme |
| --- | --- | --- |
| SCMOH | 1 | 10% |
| SCASCO | 1 | 30% |
| Sub County Promotion Officers | 1 | 15% |
|  | - | 0% |
| Sub County Health Records | 1 | 20% |
| Sub-County Reproductive Health Coordinator | 1 | 8% |
| Sub County Nursing Officer | 1 | 5% |
| Sub County PHO | 1 | 10% |
| Sub-Count Medical Laboratory Officer | 1 | 15% |
|  |  |  |
| **RONGO SUB COUNTY** |  |  |
| SCMOH | 1 | 10% |
| SCASCO | 1 | 25% |
| Sub County Promotion Officers | 1 | 0% |
| VMMC Implementing Officer | - | 0% |
| Sub County Health Records | 1 | 10% |
| Sub-County Reproductive Health Coordinator | 1 | 10% |
| Sub County Nursing Officer | 1 | 10% |
| Sub County PHO | 1 | 15% |
| Sub-Count Medical Laboratory Officer | 1 | 10% |
|  |  |  |
| **SUNA WEST SUB-COUNTY** |  |  |
| SCMOH | 1 | 10% |
| SCASCO | 1 | 25% |
| Sub County Health Promotion Officers | 1 | 0% |
| VMMC Implementing Officer | - | 0% |
| Sub County Health Records | 1 | 10% |
| Sub-County Reproductive Health Coordinator | 1 | 10% |
| Sub County Nursing Officer | 1 | 10% |
| Sub County PHO | 1 | 15% |
| Sub-Count Medical Laboratory Officer | 1 | 10% |
|  |  |  |
| **Other Indirect staff** |  |  |
| Finance Officer | 1 | 10% |
| HR Officer | 1 | 10% |

**Exhibit 10:** Percentage of salary allocation to VMMC by staff role in **Year 1** in Siaya County, for county-level and sub-county-level MoH staff

| **County Health Management Team** | **No per cadre** | Level of effort to VMMC programme |
| --- | --- | --- |
|  |  |  |
| CEC | 1 | 10% |
| Chief Officer for Health | 1 | 10% |
| County Director of Health | 1 | 10% |
| County Health Promotion Officers | 1 | 10% |
| CASCO | 1 | 10% |
| VMMC Team Lead- Focal Person | 1 | 10% |
| County Health Records | 1 | 10% |
| Reproductive Health Coordinator | 1 | 10% |
| County Nursing Officer | 1 | 10% |
| County Medical Laboratory Technologist | 1 | 10% |
| County PHO | 1 | 10% |
|  |  |  |
|  |  |  |
| **Sub County Health Management Team-Alego** | |  |
|  |  |  |
| SCMOH | 1 | 10% |
| SCASCO | 1 | 10% |
| Sub County Promotion Officers | 1 | 10% |
| VMMC Implementing Officer |  |  |
| Sub County Health Records | 1 | 10% |
| Sub-County Reproductive Health Coordinator |  |  |
| Sub County Nursing Officer | 1 | 10% |
| Sub County PHO | 1 | 10% |
| Sub-Count Medical Laboratory Officer | 1 | 10% |
|  |  |  |
|  |  |  |
| Sub County Health Management Team-Gem | |  |
|  |  |  |
| SCMOH | 1 | 10% |
| SCASCO | 1 | 10% |
| Sub County Promotion Officers | 1 | 10% |
| VMMC Implementing Officer |  |  |
| Sub County Health Records | 1 | 10% |
| Sub-County Reproductive Health Coordinator |  |  |
| Sub County Nursing Officer | 1 | 10% |
| Sub County PHO | 1 | 10% |
| Sub-Count Medical Laboratory Officer | 1 | 10% |
|  |  |  |
|  |  |  |
| Sub County Health Management Team-Rarieda | |  |
|  |  |  |
| SCMOH | 1 | 10% |
| SCASCO | 1 | 10% |
| Sub County Promotion Officers | 1 | 10% |
| VMMC Implementing Officer |  |  |
| Sub County Health Records | 1 | 10% |
| Sub-County Reproductive Health Coordinator |  |  |
| Sub County Nursing Officer | 1 | 10% |
| Sub County PHO | 1 | 10% |
| Sub-Count Medical Laboratory Officer | 1 | 10% |
|  |  |  |
|  |  |  |
| Sub County Health Management Team-Bondo | |  |
|  |  |  |
| SCMOH | 1 | 10% |
| SCASCO | 1 | 10% |
| Sub County Promotion Officers | 1 | 10% |
| VMMC Implementing Officer |  |  |
| Sub County Health Records | 1 | 10% |
| Sub-County Reproductive Health Coordinator |  |  |
| Sub County Nursing Officer | 1 | 10% |
| Sub County PHO | 1 | 10% |
| Sub-Count Medical Laboratory Officer | 1 | 10% |
|  |  |  |
|  |  |  |
| Sub County Health Management Team-Ugenya | |  |
|  |  |  |
| SCMOH | 1 | 10% |
| SCASCO | 1 | 10% |
| Sub County Promotion Officers | 1 | 10% |
| VMMC Implementing Officer |  |  |
| Sub County Health Records | 1 | 10% |
| Sub-County Reproductive Health Coordinator |  |  |
| Sub County Nursing Officer | 1 | 10% |
| Sub County PHO | 1 | 10% |
| Sub-Count Medical Laboratory Officer | 1 | 10% |
|  |  | Sub Total |
|  |  |  |
| Sub County Health Management Team-Ugunja | |  |
|  |  |  |
| SCMOH | 1 | 10% |
| SCASCO | 1 | 10% |
| Sub County Promotion Officers | 1 |  |
| VMMC Implementing Officer |  | 10% |
| Sub County Health Records | 1 | 10% |
| Sub-County Reproductive Health Coordinator |  |  |
| Sub County Nursing Officer | 1 | 10% |
| Sub County PHO | 1 | 10% |
| Sub-Count Medical Laboratory Officer | 1 | 10% |
| Other Indirect staff |  |  |
| Finance Officer | 4 | 10% |
| HR Officer | 2 | 10% |

**Exhibit 11.** Percentage of salary allocation to VMMC by staff role in **Year 1** in Migori County**, Partner-Supported staff: University of Maryland Baltimore (UMB)**

| **Staff Carder** | LOE to the VMMC program |
| --- | --- |
| Finance Manager | 3% |
| Finance Manager | 3% |
| Program Director | 3% |
| Programs Manager | 3% |
| Administrative Officer | 5% |
| Administrator | 5% |
| Adminstrative Officer, Director's office | 5% |
| Assistant Accountant-Admin | 5% |
| Clinical Manager | 5% |
| ICT Manager | 5% |
| ICT Manager | 5% |
| Assistant Accountant-Admin | 5% |
| Administrator | 5% |
| Administrative Officer | 5% |
| Data Manager | 5% |
| Deputy Technical Advisor, EIMC | 5% |
| MER Manager | 5% |
| ICT Manager | 5% |
| ICT Manager | 5% |
| Stores Officer | 5% |
| Head of Internal Audit | 5% |
| Head of Performance Monitoring Team | 5% |
| Assistant Accountant-Admin | 5% |
| MER Manager | 5% |
| Stores Officer | 5% |
| Adminstrative Officer, Director's office | 5% |
| Administrator | 5% |
| Procurement Officer | 5% |
| Stores Officer | 5% |
| Data Reporting Officer | 10% |
| Data Reporting Officer | 10% |
| Data Reporting Officer | 10% |
| Data Reporting Officer | 10% |
| Data Reporting Officer | 10% |
| Project Internal Auditor | 50% |
| Deputy Technical Advisor, QA/QC/Training | 59% |
| Deputy Technical Advisor, QA/QC/Training | 59% |
| Technical Advisor, VMMC | 59% |
| Deputy Technical Advisor, QA/QC/Training | 59% |
| Technical Advisor, VMMC | 59% |
| Sub-county Account & Logistics Officer (ALO) | 100% |
| Sub-county Account & Logistics Officer (ALO) | 100% |
| Sub-county Account & Logistics Officer (ALO) | 100% |
| Team Leader - Clinical Officer (Suna East) | 100% |
| Clinical Officer | 100% |
| Clinical Officer | 100% |
| Clinical Officer | 100% |
| Team Leader - Clinical Officer | 100% |
| Locum-Clinical Officer | 100% |
| Locum-Clinical Officer | 100% |
| Team Leader - Clinical Officer (Nyatike) | 100% |
| Team Leader - Clinical Officer | 100% |
| MC Counselor | 100% |
| Nursing Officer | 100% |
| Sub-county Account & Logistics Officer (ALO) | 100% |
| Field Assistants/Divisional Mobilizers | 100% |
| Hygiene Officers (Infection Prevention Officers) | 100% |
| HMIS Officer | 100% |
| Field Assistants/Divisional Mobilizers | 100% |
| Nursing Officer | 100% |
| Team Leader - Nursing Officer (Rongo/Uriri) | 100% |
| Team Leader - Nursing Officer (Rongo/Uriri) | 100% |
| Team Leader - Nursing Officer (Rongo/Uriri) | 100% |
| Hygiene Officers (Infection Prevention Officers) | 100% |
| Locum-Clinical Officer | 100% |
| MC Counselor | 100% |
| MC Counselor | 100% |
| MC Counselor | 100% |
| Nursing Officer | 100% |
| MC Counselor | 100% |
| MC Counselor | 100% |
| MC Counselor | 100% |
| MC Counselor | 100% |
| Field Assistants/Divisional Mobilizers | 100% |
| Sub-county Account & Logistics Officer (ALO) | 100% |
| Field Assistants/Divisional Mobilizers | 100% |
| Team Leader - Nursing Officer | 100% |
| Field Assistants/Divisional Mobilizers | 100% |
| Nursing Officer | 100% |
| Team Leader - Nursing Officer (Rongo/Uriri) | 100% |
| Field Assistants/Divisional Mobilizers | 100% |
| Field Assistants/Divisional Mobilizers | 100% |
| Hygiene Officers (Infection Prevention Officers) | 100% |
| Field Assistants/Divisional Mobilizers | 100% |
| Field Assistants/Divisional Mobilizers | 100% |
| Regional Coordinator | 100% |
| Driver Mechanic | 100% |
| Locum-Hygiene Officers (IPO) | 100% |
| Driver | 100% |
| Driver | 100% |
| Field Assistants/Divisional Mobilizers | 100% |
| Field Officers/Sub-County Social Mobilizers | 100% |
| Field Assistants/Divisional Mobilizers | 100% |
| Field Assistants/Divisional Mobilizers | 100% |
| Field Assistants/Divisional Mobilizers | 100% |
| Field Officers/Sub-County Social Mobilizers | 100% |
| Field Assistants/Divisional Mobilizers | 100% |
| Field Assistants/Divisional Mobilizers | 100% |
| Field Assistants/Divisional Mobilizers | 100% |
| Field Assistants/Divisional Mobilizers | 100% |
| Field Assistants/Divisional Mobilizers | 100% |
| Field Assistants/Divisional Mobilizers | 100% |
| Field Assistants/Divisional Mobilizers | 100% |
| Field Officers/Sub-County Social Mobilizers | 100% |
| Field Assistants/Divisional Mobilizers | 100% |
| Locum-Field Assistants/Super Mobilizer | 100% |
| Field Assistants/Divisional Mobilizers | 100% |
| Field Assistants/Divisional Mobilizers | 100% |
| Nursing Officer | 100% |
| Field Assistants/Divisional Mobilizers | 100% |
| Field Assistants/Divisional Mobilizers | 100% |
| Field Assistants/Divisional Mobilizers | 100% |
| Field Assistants/Divisional Mobilizers | 100% |
| Field Assistants/Divisional Mobilizers | 100% |
| Nursing Officer | 100% |
| Nursing Officer | 100% |
| Hygiene Officers (Infection Prevention Officers) | 100% |
| Locum-Field Assistants/Super Mobilizer | 100% |
| Locum-Clinical Officer | 100% |
| Field Assistants/Divisional Mobilizers | 100% |
| HMIS Officer | 100% |
| HMIS Officer | 100% |
| Hygiene Officers (Infection Prevention Officers) | 100% |
| Hygiene Officers (Infection Prevention Officers) | 100% |
| Hygiene Officers (Infection Prevention Officers) | 100% |
| Hygiene Officers (Infection Prevention Officers) | 100% |
| Hygiene Officers (Infection Prevention Officers) | 100% |
| Hygiene Officers (Infection Prevention Officers) | 100% |
| Hygiene Officers (Infection Prevention Officers) | 100% |
| Hygiene Officers (Infection Prevention Officers) | 100% |
| Team Leader - Nursing Officer | 100% |
| Team Leader - Clinical Officer (Nyatike) | 100% |
| Field Assistants/Divisional Mobilizers | 100% |
| Clinical Officer | 100% |
| Clinical Officer | 100% |
| Field Assistants/Divisional Mobilizers | 100% |
| Clinical Officer | 100% |
| Nursing Officer | 100% |
| Field Assistants/Divisional Mobilizers | 100% |
| Team Leader - Nursing Officer (Rongo/Uriri) | 100% |
| Field Assistants/Divisional Mobilizers | 100% |
| Field Assistants/Divisional Mobilizers | 100% |
| Locum-Hygiene Officers (IPO) | 100% |
| Hygiene Officers (Infection Prevention Officers) | 100% |
| Project Accountant | 100% |
| Nursing Officer | 100% |
| Team Leader - Nursing Officer | 100% |
| Team Leader - Nursing Officer | 100% |
| Nursing Officer | 100% |
| Nursing Officer | 100% |
| Team Leader - Nursing Officer (Rongo/Uriri) | 100% |
| Nursing Officer - EIMC | 100% |
| MC Counselor | 100% |
| Field Assistants/Divisional Mobilizers | 100% |
| Field Assistants/Divisional Mobilizers | 100% |
| Field Assistants/Divisional Mobilizers | 100% |
| Sub-county Account & Logistics Officer (ALO) | 100% |
| Team Leader - Nursing Officer (Rongo/Uriri) | 100% |
| Team Leader - Nursing Officer (Rongo/Uriri) | 100% |
| Hygiene Officers (Infection Prevention Officers) | 100% |
| Driver | 100% |
| Field Assistants/Divisional Mobilizers | 100% |

**Exhibit 12. Percentage of salary allocation to VMMC by staff role in Year 1 in Siaya County, Partner-Supported staff: Centre for Health Solutions (CHS)**

| Grants & Contracts Officer (Sub) | 15% |
| --- | --- |
| Logistics & Stores Assistant | 20% |
| Driver | 30% |
| Driver | 30% |
| Driver | 30% |
| Driver | 30% |
| Driver | 30% |
| Driver | 30% |
| Driver | 30% |
| Driver | 30% |
| Driver | 30% |
| Driver | 30% |
| Driver | 30% |
| Driver | 30% |
| Driver | 30% |
| Driver | 30% |
| Driver | 30% |
| Driver | 30% |
| Driver | 30% |
| Driver | 30% |
| Driver | 30% |
| Driver | 30% |
| Driver | 30% |
| Driver | 30% |
| Driver | 30% |
| Driver | 30% |
| Driver | 30% |
| ASS. SURGEON | 100% |
| ASS. SURGEON | 100% |
| ASS. SURGEON | 100% |
| ASS. SURGEON | 100% |
| ASS. SURGEON | 100% |
| ASS. SURGEON | 100% |
| ASS. SURGEON | 100% |
| ASS. SURGEON | 100% |
| ASS. SURGEON | 100% |
| ASS. SURGEON | 100% |
| ASS. SURGEON | 100% |
| ASS. SURGEON | 100% |
| ASS. SURGEON | 100% |
| ASS. SURGEON | 100% |
| ASS. SURGEON | 100% |
| ASS. SURGEON | 100% |
| Communication & Mobilization Advisor | 100% |
| INFECTION PREVENTION OFFICER | 100% |
| INFECTION PREVENTION OFFICER | 100% |
| INFECTION PREVENTION OFFICER | 100% |
| INFECTION PREVENTION OFFICER | 100% |
| INFECTION PREVENTION OFFICER | 100% |
| INFECTION PREVENTION OFFICER | 100% |
| INFECTION PREVENTION OFFICER | 100% |
| INFECTION PREVENTION OFFICER | 100% |
| INFECTION PREVENTION OFFICER | 100% |
| INFECTION PREVENTION OFFICER | 100% |
| INFECTION PREVENTION OFFICER | 100% |
| INFECTION PREVENTION OFFICER | 100% |
| INFECTION PREVENTION OFFICER | 100% |
| INFECTION PREVENTION OFFICER | 100% |
| INFECTION PREVENTION OFFICER | 100% |
| INFECTION PREVENTION OFFICER | 100% |
| Program Officer -VMMC | 100% |
| Program Officer-VMMC | 100% |
| Program Officer-VMMC | 100% |
| Sub County Mobilization Officer | 100% |
| Sub County Mobilization Officer | 100% |
| Sub County Mobilization Officer | 100% |
| Sub County Mobilization Officer | 100% |
| Sub County Mobilization Officer | 100% |
| VMMC COUNSELOR | 100% |
| VMMC COUNSELOR | 100% |
| VMMC COUNSELOR | 100% |
| VMMC COUNSELOR | 100% |
| VMMC COUNSELOR | 100% |
| VMMC COUNSELOR | 100% |
| VMMC COUNSELOR | 100% |
| VMMC COUNSELOR | 100% |
| VMMC COUNSELOR | 100% |
| VMMC COUNSELOR | 100% |
| VMMC COUNSELOR | 100% |
| VMMC COUNSELOR | 100% |
| VMMC COUNSELOR | 100% |
| VMMC COUNSELOR | 100% |
| VMMC COUNSELOR | 100% |
| VMMC COUNSELOR | 100% |
| VMMC DATA CLERKS | 100% |
| VMMC DATA CLERKS | 100% |
| VMMC DATA CLERKS | 100% |
| VMMC DATA CLERKS | 100% |
| VMMC surgeon | 100% |
| VMMC surgeon | 100% |
| VMMC surgeon | 100% |
| VMMC surgeon | 100% |
| VMMC surgeon | 100% |
| VMMC surgeon | 100% |
| VMMC surgeon | 100% |
| VMMC surgeon | 100% |
| VMMC surgeon | 100% |
| VMMC surgeon | 100% |
| VMMC surgeon | 100% |
| VMMC surgeon | 100% |
| VMMC surgeon | 100% |
| VMMC surgeon | 100% |
| VMMC surgeon | 100% |
| VMMC surgeon | 100% |
| VMMC surgeon | 100% |
| VMMC surgeon | 100% |
| VMMC surgeon | 100% |

​
