# Supplementary material for: Making voluntary medical male circumcision services sustainable: Findings from Kenya’s pilot models, baseline and year 1
Source: PLoS One. 2021 Jun 11;16(6):e0252725. doi: 10.1371/journal.pone.0252725 (PMC8195380; doi:10.1371/journal.pone.0252725)
Supplement: S7 Appendix — (DOCX) [file pone.0252725.s007.docx]

**Identifying sustainable service delivery models to maintain medical male circumcision coverage in Western Kenya**

**Results of Qualitative Surveys**

**Baseline and Year 1**

**Introduction and Background**

As VMMC priority countries begin approaching the PEPFAR target of 80% male circumcision coverage among 15-29-year-olds, they need to plan for a transition to long-term coverage maintenance entirely under national and local leadership, oversight and financing. Sustainable medical male circumcision service delivery models that can sustain high circumcision coverage and quality of services do not currently exist. Kenya is a global leader in achieving VMMC coverage targets and has the potential to be a leader in moving to full country leadership and ownership of sustainable VMMC services. Consistent with PEPFAR 3.0’s evolution towards a more sustainable HIV/AIDS response, PEPFAR will support Kenya to develop “affordable, quality, locally owned (managed, implemented and funded)”^[[1]](#endnote-1)^ VMMC models for the counties implementing VMMC.

Such sustainable models will need to deliver VMMC targeting adolescents aged 10-14 years, either long-term or as a bridge to early infant male circumcision, (through adolescent circumcision for the next decade, until the cohort of boys circumcised as infants age into that group, given that male circumcision under local anesthesia is not recommended for males aged > 60 days to < 10 years.) These approaches differ from the current catch-up phase of VMMC: they will be characterized by lower and more consistent volume, diffuse geographic distribution, pre-existing high-potential venues (schools and other programs) to facilitate access to clients, and the high intrinsic demand for VMMC typical among adolescent males. These characteristics make country-based financing more feasible, if the model used is lower-cost and better-positioned to take advantage of them through links to these venues. Finally, because implementing counties have substantial internal variations in characteristics such as population density and ease of access to health care, no single model may be appropriate throughout any county; approaches adapted to the local area may be needed.

Aims of the Program Evaluation and evaluation questions

Over three years (2017 – 2020), the evaluation will monitor quantitative and qualitative indicators of VMMC service delivery sustainability for three VMMC service delivery models for 10-14-year-olds designed for different areas of Kenya, to determine if each can maintain adolescent client demand/acceptability, minimize costs and health system burden, optimize program and HIV/AIDS indicator data availability, and facilitate government ownership. The specific evaluation questions to be answered for each model are:

- 1. Can this model deliver high-quality VMMC services that are sufficient in volume to maintain over 80% coverage among 10-14 year olds?
  2. Can this model be sustainably implemented by the Government of Kenya using its own financial, human, and management/oversight resources?
  3. What are the areas of weakness that need to be improved in order to maximize this model’s sustainability?

This report presents results from qualitative surveys that were conducted to answer question ‘c’.

## **VMMC Service Delivery Models**

The three models being implemented and evaluated are all within the scope of current VMMC practice in Kenya. They are:

1. A ‘static’ model in which general clinicians stationed in health facilities offer VMMC to clients who present requesting it, paired with targeted demand creation and mobilization at, and potentially transportation assistance from and back to schools and other adolescent venues. .
2. A ‘mixed’ model similar to the most common current approach, in which providers offer adolescent-targeted rapid results initiative (RRI)-type demand creation and services at opportune times in the school year, including use of schools as venues and emphasis on demand creation as necessary; and possibly provide services similar to the static model during the rest of the year..
3. A ‘mobile’ model, in which a mobile VMMC-dedicated team is responsible for maintaining VMMC coverage, including performing mobilization and demand creation, over a large catchment area via year-round short visits to multiple venues.

Design/Methods

This is a cross-sectional, retrospective (baseline) and prospective in-depth qualitative case-study using a self-administered Likert-scale questionnaire. Respondents were drawn from various levels of VMMC service delivery ranging from the national/county leadership to the community level and were sampled using a convenience sampling technique.

Data was collected at two time points – March to April 2018 for baseline (Oct 2016 – Nov 2017) and in August 2018 for Year 1(Dec 2017 – August 2018) assessment. Three self-administered tools were distributed to respondents as shown in the table below:

Table 1: Respondents and Sample Size Distribution

| **Tool** | **Audience** | **Distribution of Sample size by cadres** | **Minimum Sample size per county** | **Sample size per county**  **Baseline** | **Sample size per county**  **Year 1** |
| --- | --- | --- | --- | --- | --- |
| Tool 4  KII – Leadership | IP and County Leadership   1. National VMMC leadership, CDC VMMC lead 2. IP’s and County Leadership (MOH)-CASCO, VMMC focal person, county health promotion officer, County HRIO, Program director (UMB), Technical advisor (UMB), County Director of Health. | 1 – national level  1 – CDC VMMC lead  7 – county level | Migori =9  Siaya =9 | Migori = 6  Siaya = 1 | Migori = 1  Siaya = 5 |
| Tool 5  KII – Project | 1. Staff directly involved in model oversight. 2. Site level leadership    1. VMMC site manager    2. VMMC site staff - counsellor, assistant surgeon, infection prevention 3. In charges for all facilities. | Each team comprises of 5 members | Migori=40  Siaya = 40 | Migori =  Siaya = | Migori =  Siaya = |
| Tool 6  KII – Community | Community respondents   1. 4-6 residents with knowledge or experience with VMMC services from villages in the cluster areas 2. School administrators and teachers – head teachers and all Class teachers whose classes are involved. 3. Religious leaders from villages in the cluster areas 4. Local leaders from the villages in the cluster areas (head men, sub-chiefs, chiefs) | 5 residents per ward  1 school administrator per ward  1 teacher per ward  1 religious leader per ward  1 local leader per ward | Migori =126  Siaya = 99 | Migori =  Siaya = | Migori =  Siaya = |

**Analysis**

Responses from the questionnaires which used a 5 point Likert scale were analyzed. A score of 1 reflected poor performance or a weakness in that particular area (interpreted as less conducive to sustainability) and 5 indicated better performance or a strength in that particular area (good for sustainability). Mean scores above 3 were considered to the strengths and those below 3 weaknesses. A summary of the top 5 strengths and 5 weaknesses for each service delivery model, at different levels of the health system was created.

**Key Findings**

**TOOL 4: COUNTY AND NATIONAL LEVEL LEADERSHIP**

This tool was completed by county and national leaders who had a role in the implementation of the survey. The following domain and sub-domains were assessed for sustainability:

1. Governance, leadership and Accountability
   1. Planning and Coordination (P&C)
   2. Civil Society Management (CSM)
   3. Transparency (T)

**Summary of Key strengths and weaknesses:**

Results from surveys with the County and National level leadership showed that at baseline, which was the year before the Sustainability project was implemented in Migori and Siaya counties, the key strengths of the VMMC project included management by a competent county health team and well-coordinated activities with effective communication between the county health team and implementing partners. All stakeholders were aware of the goal of maintaining 80% coverage and there was a demand creation strategy in place to meet the coverage targets.

One year after the Sustainability project was launched (Year 1), these strengths were maintained. In addition, the County health team continued to strengthen their planning and coordination activities by actively leading mechanisms and processes that routinely convene stakeholders for VMMC planning and coordination purposes and ensuring that current and future staffing needs were based on the VMMC program goals and targets.

The results also identified weaknesses with the VMMC program. The major weakness at baseline were that the roles and responsibilities between the county health team and IP’s were not clearly defined; poor civil society engagement; budgets were not well allocated and there was a lack of transparency when it came to public expenditure reporting and sharing of achievements. These weaknesses continued in Year 1 with the only improvement being that roles and responsibilities were clearly defined in Year 1.

| **Tool 4: County and National Level Leadership*** | | |
| --- | --- | --- |
|  | **Baseline  (n=7)** | **Year 1 (n=6)** |
| Strengths | 1. *The goal of the VMMC sustainability project (maintaining 80% coverage) is well understood by all stakeholders.* 2. *Communication between the VMMC sustainability project team and the county health team is effective.* 3. The county health team responsible for supervising, monitoring and supporting the VMMC sustainability project are qualified and competent to do so. 4. The VMMC sustainability project has an effective demand creation strategy for meeting performance targets. 5. There are clear and transparent processes for the hiring of staff for the VMMC sustainability project. | 1. *The goal of the VMMC sustainability project (maintaining 80% coverage) is well understood by all stakeholders.* 2. *Communication between the VMMC sustainability project team and the county health team is effective* 3. The county health team actively leads a mechanism or process (i.e., committee, working group, etc.) that routinely convenes stakeholders for VMMC planning and coordination purposes. 4. The county health team is committed to maintaining 80% VMMC coverage in the site's catchment area. 5. Current and future staffing needs are based on the VMMC program goals and targets. |
| Weaknesses | 1. *The VMMC sustainability project team does not make VMMC expenditure summary reports available to stakeholders and the general public regularly.* 2. *The county health team does not develop budgets that allocate resources to high need VMMC service delivery locations*. 3. VMMC program achievements are not shared with stakeholders and the general public regularly. 4. The county health team does not always communicate the need for the program and provides important information about the VMMC program to community leaders. 5. VMMC management roles and responsibilities between the county health team and all implementing partners are not clearly defined. | 1. *The VMMC sustainability project team does not make VMMC expenditure summary reports available to stakeholders and the general public regularly.* 2. *The county health team does not develop budgets that allocate resources to high need VMMC service delivery locations*. 3. The county health team does not engage with civil society in VMMC program planning and client recruitment for VMMC. 4. The county health team does not engage with civil society in program evaluation and getting feedback from VMMC clients. |

**Responses in italics remained constant from Baseline to Year 1.*

**TOOL 5: KEY INFORMANT QUESTIONNAIRE – PROJECT AND SITE LEVEL**

This tool was completed by staff directly involved in sustainability model oversight (including MoH staff), site program leadership (VMMC site manager) and VMMC site staff. The following domains were assessed for sustainability:

1. Governance, leadership and Accountability
   1. Planning and Coordination (P&C)
   2. Civil Society Management (CSM)
   3. Transparency (T)
2. National Health System and Service Delivery
   1. Domestic Service Delivery (DSD)
   2. Supply Chain (SC)
   3. Quality Management (QM)
3. Strategic Investments, Efficiency and Sustainable Financing
   1. Domestic Resource Mobilization (DRM)
   2. Technical and Allocative Efficiencies (TAE)
4. Strategic Information
   1. Performance Data (PD)

Below are results of the top 5 strengths and weaknesses across domains for all models as well as for each model.

**Strengths**: At baseline, Domestic Service Delivery and Quality Management of VMMC services were strengths across all models. Supply Chain processes worked well and site level performance data was submitted through national reporting systems. These continued to be strengths in Year 1. In addition, staff training and certification was identified as a strength in Year 1.

**Weaknesses**: At baseline, across all models, Project and site level respondents reported that a major weakness of the VMMC program was the dependence on external technical assistance and funding sources. There were still waiting lists for VMMC and routine service delivery was still not task-shifted to the lowest cadre. In Year 1, the major weakness identified were low levels of civil society engagement and few community groups engaged in VMMC programs.

| **Tool 5: Project and Site Level _ All Models** | | |
| --- | --- | --- |
|  | **Baseline** | **Year 1 (n=46)** |
| **Strengths** | 1. *VMMC is offered regularly as planned.(DSD)* 2. *Performance against VMMC quality standards is reviewed at least quarterly with the service delivery team.(QM)* 3. VMMC services do not interfere with other health services provided at the site. (DSD) 4. Supply processes work well enough to provide everything needed to ensure client safety and achieve VMMC targets.(SC) 5. Site-level VMMC performance data is submitted to the county health team through the national reporting system. (SI) | 1. *VMMC is offered regularly as planned. (DSD)* 2. *Performance against VMMC quality standards is reviewed at least quarterly with the service delivery team. (QM)* 3. Recruitment of clients for VMMC does not interfere with any services provided at the recruitment venues. (DSD) 4. VMMC services are offered in a manner acceptable to the community (DSD) 5. All VMMC field staff are trained and certified to perform their duties as assigned. (HRH) 6. Staff have the training and capacity to apply VMMC quality improvement methods (QM) |
| **Weaknesses** | 1. The county health team needs external technical assistance to execute their VMMC responsibilities 2. Domestic funding sources for VMMC program support have not been identified. 3. Routine VMMC service delivery is not task-shifted to the lowest permitted cadre 4. There are waiting lists for VMMC services. 5. The county health team does not develop budgets that allocate resources to high need VMMC service delivery locations. | 1. Do not have diverse community groups that are committed to the success of the VMMC sustainability project. |

Summary of key strengths and major weaknesses across domains for the mixed model.

**Strengths**: At baseline, Strategic Information, Domestic Service Delivery and Quality management of VMMC services were identified as strengths of the mixed model. VMMC services were offered regularly and were accessible to clients; the service delivery team reviewed performance against targets and quality standards quarterly and site-level performance data was submitted to the county health team. These continued to be strengths in Year 1. In addition, in Year 1, Planning and Coordination of VMMC services emerged as a key strength of this model. The competency and qualifications of the county health team and the use of data to plan staffing and VMMC services was identified as a key strength.

**Weaknesses**: At baseline, project and site level respondents reported that a major weakness of the VMMC program was the dependence on external technical assistance and funding sources. Budgets were not well allocated and routine service delivery was still not task-shifted to the lowest cadre. In Year 1, the major weaknesses identified during the baseline period were addressed. The major weakness identified during Year 1 was that there were still waiting lists for VMMC in the mixed model areas.

| **Tool 5: Project and Site Level _ Mixed Model** | | |
| --- | --- | --- |
|  | **Baseline  (n=21)** | **Year 1 (n=12)** |
| Strengths | 1. Site-level VMMC performance data is submitted to the county health team through the national reporting system (P & C) 2. VMMC is offered regularly as planned.(DSD) 3. VMMC services are easily accessible to interested clients (DSD). 4. Performance against VMMC targets is reviewed at least quarterly with the service delivery team.(QM) 5. Performance against VMMC quality standards is reviewed at least quarterly with the service delivery team.(QM) | 1. VMMC services are offered in a manner acceptable to the community (DSD) 2. Recruitment of clients for VMMC does not interfere with any services provided at the recruitment venues.(DSD) 3. The county health team uses data to measure the effectiveness of the VMMC project in delivering needed VMMC services in the right locations.(P&C) 4. The county health team responsible for supervising, monitoring and supporting the VMMC sustainability project are qualified and competent to do so.(P&C) 5. Current and future staffing needs are based on the VMMC program goals and targets.(P&C) |
| Weaknesses | 1. Domestic funding sources for VMMC program support have not been identified. 2. The county health team needs external technical assistance to executes their VMMC responsibilities. 3. The county health team does not develop budgets that allocate resources to high need VMMC service delivery locations. 4. Staff from this cadre do not routinely perform all VMMC surgical/procedural steps without on-site supervision from a higher cadre. | 1. There are waiting lists for VMMC services. |

Summary of key strengths and major weaknesses across domains for the mobile model.

**Strengths**: At baseline, Domestic Service Delivery was identified as a major strength of the mobile model. Recruitment of clients as well as provision of VMMC services did not interfere with other services. In addition staff were trained and certified and site-level performance data was submitted to the county health team. These continued to be strengths in Year 1. In addition, by Year 1, Quality Management and Planning and Coordination of VMMC services emerged as a key strength of this model.

**Weaknesses**: At baseline, project and site level respondents reported these major weaknesses were identified – county health team did not allocate resources by need and the VMMC program was dependent on external technical assistance and funding sources. In Year 1, most of the major weaknesses identified during the baseline period were addressed. However, the county health team still did not develop budgets that allocated resources by need.

| **Tool 5: Project and Site Level _ Mobile Model** | | |
| --- | --- | --- |
| Section | **Baseline  (n=23)** | **Year 1 (n=20)** |
| Strengths | 1. *Recruitment of clients for VMMC does not interfere with any services provided at the recruitment venues.(DSD)* 2. VMMC services do not interfere with other health services provided at the site.(DSD) 3. VMMC services are easily accessible to interested clients.(DSD) 4. Site-level VMMC performance data is submitted to the county health team through the national reporting system. (SI) 5. All VMMC field staff are trained and certified to perform their duties as assigned.(HRH) | 1. *Recruitment of clients for VMMC does not interfere with any services provided at the recruitment venues.(DSD)* 2. VMMC services are offered in a manner acceptable to the community (DSD) 3. The county health team uses data to measure the effectiveness of the VMMC project in delivering needed VMMC services in the right locations. (P&C) 4. Staff have the training and capacity to apply VMMC quality improvement methods. (QM) 5. Performance against VMMC targets is reviewed at least quarterly with the service delivery team. (QM) |
| Weaknesses | 1. *The county health team does not develop budgets that allocate resources to high need VMMC service delivery locations.* 2. The county health team needs external technical assistance to execute their VMMC responsibilities. 3. There are waiting lists for VMMC services. | 1. *The county health team does not develop budgets that allocate resources to high need VMMC service delivery locations.* |

Summary of key strengths and major weaknesses across domains for the static model.

**Strengths**: At baseline, strengths of the static model included Domestic Service Delivery, Planning and Coordination, Supply Chain and Quality Management. VMMC services were offered regularly and did not interfere with other services. Performance data was used to plan services and was submitted to the county health team. These strengths continued in Year 1. In addition, by Year 1, training and certification of VMMC field staff emerged as a key strength of this model.

**Weaknesses**: Major weaknesses at identified were that the VMMC program was dependent on external technical assistance and funding sources, routine VMMC service delivery was not task-shifted to the lowest permitted cadre and low levels of civil society engagement. All these contuined to be weaknesses in Year 1.

| **Tool 5: Project and Site Level _ Static Model** | | |
| --- | --- | --- |
| Section | **Baseline  (n=15)** | **Year 1 (n=14)** |
| Strengths | 1. *VMMC is offered regularly as planned (DSD).* 2. VMMC services do not interfere with other health services provided at the site. 3. *Site-level VMMC performance data is submitted to the county health team through the national reporting system* 4. Performance against VMMC targets is reviewed at least quarterly with the service delivery team.(QM) 5. Supply processes work well enough to provide everything needed to ensure client safety and achieve VMMC targets. (SC) | 1. *VMMC is offered regularly as planned*. *(DSD*) 2. VMMC services are offered in a manner acceptable to the community (DSD) 3. *Site-level VMMC performance data is submitted to the county health team through the national reporting system* 4. Performance against VMMC quality standards is reviewed at least quarterly with the service delivery team. (QM) 5. All VMMC field staff are trained and certified to perform their duties as assigned.(HRH) |
| Weaknesses | 1. *Domestic funding sources for VMMC program support have not been identified.* 2. *Routine VMMC service delivery is not task-shifted to the lowest permitted cadre.* 3. *Do not have diverse community groups that are committed to the success of the VMMC sustainability project.* 4. The county health team does not execute their VMMC responsibilities without the need for external technical assistance. | 1. *Domestic funding sources for VMMC program support have not been identified.* 2. *Routine VMMC service delivery is not task-shifted to the lowest permitted cadre* 3. *Do not have diverse community groups that are committed to the success of the VMMC sustainability project.* |

**TOOL 6: KEY INFORMANT QUESTIONNAIRE – COMMUNITY LEVEL**

This tool was completed by community members in areas where the study is being implemented. In total, 133 questionnaires were completed during the baseline and 206 during the first year. The following domains were assessed for sustainability:

1. Community Engagement and Participation (CE&P)
2. Service Delivery (SD)

Below are results of the top 5 strengths and weaknesses across all domains for all models as well as for each model (Mixed, Mobile and Static), as reported by the respondents.

**Summary of key strengths and major weaknesses across all models:**

**Strengths:** At baseline, community respondents across all models reported high-levels of community engagement in the VMMC program. Service delivery was also a strength – most people knew where MC services were offered in their community and boys 10-14 years could easily get circumcised. These strengths continued in Year 1.

**Weaknesses**: At baseline, community respondents reported low levels of community participation in planning or providing feedback to improve services and felt that some services were not well planned and coordinated. Limited civil society participation in planning or providing feedback to improve services continued to be a major weakness in Year 1.

| **Tool 6: Community Level _ All Models** | | |
| --- | --- | --- |
|  | **Baseline** | **Year 1** |
| **Strengths** | 1. *If a boy between 10-14 years old wants to get circumcised, he can easily get the service.(SD)* 2. *The information received about the male circumcision program helps people know more about the services offered in their community.(CEP)* 3. The information received about the male circumcision program helps you to know how well the program is doing. (CEP) 4. Most people know about the MC services offered in the community (SD) 5. In general, community representatives have a lot of influence in the way male circumcision services are provided in this community.(CEP) | 1. *If a boy between 10-14 years old wants to get circumcised, he can easily get the service. (SD)* 2. Boys who go to get circumcised get services on the same day. (SD*)* 3. *The information received about the male circumcision program helps people know more about the services offered in their community. (CEP)* 4. Feedback provided by community representatives is used to improve the way male circumcision services are provided in this community.   5. The Ministry of Health shares important information about the male circumcision program in your community. |
| **Weaknesses** | 1. *Community representatives do not often participate in meetings with the Ministry of Health to provide feedback about male circumcision services being offered in their community.* 2. *Community representatives do not often participate in meetings with the Ministry of Health to plan the male circumcision services being offered in your community.* 3. Community representatives do not participate in activities to recruit boys for male circumcision in your community. 4. MC activities conducted in the community are not planned and coordinated in a way that they do no disrupt services. | 1. *Community representatives do not often participate in meetings with the Ministry of Health to provide feedback about male circumcision services being offered in their community.* 2. *Community representatives do not often participate in meetings with the Ministry of Health to plan the male circumcision services being offered in your community.* 3. Community representatives do not often participate in meetings with other community leaders or community members to talk about the male circumcision services in your community. |

**Summary of key strengths and major weaknesses of the mixed model:**

**Strengths:** At baseline, community respondents in the mixed model areas were pleased with VMMC Service delivery – boys 10-14 years seeking to get circumcised could easily get the service on the day that they went to seek male circumcision services. They also reported that community engagement through community representatives was very high. These strengths continued in Year 1. In addition, community respondents were well informed about VMMC services in their community.

**Weaknesses**: At baseline, although community engagement through community representatives was important, respondents reported low levels of community participation in planning services, providing feedback and recruting biys for MC. Limited civil society engagement in planning or providing feedback to improve services conituned to be a major weakness in Year 1.

| **Tool 6: Community Level_Mixed Model** | | |
| --- | --- | --- |
|  | **Baseline  (n=31)** | **Year 1 (n=74)** |
| Strengths | *If a boy between 10-14 years old wants to get circumcised, he can easily get the service.*  *Boys who go to get circumcised get services on the same day.*  *The information received about the male circumcision program helps the community to know more about the services offered in the community.*  In general, community representatives have a lot of influence in the way male circumcision services are provided in this community.  In general, feedback provided by community representatives is used to improve the way male circumcision services are provided in this community. | *If a boy between 10-14 years old wants to get circumcised, he can easily get the service.*   1. *Boys who go to get circumcised get services on the same day.* 2. *The information received about the male circumcision program helps them to know more about the services offered in the community*. 3. The Ministry of Health shares important information about the male circumcision program in your community. 4. The information received about the male circumcision program helps the community to know how well the program is doing. |
| Weaknesses | 1. *Community members do not often participate in meetings with the Ministry of Health to provide feedback about male circumcision services being offered in the community.* 2. Community members do not often participate in meetings with the Ministry of Health to plan the male circumcision services being offered in your community. 3. Community members do not participate in activities to recruit boys for male circumcision in your community. | 1. *Community representatives do not often participate in meetings with the Ministry of Health to provide feedback about male circumcision services being offered in the community.* 2. Community representatives do not often participate in meetings with the Ministry of Health to plan the male circumcision services being offered in the community. 3. Community representatives do not often participate in meetings with other community leaders or community members to talk about the male circumcision services in the community. |

**Summary of key strengths and major weaknesses of the mobile model:**

**Strengths:** At baseline, community respondents in the mixed model areas were pleased with VMMC Service delivery – boys 10-14 years seeking to get circumcised could easily get the service on the day that they went to seek male circumcision services. They also reported that community engagement through community representatives was very high. These strengths continued in Year 1. In addition, community respondents were well informed about VMMC services in their community.

**Weaknesses**: At baseline, although community engagement through community representatives was important, respondents reported low levels of community participation in planning services, providing feedback and recruiting boys for MC. Limited civil society engagement in planning or providing feedback to improve services continued to be a major weakness in Year 1.

| **Tool 6: Community Level _ Mobile Model** | | |
| --- | --- | --- |
| Section | **Baseline  (n=69)** | **Year 1 (n=82)** |
| Strengths | 1. In general, community representatives have a lot of influence in the way male circumcision services are provided in this community. 2. In general, feedback provided by community representatives is used to improve the way male circumcision services are provided in this community. 3. The information you receive about the male circumcision program helps you to know more about the services offered in my community. 4. The information you receive about the male circumcision program helps you to know how well the program is doing. 5. Most people know about the MC services offered in the community. 6. If a boy between 10-14 years old wants to get circumcised, he can easily get the service. | 1. If a boy between 10-14 years old wants to get circumcised, he can easily get the service. 2. Boys who go to get circumcised get services on the same day. 3. The information received about the male circumcision program helps people know more about the services offered in their community. 4. Most people know about the MC services offered in the community.   In general, community representatives are willing to support the male circumcision program in this community. |
| Weaknesses | 1. Community members do not often participate in meetings with the Ministry of Health to provide feedback about male circumcision services being offered in their community. 2. Community members do not ofte participate in meetings with the Ministry of Health to plan the male circumcision services being offered in your community.   3. Community representatives do not participate in activities to recruit boys for male circumcision in your community. | 1. Community members do not often participate in meetings with the Ministry of Health to provide feedback about male circumcision services being offered in their community. 2. Community members do not often participate in meetings with the Ministry of Health to plan the male circumcision services being offered in your community. |

**Summary of key strengths and major weaknesses of the static model:**

**Strengths:** At baseline, community respondents in the static model areas were pleased with VMMC Service delivery – boys 10-14 years seeking to get circumcised could easily get the service on the day that they went to seek male circumcision services. They also reported that the Ministry of Health kept them informed about VMMC services in their community. These strengths continued in Year 1.

**Weaknesses**: A major weakness at baseline as well as Year 1 was limited community participation in planning services or providing feedback to improve services.

| **Tool 6: Community Level _ Static Model** | | |
| --- | --- | --- |
| Section | **Baseline  (n=33)** | **Year 1 (n=50)** |
| **Strengths** | 1. *If a boy between 10-14 years old wants to get circumcised, he can easily get the service.* 2. *Boys who go to get circumcised get services on the same day.* 3. *The information received about the male circumcision program helps you to know more about the services offered in their community.* 4. The information received about the male circumcision program helps you to know how well the program is doing. 5. Most people know about the MC services offered in the community. | 1. *If a boy between 10-14 years old wants to get circumcised, he can easily get the service.* 2. *Boys who go to get circumcised get services on the same day.* 3. *The information received about the male circumcision program helps people know more about the services offered in their community.* 4. In general, feedback provided by community representatives is used to improve the way male circumcision services are provided in this community.   5. The Ministry of Health shares important information about the male circumcision program in your community. |
| **Weaknesses** | 1. *Community representatives do not often participate in meetings with the Ministry of Health to provide feedback about male circumcision services being offered in their community.* 2. *Community representatives do not often participate in meetings with the Ministry of Health to plan the male circumcision services being offered in their community.* 3. Community representatives do not participate in activities to recruit boys for male circumcision in your community. | 1. *Community representatives do not often participate in meetings with the Ministry of Health to provide feedback about male circumcision services being offered in their community.* 2. *Community representatives do not often participate in meetings with the Ministry of Health to plan the male circumcision services being offered in your community*. |

1. [↑](#endnote-ref-1)
